# Mechanochemical difluoromethylations of ketones

**DOI:** 10.3762/bjoc.20.235

**Published:** 2024-11-04

**Authors:** Jinbo Ke, Pit van Bonn, Carsten Bolm

**Affiliations:** 1 Institute of Organic Chemistry, RWTH Aachen University, Landoltweg 1, 52074 Aachen, Germany

**Keywords:** ball milling, difluorocarbene, difluoromethylations, difluoromethyl enol ether, mechanochemistry

## Abstract

We present a mechanochemical synthesis of difluoromethyl enol ethers. Utilizing an in situ generation of difluorocarbenes, ketones are efficiently converted to the target products under solvent-free conditions. The reactions proceed at room temperature and are complete within 90 minutes, demonstrating both efficiency and experimental simplicity.

## Introduction

In recent years, mechanochemical organic synthesis has been advanced significantly, prompting organic chemists to reconsider the necessity of solvents in their reactions [1–11]. Eliminating hazardous solvents substantially reduces the ecological footprint of organic reactions [12–13]. Beyond environmental benefits and enhanced human safety, mechanochemical reactions often feature shorter reaction times, eliminate the need for external heating, and offer alternative product selectivity [3,14]. In general, such reactions are characterized by the absorption of mechanical energy and they are influenced by several factors, including the lack of solvation, changes in morphology and rheology of the reaction mixtures during the milling, and variations in concentration and dielectric environment. Consequently, an increased reactivity can be achieved through the formation of novel reactive intermediates [15–18].

Fluorine-containing functional groups are essential structural motifs in the development of new bioactive compounds and functional materials. Compared to their non-fluorinated analogs, the presence of fluorine atoms in molecular structures can improve physicochemical and biological properties [19–27]. Among these groups, the difluoromethyl moiety has gained considerable attention [28–30]. Commonly, it is synthesized by the reaction of a nucleophile with difluorocarbene. However, the generation of difluorocarbene typically requires harsh conditions and involves toxic precursors, alongside with the risk of dimerization to tetrafluoroethylene [31]. Although this dimerization can be mitigated by controlling the concentration and reaction environment, as longer half-lives are observed for difluorocarbenes in the gas phase than in solution [32–33], it has remained a challenge to control such reactions.

Our group has recently reported a mechanochemical difluoromethylation of primary, secondary, and tertiary alcohols [34], yielding products with difluoromethoxy groups, which are promising organofluorine compounds [35–38]. Notably, also sterically hindered alcohols, which are typically less reactive in solution, could be applied under solvent-free conditions in a ball mill [39], which was attributed to a better accessibility of the difluorocarbene in the mechanochemical environment [40].

**Scheme 1:**
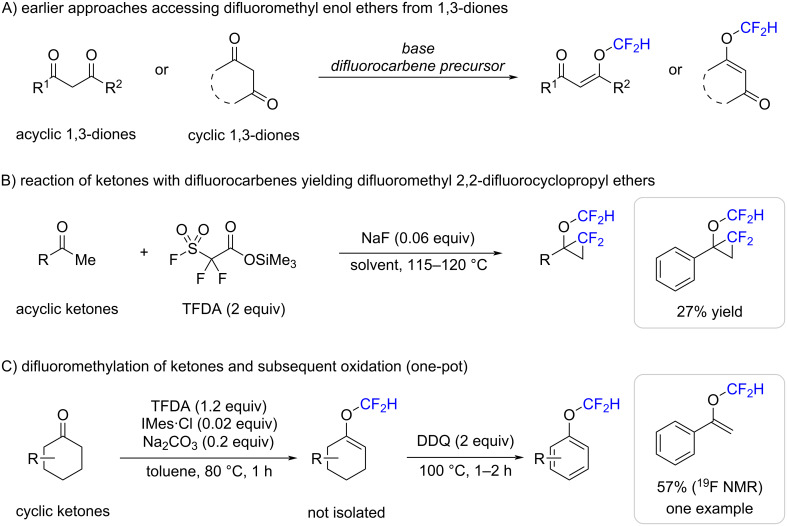
Overview over difluoromethyl enol ether syntheses from acyclic and cyclic 1,3-diones (A), acyclic ketones (B), and cyclic ketones (C).

Motivated by these findings, we now explored difluoromethylation reactions with compounds bearing less nucleophilic functional groups. Ketones caught our particular attention as they contain a weak nucleophilic carbonyl oxygen adjacent to an electrophilic carbonyl carbon. Previous studies have focused on reactions of difluorocarbene with cyclic and acyclic 1,3-diones (Scheme 1A) [41–45]. Typically, they were conducted with a base to form the corresponding enolate anions which then reacted with difluorocarbene to yield difluoromethyl enol ethers. Those products are of interest because they contain a unique structural motif with potential for further functionalizations into highly diverse secondary or tertiary difluoroalkyl ethers. Dolbier and co-workers investigated reactions of difluorocarbene generated from its precursor trimethylsilyl 2,2-difluoro-2-(fluorosulfonyl)acetate (TFDA) and sodium fluoride catalyst, with simple ketones, which resulted in the formation of difluoromethyl 2,2-difluorocyclopropyl ethers (Scheme 1B). Although the reactions worked well, it is also noteworthy that the use of TFDA as reagent, liberated fluoro(trimethyl)silane (TMSF), carbon dioxide, and ozone-depleting sulfur dioxide as side products [46–47]. Later, Ichikawa and co-workers established the release of difluorocarbene from TFDA with catalytic amounts of an *N*-heterocyclic carbene and a base (Scheme 1C) [29,48–49]. In these reactions, difluoromethyl enol ethers were obtained, which were subsequently oxidized to yield the corresponding aryl difluoromethyl ethers. Noteworthy, however, the latter study focused mostly on cyclic ketones, with only one reported example of a difluoromethylation reaction of an acyclic substrate.

Against this background and seeing new synthetic opportunities, we wondered about reactions of mechanochemically generated difluorocarbene with simple acyclic ketones. The results and observations of this study are summarized here.

## Results and Discussion

For the optimization of the reaction conditions, 4-methylacetophenone (**1a**) was chosen as model substrate. Under standard reaction conditions with difluorocarbene precursor TMSCF_2_Br (**2**, 2.0 equiv), activator KFHF (4.0 equiv), and grinding auxiliary CsCl (4.0 equiv), difluoromethyl enol ether **3a** was obtained after 90 min reaction time at 25 Hz in 74% yield, determined by quantitative ^1^H NMR spectroscopy (Table 1, entry 1). The reaction was conducted in a PTFE milling equipment with two milling balls (diameter: 10 mm). Changing to a heavier milling ball (diameter: 15 mm) resulted in a yield of 67% of **3a** (Table 1, entry 2). Stopping the reaction after 60 min gave product **3a** in 72% yield (Table 1, entry 3). At a reaction time of 60 min, both reducing and increasing the amount of **2** (from initially used 2.0 equiv to 1.5 equiv and 3.0 equiv, respectively) reduced the yield of **3a** by about 10% (Table 1, entries 3–5). Probably, with less carbene precursor the amount of generated difluorocarbene was insufficient, and with too much of it, side reactions occurred [31–33]. Next, various grinding auxiliaries were investigated at a reaction time of 60 min (Table 1, entries 6–9). A similar yield of **3a** (69%) was obtained with KCl or KBr instead of CsCl (Table 1, entries 6 and 7 versus entry 3). Using NaCl, gave **3a** in 64% yield (Table 1, entry 8). Finally, CsCl was substituted by silica, which, to our surprise, blocked the product formation completely (Table 1, entry 9). Apparently, the presence of an alkali halide salt was beneficial, most likely by stabilizing the consistency of the reaction mixture leading to a sufficient mixing. Silica could not fulfill this role. Lastly, water, 1,4-dioxane, chloroform, and toluene were tested in a liquid-assisted grinding (LAG) protocol (Table 1, entries 10–13). The lowest yields were obtained with water and 1,4-dioxane, providing **3a** in yields of 37% and 43%, respectively (Table 1, entries 10 and 11). With the less polar solvents chloroform and toluene **3a** was obtained in 62% and 68% yield, respectively (Table 1, entries 12 and 13).

**Table 1:** Optimization of the reaction conditions.^a^

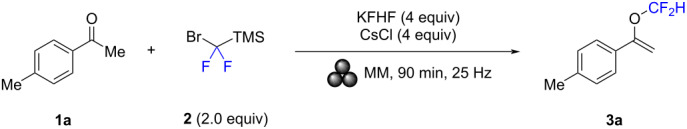		

Entry	Deviation from the reaction conditions	Yield of **3a** (%)^b^

1	none	74^c^
2	with one PTFE milling ball (diameter: 15 mm)	67
3	60 min	72
4	60 min, **2** (1.5 equiv)	61
5	60 min, **2** (3.0 equiv)	60
6	60 min, KCl instead of CsCl	69
7	60 min, KBr instead of CsCl	69
8	60 min, NaCl instead of CsCl	64
9	60 min, SiO_2_ instead of CsCl	0
10	LAG (H_2_O)	37
11	LAG (1,4-dioxane)	43
12	LAG (CHCl_3_)	62
13	LAG (toluene)	68

^a^Reaction performed with two PTFE milling balls (diameter: 10 mm) in a PTFE jar (volume: 25 mL). Liquid-assisted grinding (LAG): 0.25 µL·mg^−1^. ^b^Determined by ^1^H NMR spectroscopy using 1,2-dichloroethane as the internal standard. ^c^Repetition of the experiment gave consistent results.

**Scheme 2:**
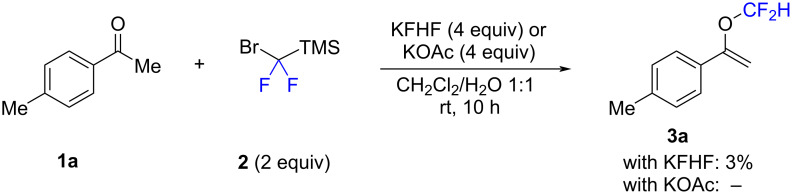
Attempted difluoromethylation of **1a** in solution. The reactions were performed on a 0.2 mmol scale. Method A: **2** (2.0 equiv), KFHF (4.0 equiv), CH_2_Cl_2_ (0.2 mL), H_2_O (0.2 mL), rt, 10 h; Method B: **2** (2.0 equiv), KOAc (4.0 equiv), CH_2_Cl_2_ (0.2 mL), H_2_O (0.2 mL), rt, 10 h. The yields were determined by ^19^F NMR spectroscopy using trifluoromethoxybenzene as the internal standard.

For comparison, the difluoromethylation of ketone **1a** with difluorocarbene precursor TMSCF_2_Br (**2**) was investigated in solution (Scheme 2). The reaction conditions were chosen based on those reported by Ni, Hu and co-workers for the difluoromethylation of alcohols in solution [39]. The two activators KFHF and KOAc were investigated in a dichloromethane/water mixture at room temperature for 10 h. In both cases, the yield of **3a** was negligible (with KFHF: 3%, with KOAc: nil). Following these initial attempts, the mechanochemical approach appears to be superior. However, it should also be noted that the reaction with difluorocarbene precursor **2** was not further optimized in solution.

**Scheme 3:**
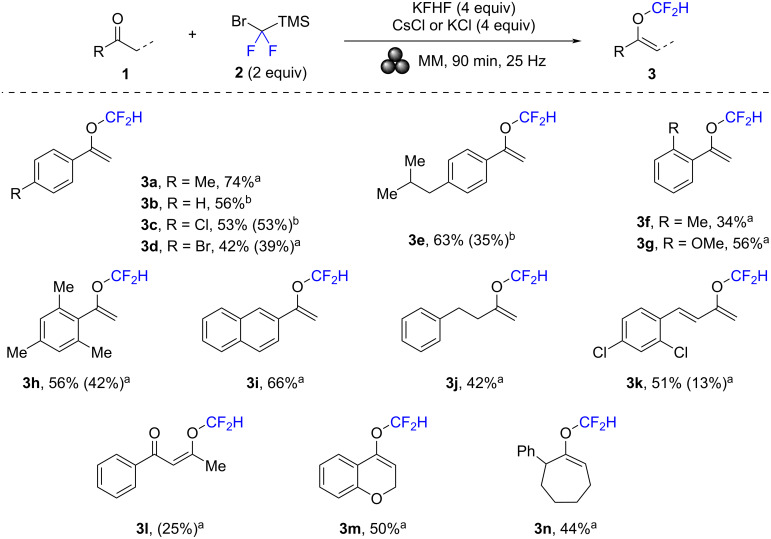
Scope of ketones. The yields were determined by ^1^H NMR spectroscopy using 1,2-dichloroethane as the internal standard. In parentheses: yields after column chromatography (with product purities of ca. 90%). ^a^With CsCl. ^b^With KCl.

Next, various ketones were investigated under the optimized reaction conditions with difluorocarbene precursor **2**, KFHF (4 equiv) as activator, and CsCl or KCl (4 equiv) as grinding auxiliaries in a PTFE milling jar for 90 min at 25 Hz (Scheme 3). To get an initial efficiency estimate, the crude reaction mixtures were first analyzed by quantitative ^1^H NMR spectroscopy with 1,2-dichloroethane as the internal standard. After these analyses, isolating the products by column chromatography was attempted. Unfortunately, many products were highly volatile and very non-polar, rendering the purification difficult. As a result, in several cases only little or no product was obtained. Furthermore, most isolated products had only purities of ca. 90% still containing inseparable impurities (as revealed by NMR spectroscopy).

In the first series of substrates, acetophenone derivatives with various *para*-substituents were applied. Similar to methyl tolyl ketone (**1a**), which afforded product **3a** in 74% yield, acetophenone (**1b**) gave **3b** in 56% yield. Substrates **1c** and **1d** bearing a chloro or a bromo group in *para* position of the aryl moiety, gave the corresponding products in yields of 53% (for **3c**) and 42% (for **3d**). These two difluoromethyl enol ethers were also isolated by column chromatography, which afforded the products in 53% and 39% yield, respectively. Product **3e** with an isobutyl group in *para*-position was obtained in 63% yield and isolated in 35% yield. Changing the position of the methyl group to the *ortho*-position led to a decrease in yield (**3f**: 34%). *ortho*-Methoxy-substituted ketone **1g** provided the corresponding product **3g** in 56% yield. Difluoromethyl enol ether **3h** with three methyl groups was obtained in 56% yield and column chromatography allowed to isolate it in 42% yield. 2-Acetonaphthone was successfully converted to **3i** in 66% yield. Besides aryl ketones, arylalkyl ketones reacted well too. Accordingly, **3j** was obtained in 42% yield. Enone **1k** gave **3k** in 51% yield, and after isolation by column chromatography the product was obtained in 13% yield. Difluoromethyl enol ether **3l** was formed from diketone **1l** in 25% yield. Finally, conversions of the two cyclic ketones **1m** and **1n** were studied. Both gave the expected products in yields of 50% (for **3m**) and 44% (for **3n**).

Besides these successful transformations several ketones proved unsuitable (Scheme S1 in Supporting Information File 1). Additionally, attempted [4 + 1]-type cycloadditions of three 1-arylprop-2-en-1-ones as heteroconjugated alkenes with difluorocarbene to give 2,2-difluoro-2,3-dihydrofurans [50] remained unsuccessful (Scheme S2 in Supporting Information File 1).

**Scheme 4:**
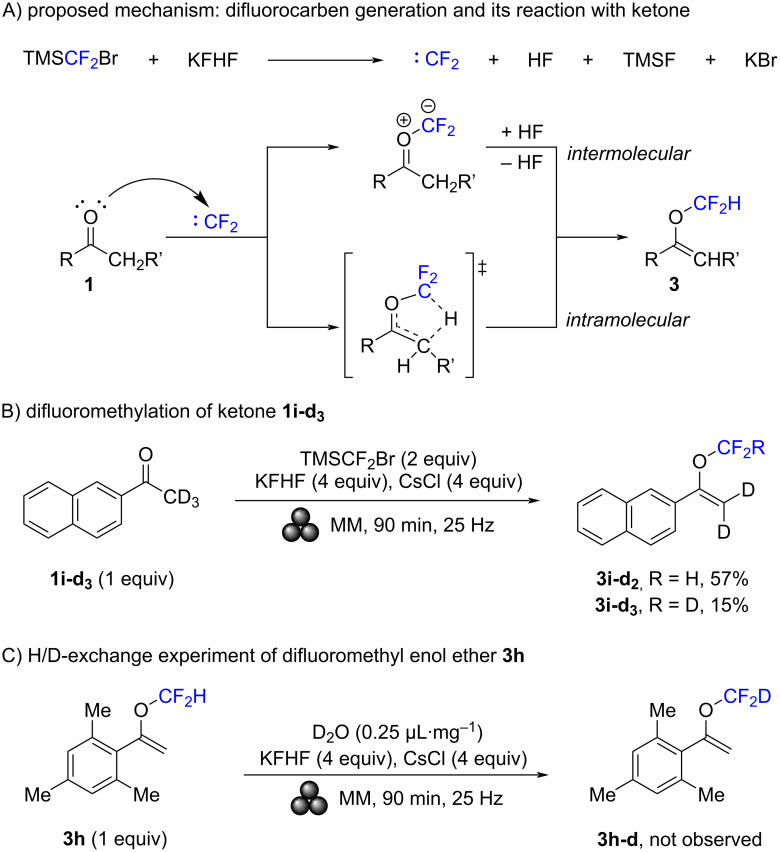
Proposed mechanism (A) and mechanistic investigations (B and C). The yields were determined by ^1^H NMR spectroscopy using 1,2-dichloroethane as internal standard.

Two mechanisms have been proposed for the difluoromethylation of ketones, as illustrated in Scheme 4A. In both cases, the process begins with the generation of difluorocarbene from TMSCF_2_Br and KFHF. This is followed by a nucleophilic attack of the oxygen atom of ketone **1** on the difluorocarbene. Subsequently, a protonation–deprotonation sequence occurs, which can either be intermolecular, involving a molecule of HF, or intramolecular, proceeding through a five-membered transition state.

To clarify the mechanism, two experiments were conducted. In the first one, 2-acetonaphthone with a trideuteromethyl group (**1i-d_3_**) was subjected to the standard reaction conditions. Two products were obtained: First, **3i-d_2_** containing a CF_2_H group, and second **3i-d_3_** bearing a CF_2_D group. The yields were 57% and 15%, respectively. In the second experiment, the potential for proton exchange in difluoromethyl enol ether **3h** was investigated. The compound was milled with the activator KFHF, CsCl as grinding auxiliary, and D_2_O in a liquid-assisted grinding process. As a result, no H/D-exchange was detected. The experimental results of both experiments suggest that the reaction predominantly proceeds through an intermolecular pathway. The occurrence of the CF_2_D product may be attributed to a minor intramolecular reaction path or the involvement of DF formed during the reaction.

## Conclusion

In conclusion, we discovered a mechanochemical synthesis of difluoromethyl enol ethers. The products were obtained from the corresponding ketones at room temperature after a reaction time of 90 minutes. The investigation of the reaction scope revealed challenges in isolating the low-boiling non-polar products. Mechanistic studies suggested that in situ-generated difluorocarbene reacts with the ketone oxygen, followed by intermolecular protonation/deprotonation. Although the process has still synthetic limitations, also acyclic ketones can now be converted into difluoromethyl enol ethers, which have the potential for further functionalization.

## Supporting Information

File 1 Experimental procedures, optimization studies, compound characterization data, NMR spectra, and mechanistic investigations.

## Data Availability

All data that supports the findings of this study is available in the published article and/or the supporting information of this article.
