# Supplementary material for: Mechanochemical difluoromethylations of ketones
Source: Beilstein J Org Chem. 2024 Nov 4;20:2799–805. doi: 10.3762/bjoc.20.235 (PMC11552444; doi:10.3762/bjoc.20.235)

BEILSTEIN JOURNAL OF ORGANIC CHEMISTRY

Supporting Information

for

Mechanochemical difluoromethylations of ketones

Jinbo Ke, Pit van Bonn and Carsten Bolm

Beilstein J. Org. Chem. **2024,** *20,* 2799–2805. doi:10.3762/bjoc.20.235

Experimental procedures, optimization studies, compound characterization data, NMR spectra, and mechanistic investigations

License and Terms: This is a supporting information file under the terms of the Creative Commons Attribution License (https://creativecommons.org/licenses/by/4.0). Please note that the reuse, redistribution and reproduction in particular requires that the author(s) and source are credited and that individual graphics may be subject to special legal provisions.
The license is subject to the *Beilstein Journal of Organic Chemistry* terms and conditions: (https://www.beilstein-journals.org/bjoc/terms)

Table of contents

1.	General information	S2
2.	Investigation of various difluorocarbene precursors	S3
3.	Difluoromethylations in solution	S4
4.	Mechanochemical difluoromethylations of ketones	S4
5.	Analytical data of the products	S4
6.	Unsuccessful difluoromethylations	S11
7.	Mechanistic investigations	S12
8.	References	S20
9.	NMR spectra	S21

1. General information

Concentration under reduced pressure was performed by rotary evaporation at 40 °C at an appropriate pressure, unless otherwise stated. All chemicals used were purchased from commercial suppliers and used without further purification unless mentioned. 1-([1,1'-Biphenyl]-4-yl)ethan-1-one [1], 1-(4-phenoxyphenyl)ethan-1-one [2], 1-(6-methoxynaphthalen-2-yl)ethan-1-one [3], 1-(*p*-tolyl)prop-2-en-1-one [4], and 1-(naphthalen-2-yl)ethan-1-one-2,2,2-d_3 [5] were prepared according to procedures reported in literature.

Mechanochemical reactions were conducted using a RETSCH Mixer Mill MM 400. PTFE milling vessels (volume: 25 mL) and PTFE milling balls (diameters: 10 mm or 15 mm) were utilized.

Solvents used in the reactions were obtained commercially and used without additional purification. For column chromatographic purification, technical-grade *n*-pentane and acetone were employed, with *n*-pentane further purified by distillation. The deuterated solvent $CDCl_3$ was purchased commercially, dried through filtration over aluminum oxide and stored over molecular sieves 4 Å.

Purifications by column chromatography were performed using silica gel 60 (particle size 0.04–0.063 mm). The running solvents are indicated as volume ratios (*V*/*V*). Thin-layer chromatography was used for reaction control, employing silica gel-covered aluminum foils with a fluorescent indicator (Merck, DC Alumina 60 F254, neutral). The compounds were detected under UV light (λ = 254 nm and 366 nm) and with aqueous $KMnO_4$ stain solution and subsequent heating.

^{1}H, ^{13}C, and ^{19}F NMR spectra were recorded on a Brucker Avance Neo 400 (400 MHz), Bruker Avance Neo 600 (600 MHz), Varian VNMRS 400 (400 MHz), or Varian VNMRS 600 (600 MHz) spectrometer at ambient temperature. Chemical shifts (δ) are reported in parts per million (ppm) relative to the residual signal of the solvent used. Coupling constants (*J*) are given in hertz (Hz). Signal multiplicities are abbreviated as follows: s = singlet, d = doublet, dd = doublet of doublets, dt = doublet of triplets, t = triplet, td = triplet of doublets, m = multiplet.

Infrared (IR) spectra were recorded on a PerkinElmer Spectrum 100 FT-IR spectrometer equipped with a UATR device featuring a KRS-5 crystal for single reflection.

High-resolution mass spectra (HRMS) were recorded on a Thermo Scientific LTQ Orbitrap XL spectrometer.

2. Investigation of various difluorocarbene precursors

A PTFE milling jar (volume: 25 mL) equipped with one milling ball of the same material (diameter: 12 mm) was loaded with 4'-methylacetophenone (**1a**, 28.1 μL, 0.200 mmol, 1.00 equiv), difluorocarbene precursor, activator, and additive. The jar was closed in air, transferred to a mixer mill, and shaken for 1 h. After the milling, the jar was opened in air, and a stock solution of dichloroethane (0.200 mmol, 1.00 equiv) as the internal standard in $CDCl_3$ was added. The resulting suspension was filtered over cotton, and the yield of the product **3a** was determined by quantitative ^1H NMR spectroscopy.

Table S1: Investigation of different difluorocarbene precursors in the difluoromethylation of ketone **1a**.[a]

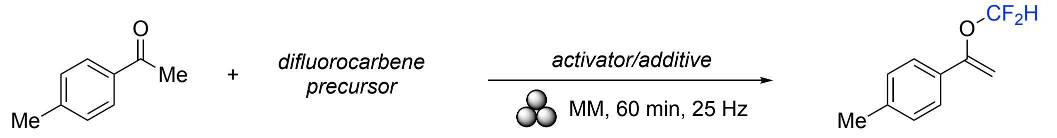

1a **3a**

difluorocarbene precursor:

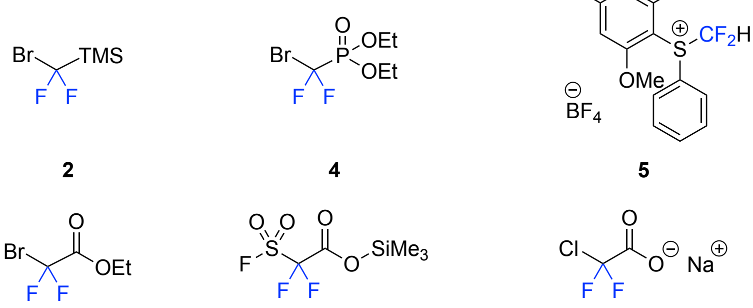

2 **4** **5** **6** **7** **8**

Entry	Ref.	Difluorocarbene precursor (equiv)	Activator/additive (equiv)	Yield of **3a** (%)[b]
1	[6]	**2** (2.0)	KFHF (4.0), KCl (4.0)	69
2	[7]	**4** (2.0)	K_3PO_4 (8.0)	0
3	[8]	**5** (2.0)	–	0
4	[9]	**6** (2.0)	Cs_2CO_3 (8.0)	0
5	[10]	**7** (1.5)	KF (4.0)	trace
6	[11]	**8** (2.0)	Cs_2CO_3 (4.0)	0
7	–	–	KFHF (4.0), KCl (4.0)	0

[a]Performed with one PTFE milling ball (diameter: 12 mm). [b]Determined by ^1H NMR spectroscopy using 1,2-dichloroethane as the internal standard.

3. Difluoromethylations in solution

The difluoromethylations of ketone **1a** were carried out under slightly modified conditions **A** or **B** according to procedures reported in literature [12]. 4'-Methylacetophenone (**1a**, 28.1 µL, 0.200 mmol, 1.00 equiv), KFHF (**A**, 62.5 mg, 0.800 mmol, 4.00 equiv) or KOAc (**B**, 80.9 mg, 0.800 mmol, 4.00 equiv), CH_2Cl_2 (0.2 mL) and H_2O (0.2 mL) were added into a PTFE tube (10 mL) with a cap. Then, the reaction mixture was stirred at room temperature and $TMSCF_2Br$ (**2**, 65.5 µL, 0.400 mmol, 2.00 equiv) was added. After 10 h the reaction mixture was diluted with CH_2Cl_2 (5 mL), and $PhOCF_3$ (40.1 µL, 0.300 mmol, 1.5 equiv) was added as the internal standard. The organic layer was analyzed by ^{19}F NMR spectroscopy.

4. Mechanochemical difluoromethylations of ketones

A PTFE milling jar (25 mL) equipped with two PTFE milling balls (diameter: 10 mm) was loaded with ketone **1** (0.200 mmol, 1.00 equiv), activator KFHF (62.5 mg, 0.800 mmol, 4.00 equiv), and the chloride salt [**GP1**: CsCl (136 mg, 0.800 mmol, 4.00 equiv); **GP2**: KCl (59.6 mg, 0.800 mmol, 4.00 equiv)]. Subsequently, $TMSCF_2Br$ (**2**, 65.5 µL, 0.400 mmol, 2.00 equiv) was added, and the jar was tightly closed. After milling for 90 min at 25 Hz, the reaction mixture was suspended with a stock solution of 1,2-dichloroethane (0.200 mmol, 1.00 equiv) as the internal standard in $CDCl_3$. The resulting solution was filtered over cotton, and the yield of the product was determined by ^1H NMR spectroscopy. The jar was extracted with acetone (3 × 2 mL) and the combined organic phase filtered over cotton and concentrated. Product **3** was obtained after purification by flash column chromatography on silica gel and evaporation of the solvent.

5. Analytical data of the products

1-[1-(Difluoromethoxy)vinyl]-4-methylbenzene (3a)

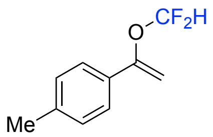

Following the **GP1**, difluoromethyl enol ether **3a** was obtained from 4'-methylacetophenone (**1a**, 28.1 µL, 0.200 mmol, 1.00 equiv) in 74% yield determined by ^1H NMR spectroscopy.

NMR yield: 74% [δ_{DCE} = 3.73 (s, 4H) ppm; $\delta_{product}$ = 5.08 (d, J = 3.3 Hz, 1H), 4.69 (d, J = 3.3 Hz, 1H) ppm].

^{1}H NMR (600 MHz, $CDCl_3$): δ = 7.49 (d, J = 8.3 Hz, 2H), 7.18 (d, J = 8.0 Hz, 2H), 6.52 (t, ^2J_F = 74.2 Hz, 1H), 5.08 (d, J = 3.3 Hz, 1H), 4.69 (d, J = 3.3 Hz, 1H), 2.37 (s, 3H) ppm.

^{19}F NMR (565 MHz, $CDCl_3$): δ = –81.39 (d, J = 74.3 Hz, 2F) ppm.

^{13}C NMR (151 MHz, $CDCl_3$): δ = 154.4, 139.5, 130.8, 129.2, 125.2, 115.9 (t, ^1J_F = 256.7 Hz), 92.2, 21.2 ppm.

1-(Difluoromethoxy)vinyl]benzene (3b)

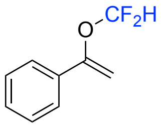

Following the **GP2**, difluoromethyl enol ether **3b** was obtained from acetophenone (**1b**, 23.3 µL, 0.200 mmol, 1.00 equiv) in 56% yield determined by ^{1}H NMR spectroscopy.
NMR yield: 56% [δ_{DCE} = 3.73 (s, 4H) ppm; $\delta_{product}$ = 5.14 (dd, J = 3.4, 0.9 Hz, 1H), 4.75 (dd, J = 3.4, 0.9 Hz, 1H) ppm].
^{1}H NMR (600 MHz, $CDCl_3$): δ = 7.62 – 7.56 (m, 2H), 7.42 – 7.34 (m, 3H), 6.53 (t, ^2J_F = 74.0 Hz, 1H), 5.14 (dd, J = 3.4, 0.9 Hz, 1H), 4.75 (dd, J = 3.4, 0.9 Hz, 1H) ppm.
^{19}F NMR (565 MHz, $CDCl_3$): δ = –81.56 (d, J = 74.1 Hz, 2F) ppm.
^{13}C NMR (151 MHz, $CDCl_3$): δ = 154.3, 133.6, 129.5, 128.5, 125.3, 115.8 (t, ^1J_F = 256.7 Hz), 93.1 ppm.
IR (neat): $\tilde{\nu}$ = 2925, 2845, 2158, 1686, 1636, 1578, 1495, 1446, 1382, 1276, 1126, 1049, 963, 919, 846, 769, 689 cm^{-1}.
HRMS (EI): m/z calculated for $C_9H_8OF_2$: 170.0543 [M]$^+$, found 170.0538.
The NMR spectra are in accordance with the literature [13].

1-Chloro-4-[1-(difluoromethoxy)vinyl]benzene (3c)

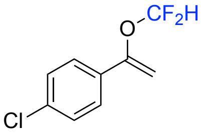

Following the **GP2**, difluoromethyl enol ether **3c** was obtained from 4'-chloroacetophenone (**1c**, 26.7 µL, 0.200 mmol, 1.00 equiv) in 53% yield (21.9 mg, 0.107 mmol). Eluent: *n*-pentane [R_F = 0.43].
^{1}H NMR (600 MHz, $CDCl_3$): δ = 7.52 (d, J = 8.7 Hz, 2H), 7.34 (d, J = 8.7 Hz, 2H), 6.52 (t, ^2J_F = 73.7 Hz, 1H), 5.12 (d, J = 3.7 Hz, 1H), 4.76 (d, J = 3.6 Hz, 1H) ppm.
^{19}F NMR (565 MHz, $CDCl_3$): δ = –81.77 (d, J = 74.3 Hz, 2F) ppm.
^{13}C NMR (101 MHz, $CDCl_3$): δ = 153.2, 135.4, 132.1, 128.7, 126.6, 115.7 (t, ^1J_F = 252.5 Hz), 93.3 ppm.
IR (neat): $\tilde{\nu}$ = 2927, 2856, 2160, 1909, 1633, 1596, 1491, 1384, 1359, 1282, 1129, 1051, 964, 907, 833, 791, 731, 662 cm^{-1}.
HRMS (EI): m/z calculated for $C_9H_7OClF_2$: 204.0154 [M]$^+$, found 204.0148.

1-Bromo-4-[1-(difluoromethoxy)vinyl]benzene (3d)

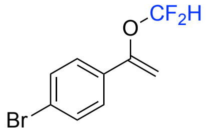

Following the **GP1**, difluoromethyl enol ether **3d** was obtained from 4'-bromoacetophenone (**1d**, 40.6 mg, 0.200 mmol, 1.00 equiv) in 39% yield (19.4 mg, 0.0779 mmol). Eluent: *n*-pentane [R_F = 0.43].

^{1}H NMR (600 MHz, $CDCl_3$): δ = 7.51 (d, *J* = 8.7 Hz, 2H), 7.46 (d, *J* = 8.7 Hz, 2H), 6.52 (t, ^2J_F = 73.7 Hz, 1H), 5.13 (d, *J* = 3.5 Hz, 1H), 4.77 (d, *J* = 3.5 Hz, 1H) ppm.

^{19}F NMR (565 MHz, $CDCl_3$): δ = –81.76 (d, *J* = 73.4 Hz, 2F) ppm.

^{13}C NMR (101 MHz, $CDCl_3$): δ = 153.3, 132.5, 131.6, 126.9, 123.7, 115.7 (t, ^1J_F = 252.5 Hz), 93.4 ppm.

IR (neat): $\tilde{v}$ = 2926, 2855, 2299, 2113, 1906, 1687, 1636, 1590, 1487, 1384, 1278, 1128, 1053, 1009, 964, 830, 784, 723, 663 cm^{-1}.

HRMS (EI): m/z calculated for $C_9H_7OBrF_2$: 247.9648 [M]$^+$, found 247.9643.

1-[1-(Difluoromethoxy)vinyl]-4-isobutylbenzene (3e)

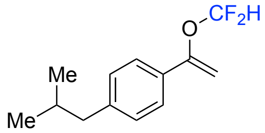

Following the **GP2**, difluoromethyl enol ether **3e** was obtained from 4'-isobutylacetophenone (**1e**, 37.5 µL, 0.200 mmol, 1.00 equiv) in 35% yield (16.0 mg, 0.0707 mmol). Eluent: *n*-pentane [R_F = 0.58].

^{1}H NMR (600 MHz, $CDCl_3$): δ = 7.50 (d, *J* = 7.5 Hz, 2H), 7.15 (d, *J* = 7.9 Hz, 2H), 6.52 (t, ^2J_F = 74.1 Hz, 1H), 5.09 (d, *J* = 3.3 Hz, 1H), 4.70 (d, *J* = 3.3 Hz, 1H), 2.49 (d, *J* = 7.2 Hz, 2H), 1.92 – 1.82 (m, 1H), 0.90 (d, *J* = 6.6 Hz, 6H) ppm.

^{19}F NMR (565 MHz, $CDCl_3$): δ = –81.37 (d, *J* = 74.4 Hz, 2F) ppm.

^{13}C NMR (101 MHz, $CDCl_3$): δ = 154.5, 143.4, 131.0, 129.2, 125.1, 115.9 (t, ^1J_F = 252.5 Hz), 92.3, 45.1, 30.2, 22.3 ppm.

IR (neat): $\tilde{v}$ = 3438, 2959, 2925, 2871, 2253, 1917, 1736, 1634, 1567, 1511, 1464, 1383, 1278, 1134, 1056, 964, 909, 846, 803, 734, 655, 534 cm^{-1}.

HRMS (EI): m/z calculated for $C_{13}H_{16}OF_2$: 226.1169 [M]$^+$, found 226.1164.

1-[1-(Difluoromethoxy)vinyl]-2-methylbenzene (3f)

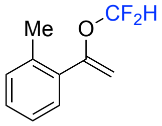

Following the **GP1**, difluoromethyl enol ether **3f** was obtained from 2'-methylacetophenone (**1f**, 26.2 µL, 0.200 mmol, 1.00 equiv) in 34% yield determined by ^{1}H NMR spectroscopy.

NMR yield: 34% [δ_{DCE} = 3.73 (s, 4H) ppm; $\delta_{product}$ = 4.89 (d, *J* = 2.7 Hz, 1H), 4.68 (d, *J* = 2.8 Hz, 1H) ppm].

^{1}H NMR (600 MHz, $CDCl_3$): δ = 7.33 (dd, *J* = 7.5, 1.5 Hz, 1H), 7.31 – 7.27 (m, 1H), 7.23 – 7.17 (m, 2H), 6.44 (t, $^{2}J_F$ = 74.2 Hz, 1H), 4.89 (d, *J* = 2.7 Hz, 1H), 4.68 (d, *J* = 2.8 Hz, 1H), 2.38 (s, 3H) ppm.
^{19}F NMR (564 MHz, $CDCl_3$): δ = –82.77 (d, *J* = 74.3 Hz, 2F) ppm.
^{13}C NMR (151 MHz, $CDCl_3$): δ = 155.4, 136.5, 132.0, 131.5, 130.6, 129.4, 125.8, 115.3 (t, $^{1}J_F$ = 256.7 Hz), 97.0, 20.1 ppm.

1-[1-(Difluoromethoxy)vinyl]-2-methoxybenzene (3g)

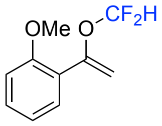

Following the **GP1**, difluoromethyl enol ether **3g** was obtained from 2'-methoxyacetophenone (**1g**, 27.6 µL, 0.200 mmol, 1.00 equiv) in 56% yield determined by ^{1}H NMR spectroscopy.
NMR yield: 56% [$δ_{DCE}$ = 3.73 (s, 4H) ppm; $δ_{product}$ = 5.23 (dt, *J* = 2.2, 1.1 Hz, 1H), 4.99 (dd, *J* = 2.2, 1.0 Hz, 1H) ppm].
^{1}H NMR (600 MHz, $CDCl_3$): δ = 7.47 (dd, *J* = 7.6, 1.8 Hz, 1H), 7.36 – 7.33 (m, 1H), 6.99 – 6.96 (m, 1H), 6.94 (dd, *J* = 8.3, 1.1 Hz, 1H), 6.45 (t, $^{2}J_F$ = 74.7 Hz, 1H), 5.23 (dt, *J* = 2.2, 1.1 Hz, 1H), 4.99 (dd, *J* = 2.2, 1.0 Hz, 1H), 3.87 (s, 3H) ppm.
^{19}F NMR (564 MHz, $CDCl_3$): δ = –80.57 (d, *J* = 74.6 Hz, 2F) ppm.
^{13}C NMR (151 MHz, $CDCl_3$): δ = 157.3, 152.1, 130.6, 129.4, 122.9, 120.5, 116.3 (t, $^{1}J_F$ = 256.7 Hz), 111.2, 99.7, 55.6 ppm.

2-[1-(Difluoromethoxy)vinyl]-1,3,5-trimethylbenzene (3h)

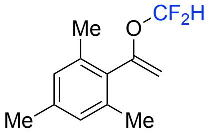

Following the **GP1**, difluoromethyl enol ether **3h** was obtained from 2',4',6'-trimethylacetophenone (**1h**, 34.0 µL, 0.200 mmol, 1.00 equiv) as a colorless liquid in 42% yield (18.0 mg, 0.0848 mmol). Eluent: *n*-pentane [R_F = 0.38].
^{1}H NMR (600 MHz, $CDCl_3$): δ = 6.89 (s, 2H), 6.36 (t, $^{2}J_F$ = 74.5, 1H), 4.98 (d, *J* = 2.6 Hz, 1H), 4.50 (d, *J* = 2.6 Hz, 1H), 2.29 (d, *J* = 6.3 Hz, 9H) ppm.
^{19}F NMR (565 MHz, $CDCl_3$): δ = –83.12 (d, *J* = 74.5 Hz, 2F) ppm.
^{13}C NMR (151 MHz, $CDCl_3$): δ = 152.8, 138.9, 136.9, 131.0, 128.3, 115.1 (t, $^{1}J_F$ = 256.7 Hz), 97.3, 21.1, 19.7 ppm.
IR (neat): $\tilde{\nu}$ = 2924, 2864, 2331, 2093, 1992, 1898, 1648, 1611, 1575, 1447, 1378, 1263, 1126, 1049, 958, 908, 852, 739 cm^{-1}.
HRMS (EI): m/z calculated for $C_{12}H_{14}OF_2$: 212.1013 $[M]^+$, found 212.1008.

2-[1-(Difluoromethoxy)vinyl]naphthalene (3i)

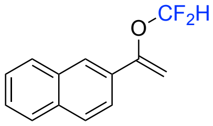

Following the **GP1**, difluoromethyl enol ether **3i** was obtained from 2-acetonaphthone (**1i**, 34.4 mg, 0.200 mmol, 1.00 equiv) in 66% yield determined by ^{1}H NMR spectroscopy.

NMR yield: 66% [δ_{DCE} = 3.73 (s, 4H) ppm; $\delta_{product}$ = 5.29 (d, *J* = 3.4 Hz, 1H), 4.86 (d, *J* = 3.5 Hz, 1H) ppm].

^{1}H NMR (600 MHz, $CDCl_3$): δ = 8.11 – 8.08 (m, 1H), 7.88 (dt, *J* = 6.2, 3.6 Hz, 1H), 7.85 – 7.82 (m, 2H), 7.66 (dd, *J* = 8.6, 1.9 Hz, 1H), 7.51 (dt, *J* = 6.2, 3.4 Hz, 2H), 6.60 (t, $^{2}J_F$ = 74.0 Hz, 1H), 5.29 (d, *J* = 3.4 Hz, 1H), 4.86 (d, *J* = 3.5 Hz, 1H) ppm.

^{19}F NMR (565 MHz, $CDCl_3$): δ = –81.28 (d, *J* = 74.3 Hz, 2F) ppm.

^{13}C NMR (151 MHz, $CDCl_3$): δ = 154.3, 133.7, 133.0, 130.7, 128.7, 128.3, 127.6, 126.9, 126.6, 124.9, 122.7, 115.9 (t, $^{1}J_F$ = 256.7 Hz), 93.7 ppm.

IR (neat): $\tilde{\nu}$ = 3438, 3059, 2921, 2852, 2396, 2285, 1923, 1806, 1678, 1631, 1575, 1507, 1466, 1435, 1382, 1286, 1235, 1197, 1127, 1051, 966, 900, 856, 817, 750, 718, 664, 575, 533, 475 cm^{-1}.

HRMS (EI): m/z calculated for $C_{13}H_{10}OF_2$: 220.0700 [M]$^+$, found 220.0694.

[3-(Difluoromethoxy)but-3-en-1-yl]benzene (3j)

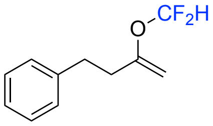

Following the **GP1**, difluoromethyl enol ether **3j** was obtained from benzylacetone (**1j**, 30.3 μL, 0.200 mmol, 1.00 equiv) in 42% yield determined by ^{1}H NMR spectroscopy.

NMR yield: 42% [δ_{DCE} = 3.73 (s, 4H) ppm; $\delta_{product}$ = 4.33 (dt, *J* = 3.0, 1.0 Hz, 1H), 4.29 (dt, *J* = 3.0, 1.0 Hz, 1H) ppm].

^{1}H NMR (600 MHz, $CDCl_3$): δ = 7.31 – 7.28 (m, 2H), 7.20 – 7.18 (m, 3H), 6.38 (t, $^{2}J_F$ = 74.3 Hz, 1H), 4.33 (dt, *J* = 3.0, 1.0 Hz, 1H), 4.29 (dt, *J* = 3.0, 1.0 Hz, 1H), 2.84 (t, *J* = 7.6 Hz, 2H), 2.47 (t, *J* = 8.0 Hz, 2H) ppm.

^{19}F NMR (564 MHz, $CDCl_3$): δ = –82.56 (d, *J* = 74.1 Hz, 2F) ppm.

^{13}C NMR (151 MHz, $CDCl_3$): δ = 156.4, 140.6, 128.4, 128.4, 126.1, 115.2 (t, $^{1}J_F$ = 256.7 Hz), 91.5, 36.0, 32.8 ppm.

IR (neat): $\tilde{\nu}$ = 3064, 3028, 2927, 2858, 2326, 2085, 1998, 1653, 1604, 1496, 1453, 1380, 1288, 1254, 1126, 1046, 972, 916, 845, 744, 698, 658 cm^{-1}.

HRMS (EI): m/z calculated for $C_{11}H_{12}OF_2$: 198.0856 [M]$^+$, found 198.0851.

2,4-Dichloro-1-[3-(difluoromethoxy)buta-1,3-dien-1-yl]benzene (3k)

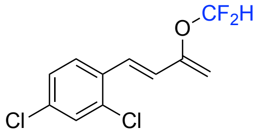

Following the **GP1**, difluoromethyl enol ether **3k** was obtained from 2,4-dichlorobenzylideneacetone (**1k**, 44.3 mg, 0.200 mmol, 1.00 equiv) in 51% yield determined by ^{1}H NMR spectroscopy, and in 13% yield (6.8 mg, 0.0257 mmol) after column chromatography. Eluent: *n*-pentane [R_F = 0.39].

NMR yield: 51% [δ_{DCE} = 3.73 (s, 4H) ppm; $\delta_{product}$ = 6.64 – 6.35 (m, 2H), 4.79 (d, *J* = 6.9 Hz, 2H) ppm].

^{1}H NMR (600 MHz, $CDCl_3$): δ = 7.47 (d, *J* = 8.5 Hz, 1H), 7.40 (s, 1H), 7.28 – 7.25 (m, 1H), 7.22 (d, *J* = 8.4 Hz, 1H), 6.50 (t, *J* = 75.0 Hz, 1H), 6.49 (d, *J* = 18.0 Hz, 1H), 4.79 (d, *J* = 6.9 Hz, 2H) ppm.

^{19}F NMR (565 MHz, $CDCl_3$): δ = –81.60 (d, *J* = 73.7 Hz, 2F) ppm.

^{13}C NMR (151 MHz, $CDCl_3$): δ = 152.6, 134.4, 134.4, 132.8, 129.7, 127.5, 127.3, 126.0, 125.1, 115.5 (t, $^{1}J_F$ = 256.7 Hz), 98.8 ppm.

IR and **HRMS** measurements were not successful.

3-(Difluoromethoxy)-1-phenylbut-2-en-1-one (3l)

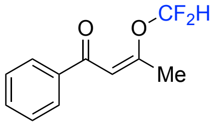

Following the **GP2**, difluoromethyl enol ether **3l** was obtained from benzoylacetone (**1l**, 33.1 mg, 0.200 mmol, 1.00 equiv) in 25% yield (10.5 mg, 0.0495 mmol).

^{1}H NMR (600 MHz, $CDCl_3$): δ = 7.62 – 7.59 (m, 2H), 7.51 – 7.48 (m, 1H), 7.47 – 7.44 (m, 2H), 6.53 (t, $^{2}J_F$ = 74.2 Hz, 1H), 6.11 (s, 1H), 2.48 (s, 3H) ppm.

^{19}F NMR (564 MHz, $CDCl_3$): δ = –82.48 (d, *J* = 74.1 Hz, 2F) ppm.

^{13}C NMR (151 MHz, $CDCl_3$): δ = 196.7, 132.9, 131.4, 129.1, 128.5, 127.3, 116.1 (t, $^{1}J_F$ = 256.7 Hz), 115.9, 31.5 ppm.

IR (neat): $\tilde{\nu}$ = 3499, 2926, 2855, 2328, 2160, 2111, 1993, 1898, 1692, 1665, 1600, 1493, 1447, 1424, 1359, 1258, 1182, 1105, 1054, 894, 835, 772, 695 cm^{-1}.

HRMS (ESI): m/z calculated for $C_{11}H_{10}O_2F_2Na$: 235.0547 $[M+Na]^+$, found 235.0541.

The NMR spectra are in accordance with the literature [14].

4-(Difluoromethoxy)-*2H*-chromene (3m)

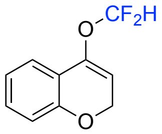

Following the **GP1**, difluoromethyl enol ether **3m** was obtained from 4-chromanone (**1m**, 30.5 mg, 0.200 mmol, 1.00 equiv) in 50% yield determined by ^{1}H NMR spectroscopy.

NMR yield: 50% [δ_{DCE} = 3.73 (s, 4H) ppm; $\delta_{product}$ = 5.23 (t, *J* = 3.8 Hz, 1H) ppm].

^{1}H NMR (600 MHz, $CDCl_3$): δ = 7.35 (dd, *J* = 7.7, 1.7 Hz, 1H), 7.21 (td, *J* = 7.8, 1.7 Hz, 1H), 6.94 (td, *J* = 7.5, 1.2 Hz, 1H), 6.82 (dd, *J* = 8.1, 1.1 Hz, 1H), 6.51 (t, $^{2}J_F$ = 73.8 Hz, 1H), 5.23 (t, *J* = 3.8 Hz, 1H), 4.88 (d, *J* = 3.9 Hz, 2H) ppm.
^{19}F NMR (564 MHz, $CDCl_3$): δ = –81.77 (d, *J* = 73.7 Hz, 2F) ppm.
^{13}C NMR (151 MHz, $CDCl_3$): δ = 161.9, 136.7, 136.1, 130.7, 130.0, 127.1, 121.4, 117.9 (t, $^{1}J_F$ = 6.0 Hz), 100.3, 67.0 ppm.

1-(Difluoromethoxy)-7-phenylcyclohept-1-ene (3n)

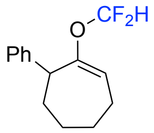

Following the **GP1**, difluoromethyl enol ether **3n** was obtained from 2-phenylcycloheptanone (**1n**, 37.7 mg, 0.200 mmol, 1.00 equiv) in 44% yield determined by ^{1}H NMR spectroscopy, and in 18% yield (8.60 mg, 0.0361 mmol) after purification.
^{1}H NMR (600 MHz, $CDCl_3$): δ = 7.34 – 7.29 (m, 2H), 7.25 – 7.21 (m, 3H), 6.21 (dd, $^{2}J_F$ = 76.6, 73.5 Hz, 1H), 5.61 (t, *J* = 6.5 Hz, 1H), 3.74 (t, *J* = 5.6 Hz, 1H), 2.29 – 2.14 (m, 2H), 2.05 – 1.99 (m, 2H), 1.76 – 1.65 (m, 2H), 1.65 – 1.58 (m, 1H), 1.49 (tt, *J* = 12.6, 6.1 Hz, 1H) ppm.
^{19}F NMR (565 MHz, $CDCl_3$): δ = –80.44 (d, *J* = 73.5 Hz, 2F) ppm.
^{13}C NMR (151 MHz, $CDCl_3$): δ = 153.4, 140.4, 128.4, 127.7, 126.4, 114.0 (t, $^{1}J_F$ = 191.9 Hz), 58.8, 43.4, 32.0, 26.5, 24.0, 23.4 ppm.
IR (neat): $\tilde{\nu}$ = 3374, 3028, 2927, 2857, 2325, 2157, 2084, 1992, 1723, 1670, 1601, 1493, 1450, 1373, 1258, 1162, 1023, 867, 795, 699 cm^{-1}.
HRMS (EI): m/z calculated for $C_{14}H_{16}OF_2$: 238.1169 $[M]^+$, found 238.1164.

6. Unsuccessful difluoromethylations

6.1. Use of aryl ketones

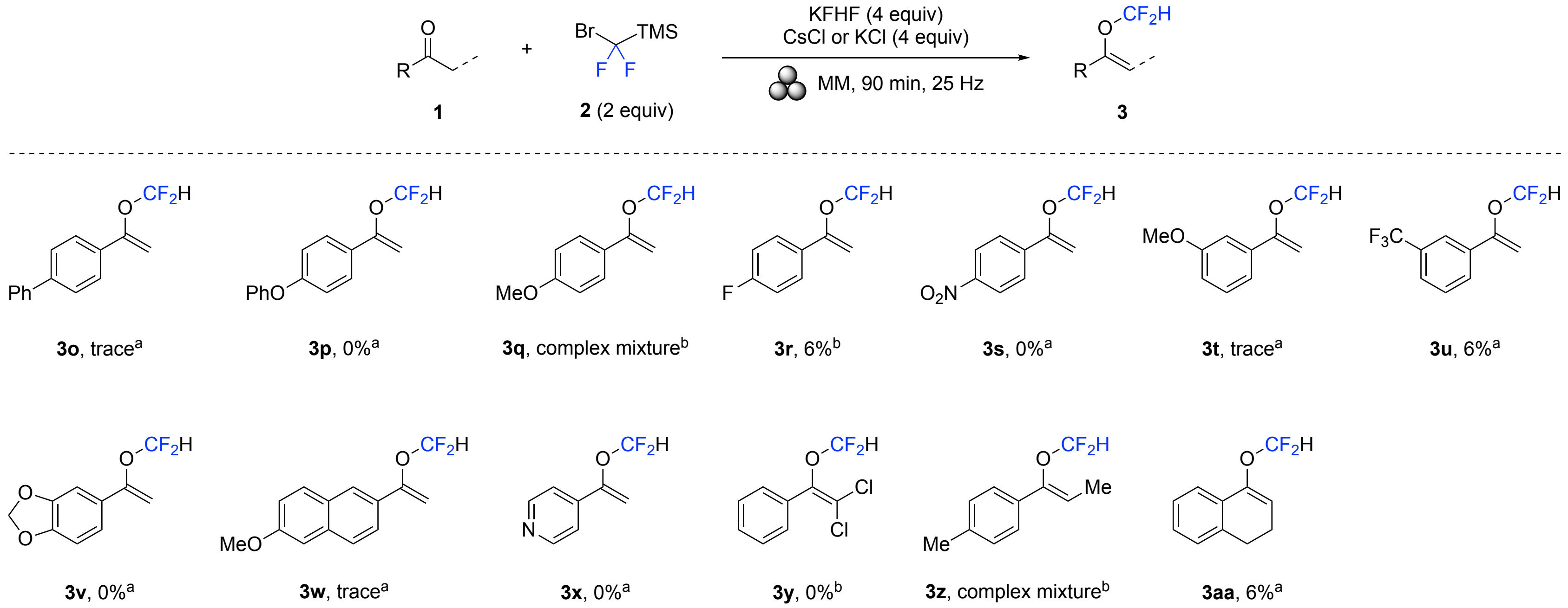

Scheme S1: Products that could only be obtained in traces of not at all following the general procedure for the difluoromethylation of ketones. The yields were determined by ^{1}H NMR spectroscopy using 1,2-dichloroethane as the internal standard. [a]With CsCl. [b]With KCl.

6.2. Use of 1-aryl prop-2-en-1-ones

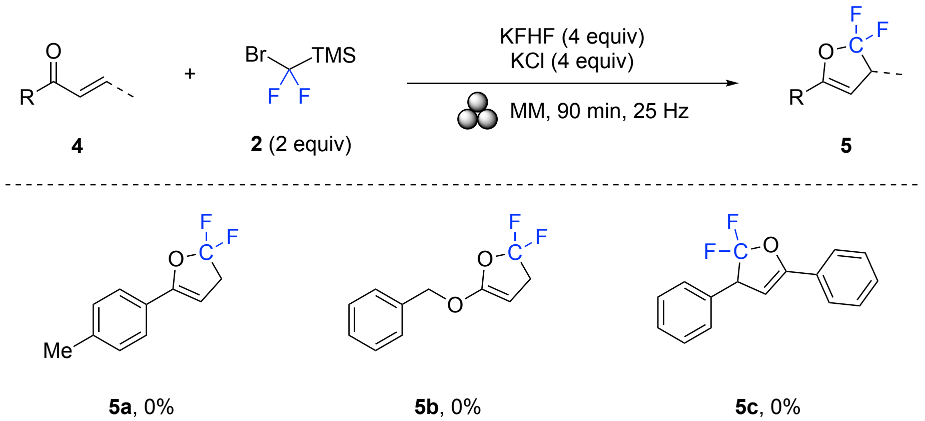

Scheme S2: 1-Aryl 2,2-difluoro-2,3-dihydrofurans that could not be obtained from the corresponding prop-2-en-1-ones following the general procedure for the difluoromethylation of ketones.

7. Mechanistic investigations

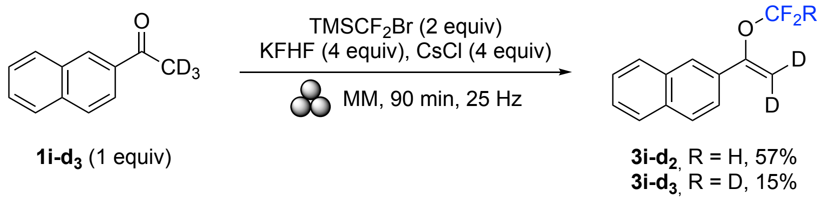

Scheme S3: Difluoromethylation of deuterated 2-acetonaphthone (**1j-d₃**). The yield was determined by ^{1}H NMR spectroscopy using 1,2-dichloroethane (1.00 equiv) as the internal standard.

^{1}H NMR of the crude reaction mixture

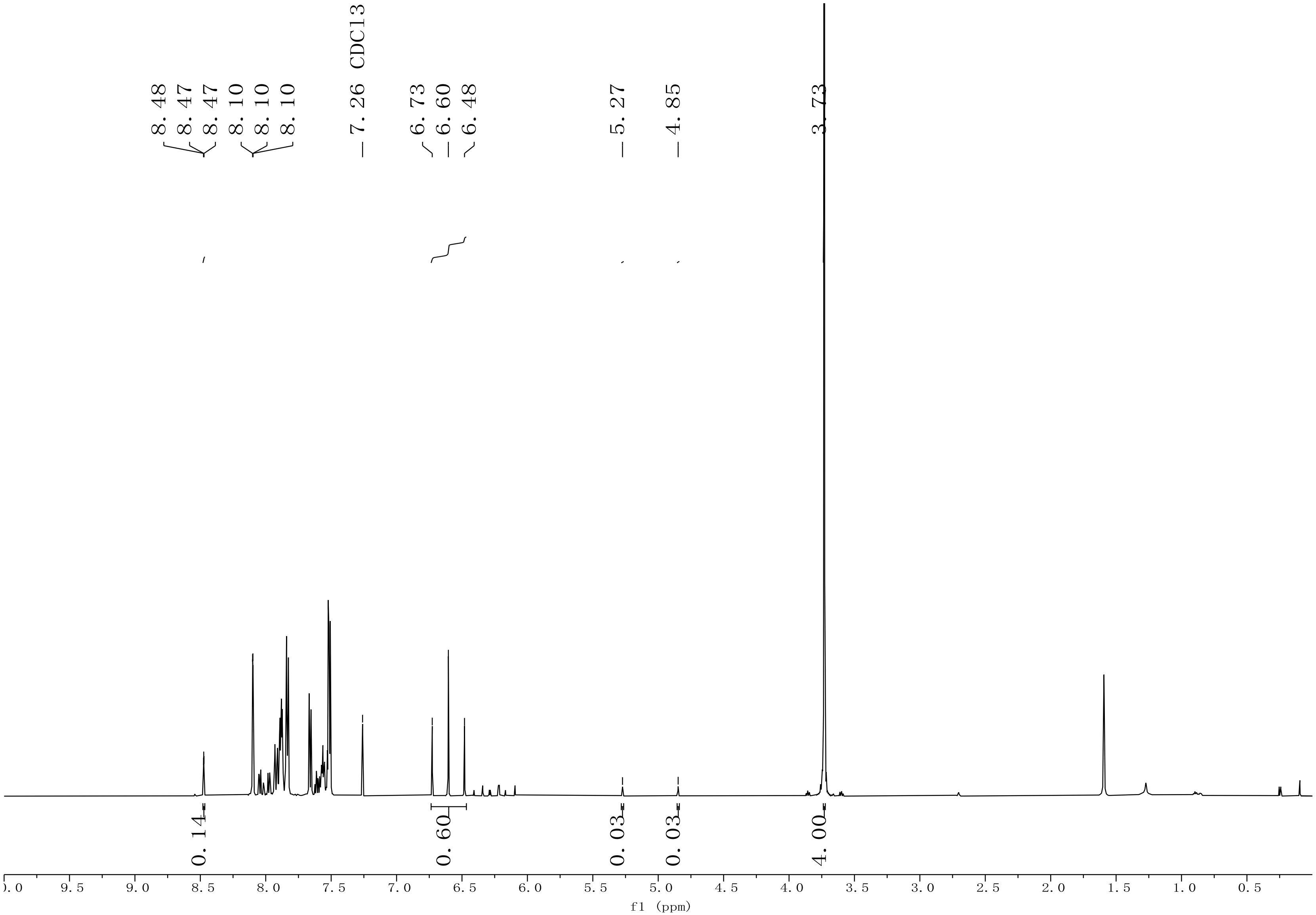

^{19}F NMR of the crude reaction mixture

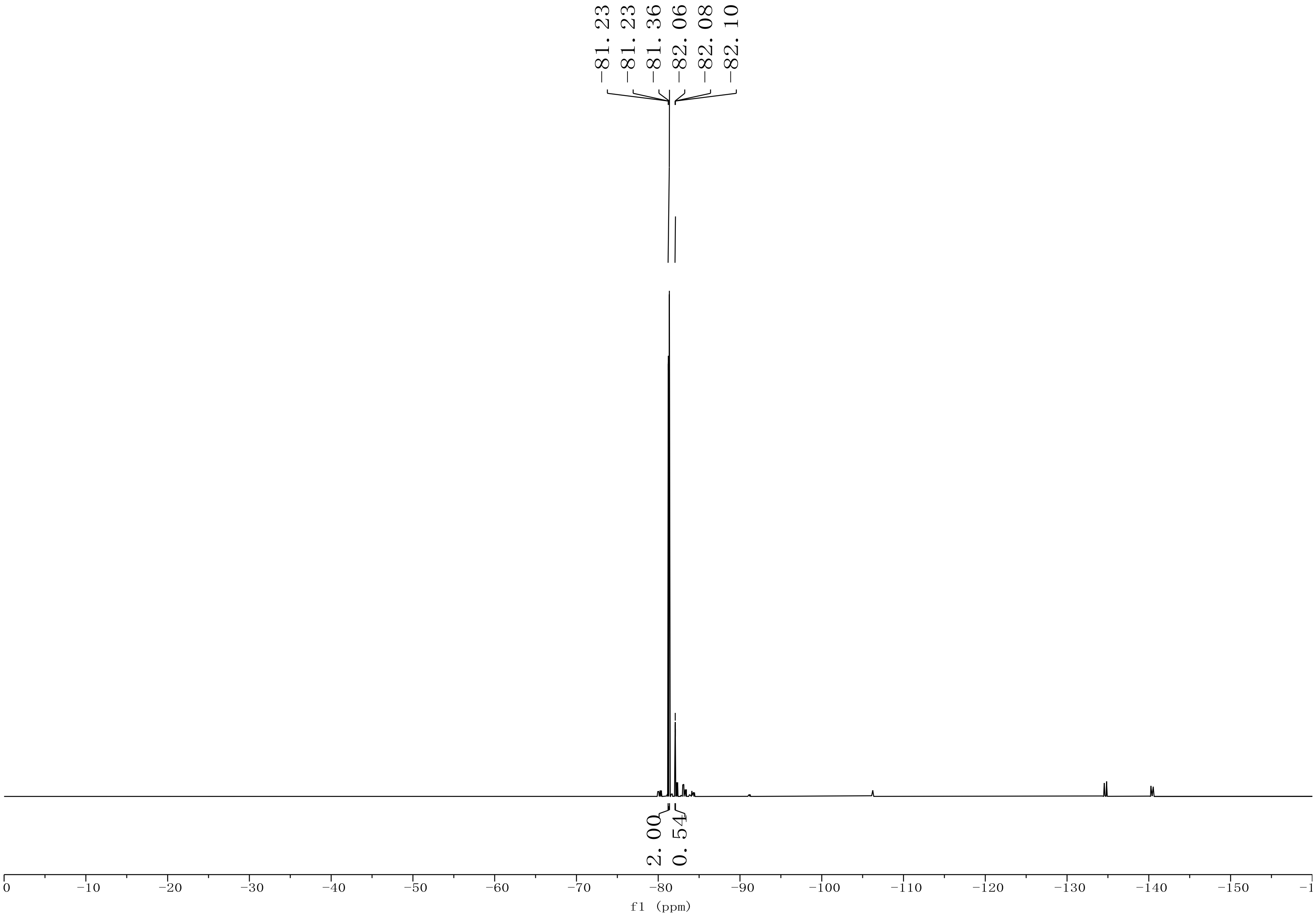

$^{13}C\{^{1}H\}$ NMR of the crude reaction mixture

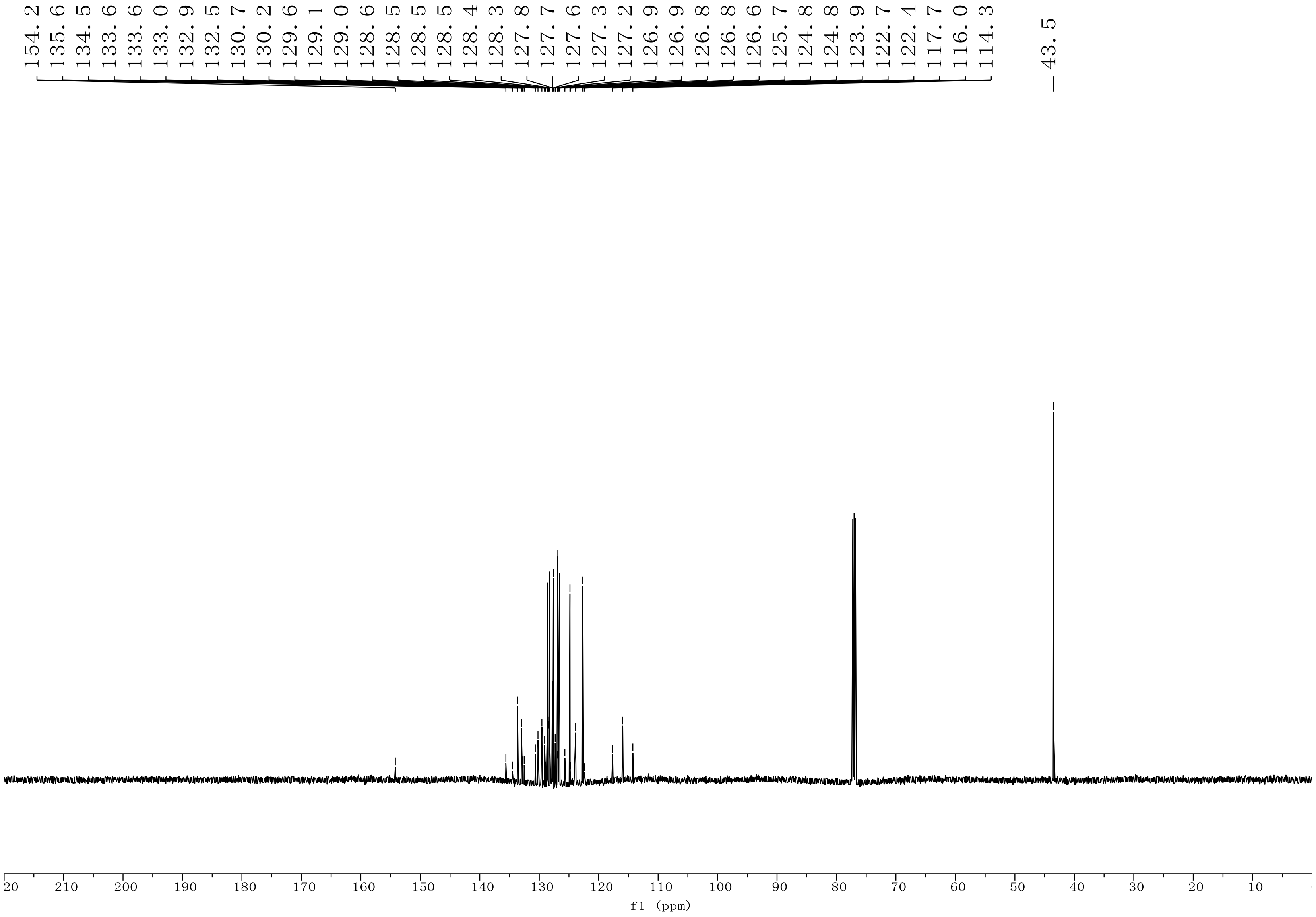

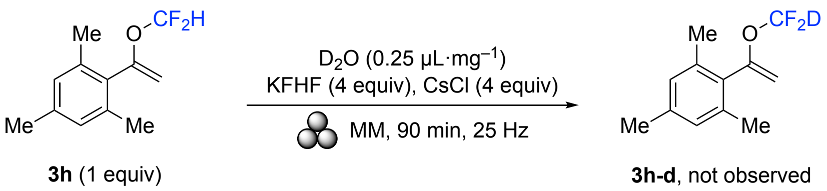

Scheme S4: Hydrogen–deuterium exchange of difluoromethyl enol ether **2i**.

^{1}H NMR of the crude reaction mixture

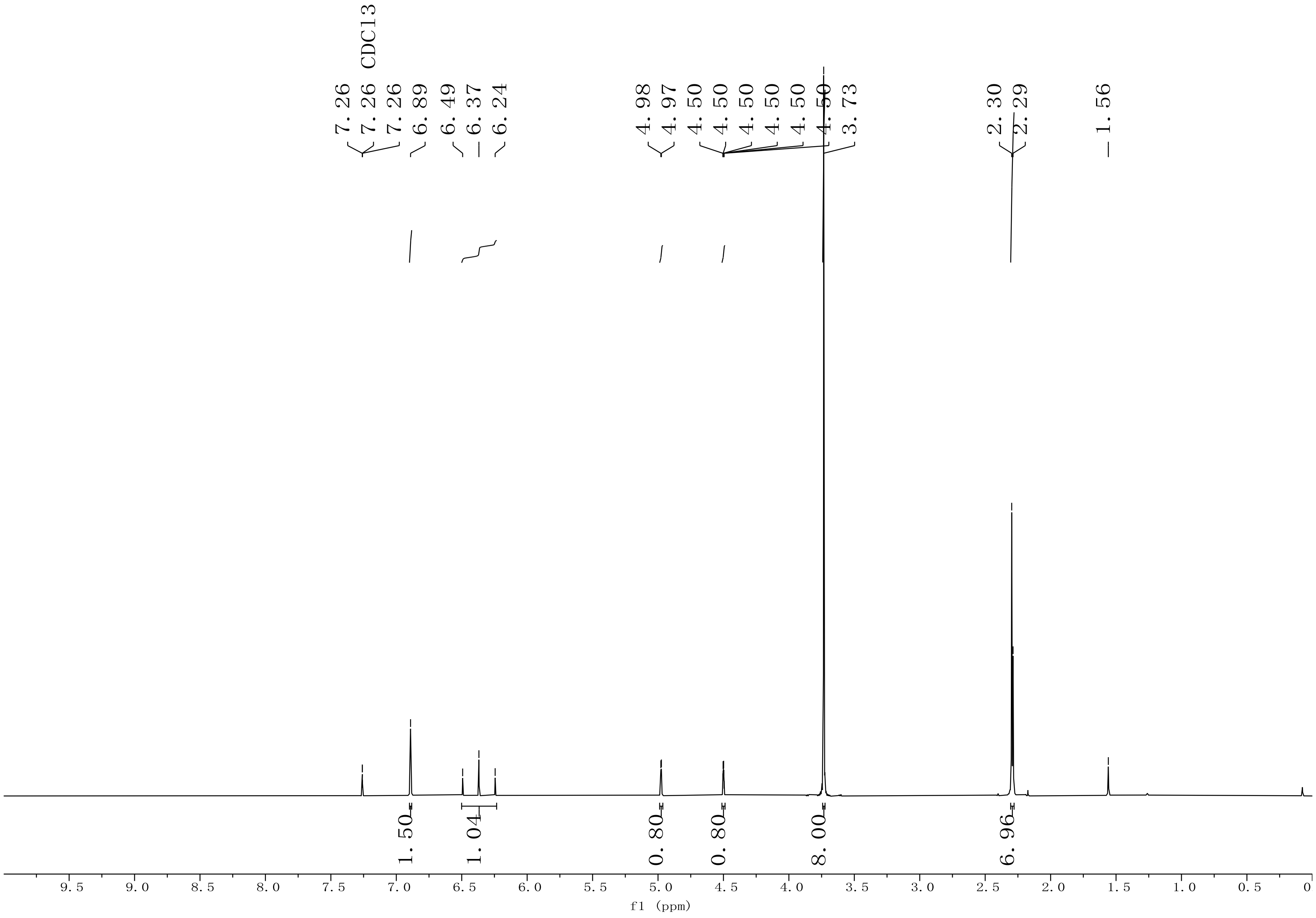

^{19}F NMR of the crude reaction mixture

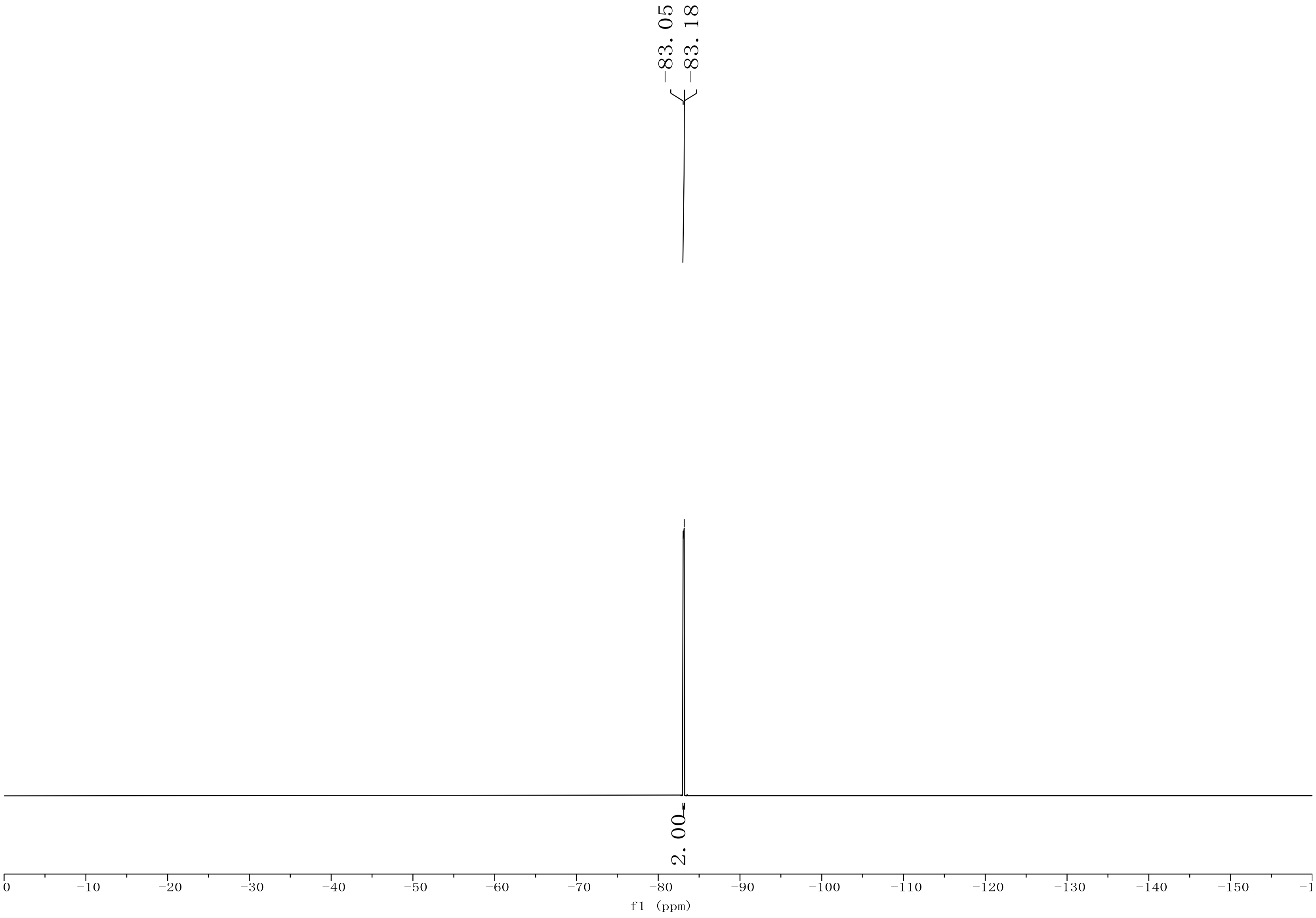

$^{13}C\{^1H\}$ NMR of the crude reaction mixture

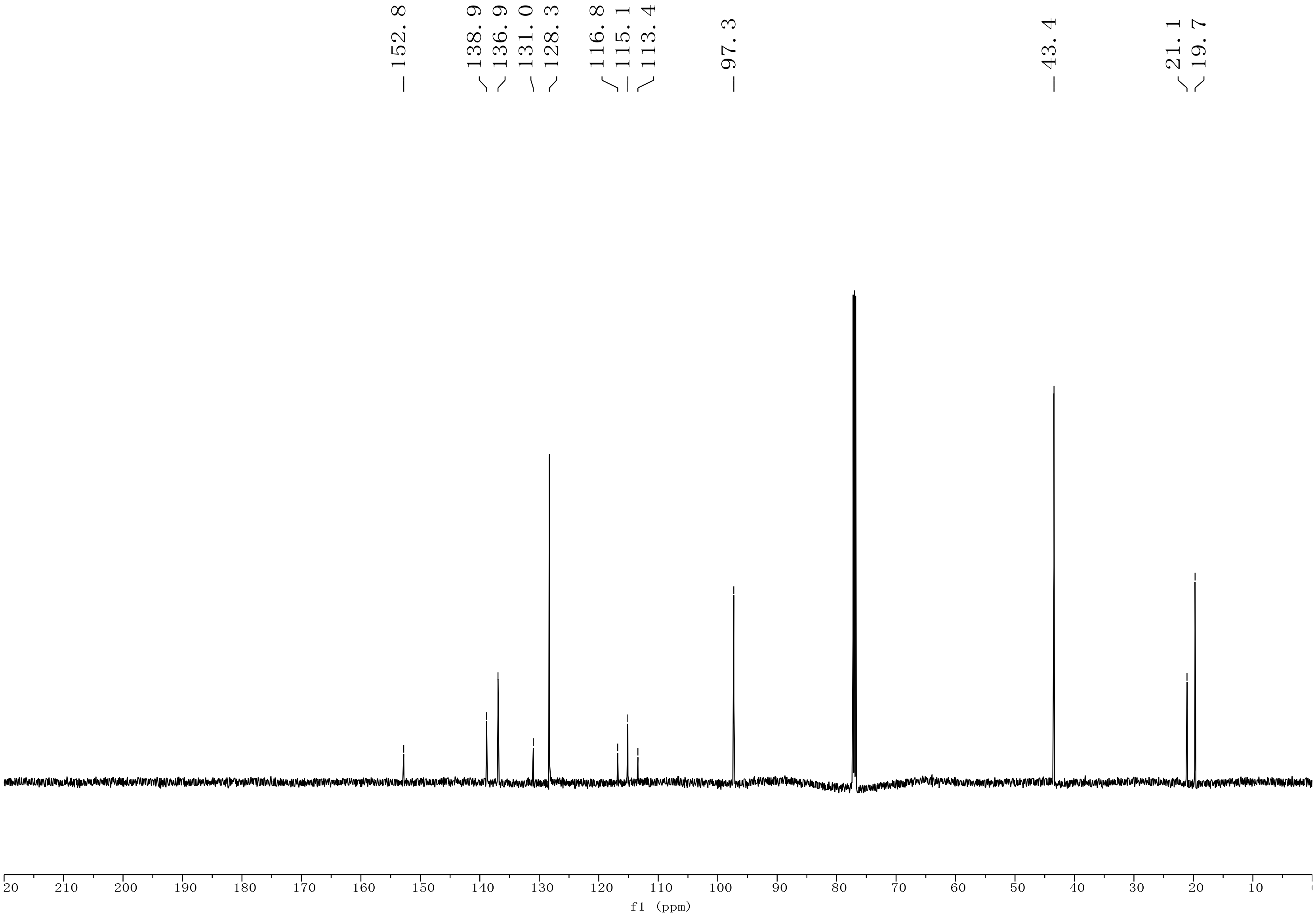

8. References

1. Zhang, Q.; Jiang, P.; Wang, K.; Song, G.; Zhu, H. *Dyes Pigments* **2011**, *91*, 89–97.
2. Sambiagio, C.; Munday, R. H.; Marsden, S. P.; Blacker, A. J.; McGowan, P. C. *Chem. Eur. J.* **2014**, *20*, 17606–17615.
3. Cui, C.; Wang, X.; Weiss, R. G. *J. Org. Chem.* **1996**, *61*, 1962–1974.
4. Bugarin, A.; Jones, K. D.; Connell, B. T. *Chem. Commun.* **2010**, *46*, 1715–1717.
5. Darshana, D.; Sureram, S.; Mahidol, C.; Ruchirawat, S.; Kittakoop, P. *Org. Biomol. Chem.* **2021**, *19*, 7390–7402.
6. van Bonn, P.; Ke, J.; Weike, C.; Ward, J. S.; Rissanen, K.; Bolm, C. *CCS Chem.* **2023**, *5*, 1737–1744.
7. Zafrani, Y.; Sod-Moriah, G.; Segall, Y. *Tetrahedron* **2009**, *65*, 5278–5283.
8. Liu, G.-K.; Li, X.; Qin, W.-B.; Peng, X.-S.; Wong, H. N. C.; Zhang, L.; Zhang, X. *Chem. Commun.* **2019**, *55*, 7446–7449.
9. Polley, A.; Bairy, G.; Das, P.; Jana, R. *Adv. Synth. Catal.* **2018**, *360*, 4161–4167.
10. Tian, F.; Kruger, V.; Bautista, O.; Duan, J.-X.; Li, A.-R.; Dolbier, W. R.; Chen, Q.-Y. *Org. Lett.* **2000**, *2*, 563–564.
11. Mehta, V. P.; Greaney, M. F. *Org. Lett.* **2013**, *15*, 5036–5039.
12. Xie, Q.; Ni, C.; Zhang, R.; Li, L.; Rong, J.; Hu, J. *Angew. Chem., Int. Ed.* **2017**, *56*, 3206–3210.
13. Fuchibe, K.; Koseki, Y.; Sasagawa, H.; Ichikawa, J. *Chem. Lett.* **2011**, *40*, 1189–1191.
14. Liu, G.-K.; Li, X.; Qin, W.-B.; Lin, W.-F.; Lin, L.-T.; Chen, J.-Y.; Liu, J.-J. *Chin. Chem. Lett.* **2019**, *30*, 1515–1518.

9. NMR spectra

^{1}H NMR of the crude reaction mixture of 1-[1-(difluoromethoxy)vinyl]-4-methylbenzene (3a)

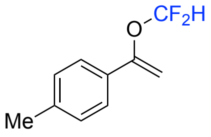

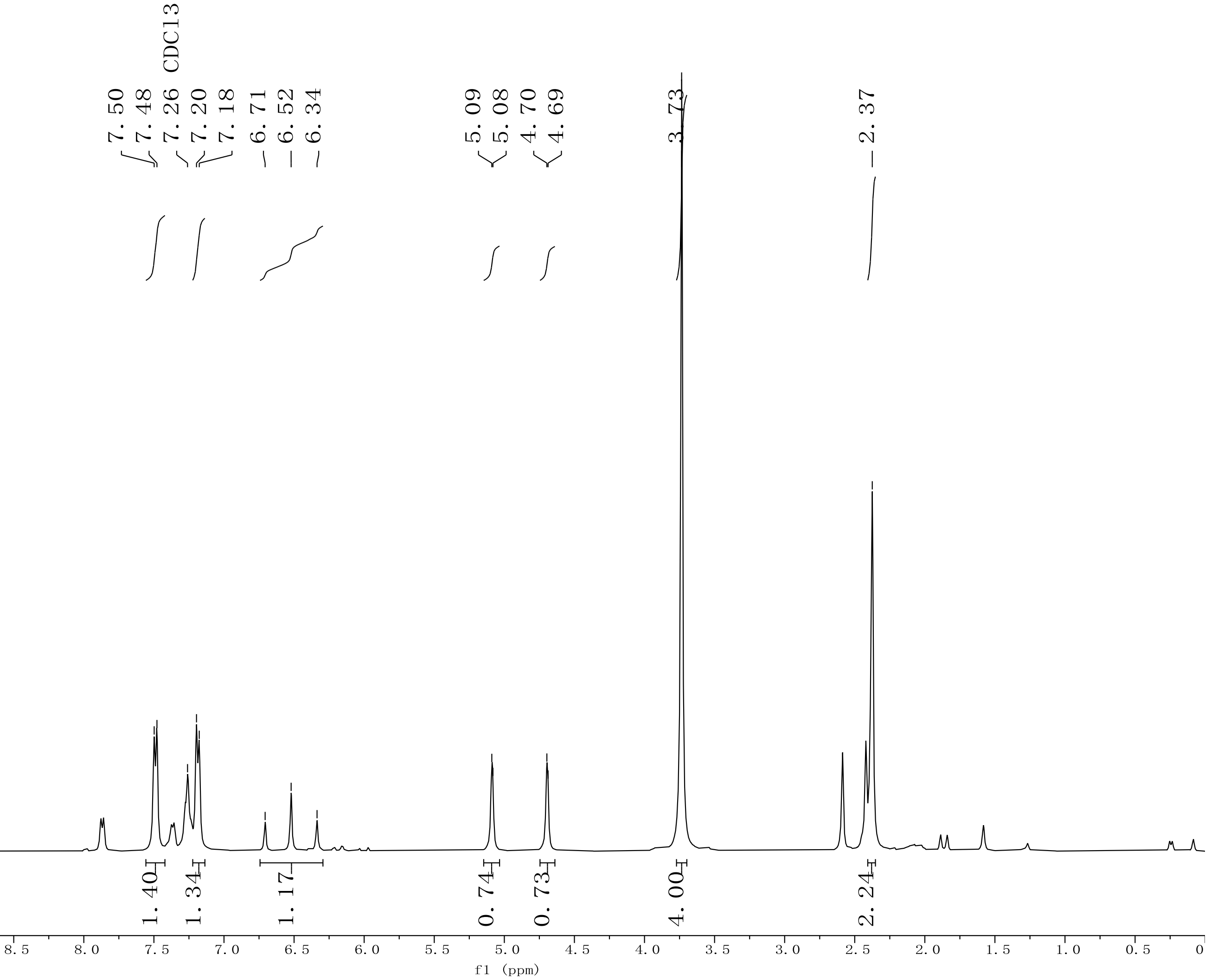

^{19}F NMR of the crude reaction mixture of 1-[1-(difluoromethoxy)vinyl]-4-methylbenzene (3a)

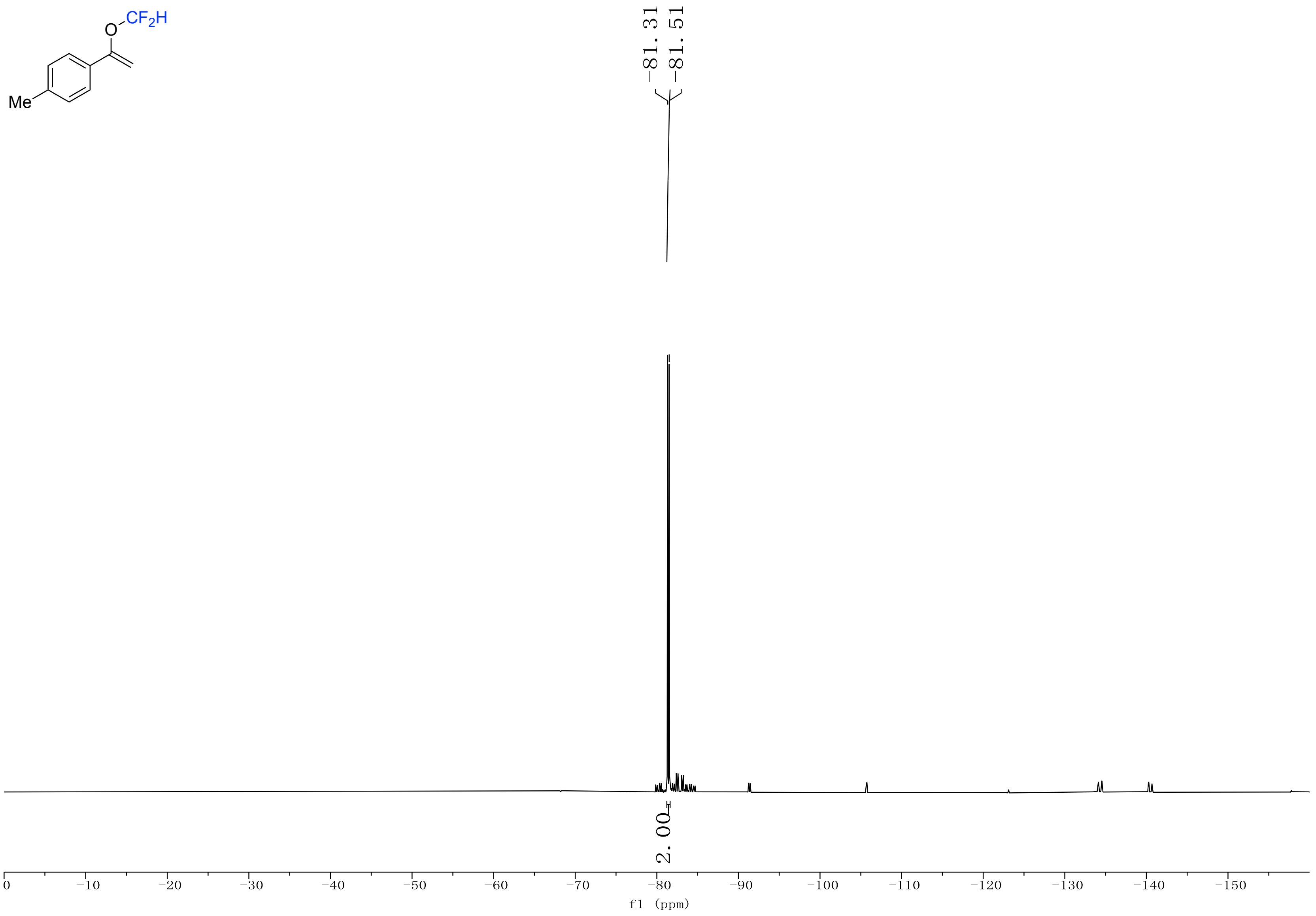

$^{13}C\{^1H\}$ NMR of the crude reaction mixture of 1-[1-(difluoromethoxy)vinyl]-4-methylbenzene (3a)

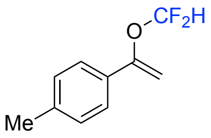

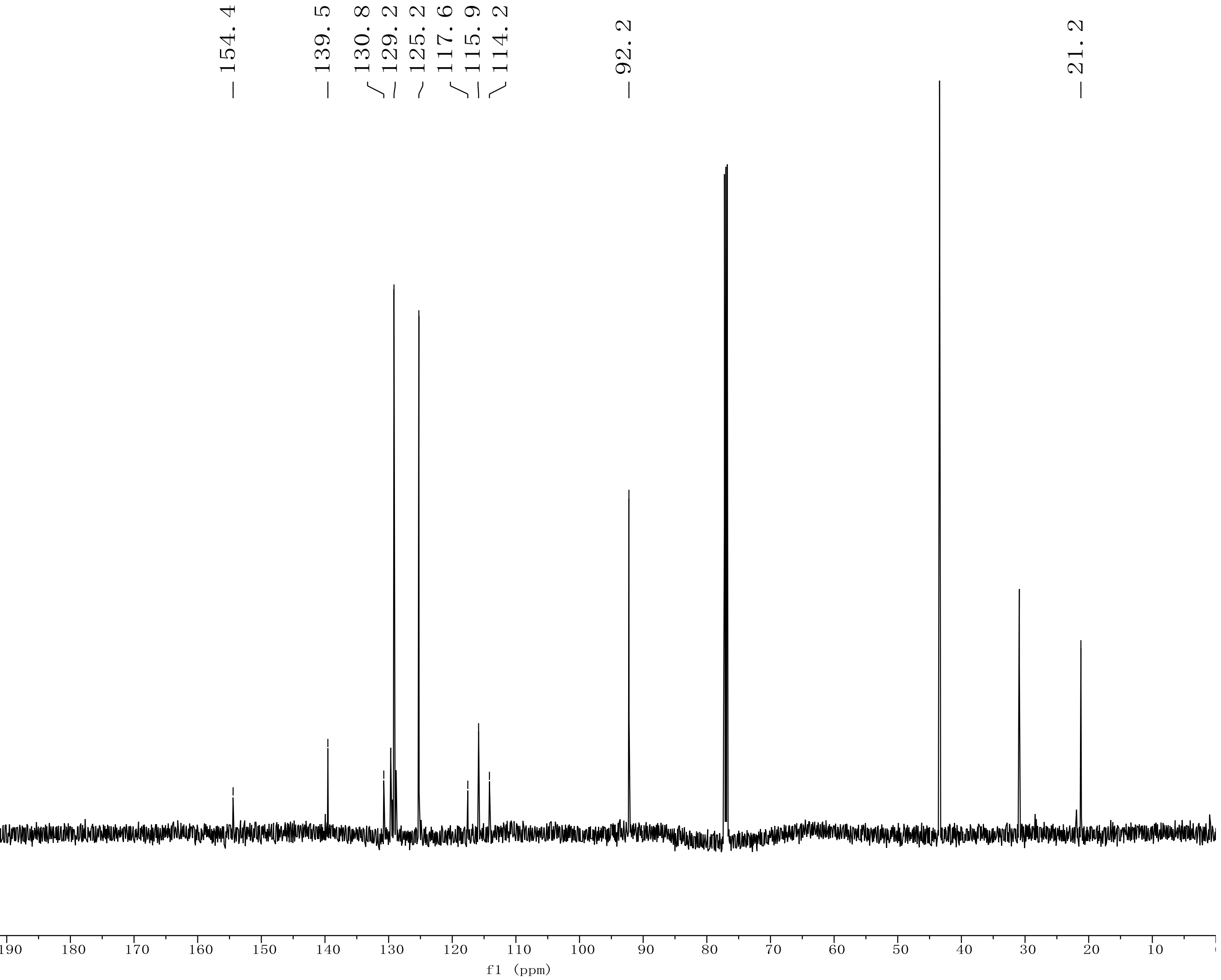

^{1}H NMR of the crude reaction mixture of [1-(difluoromethoxy)vinyl]benzene (3b)

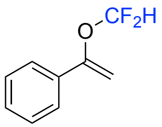

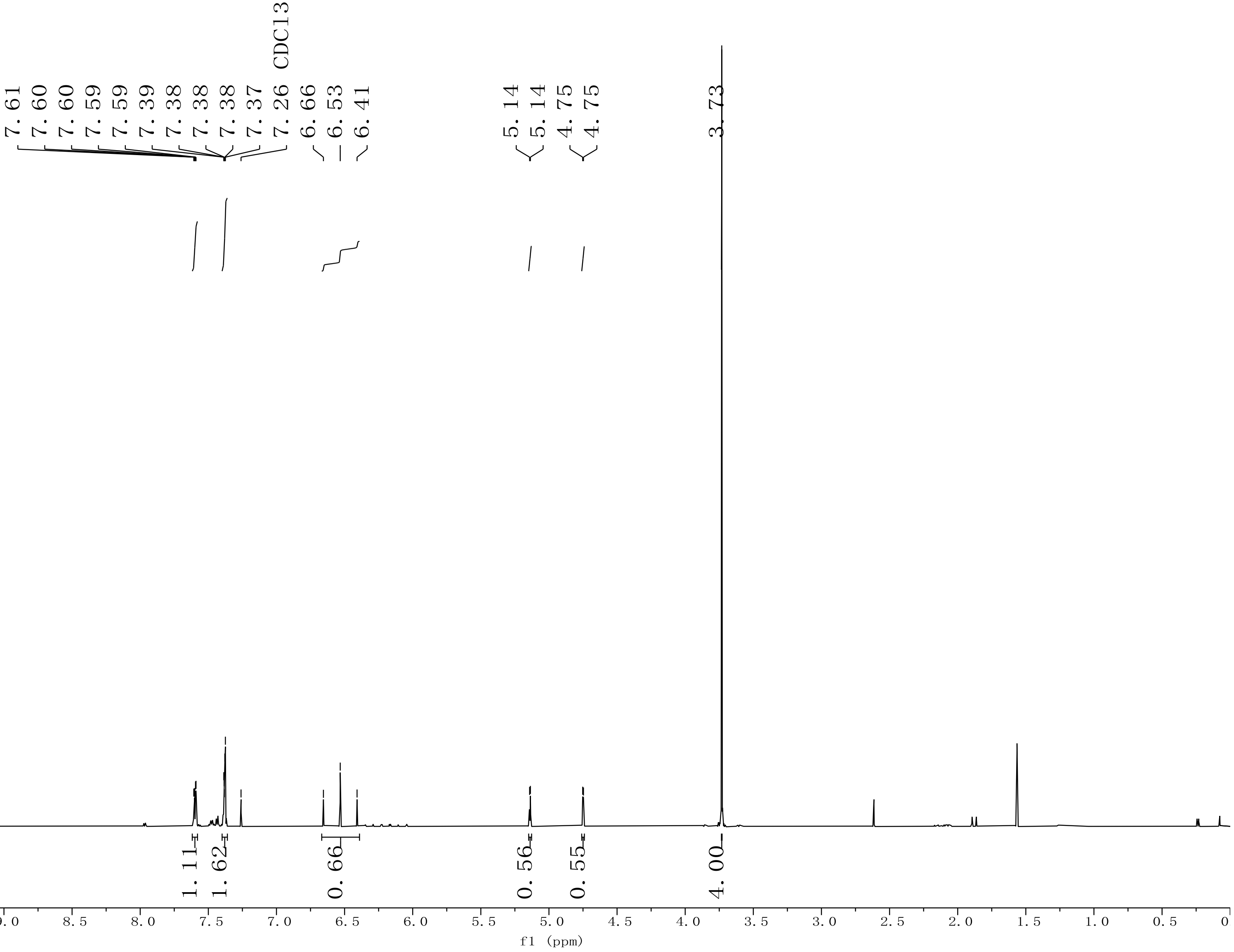

^{19}F NMR of the crude reaction mixture of [1-(difluoromethoxy)vinyl]benzene (3b)

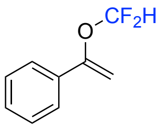

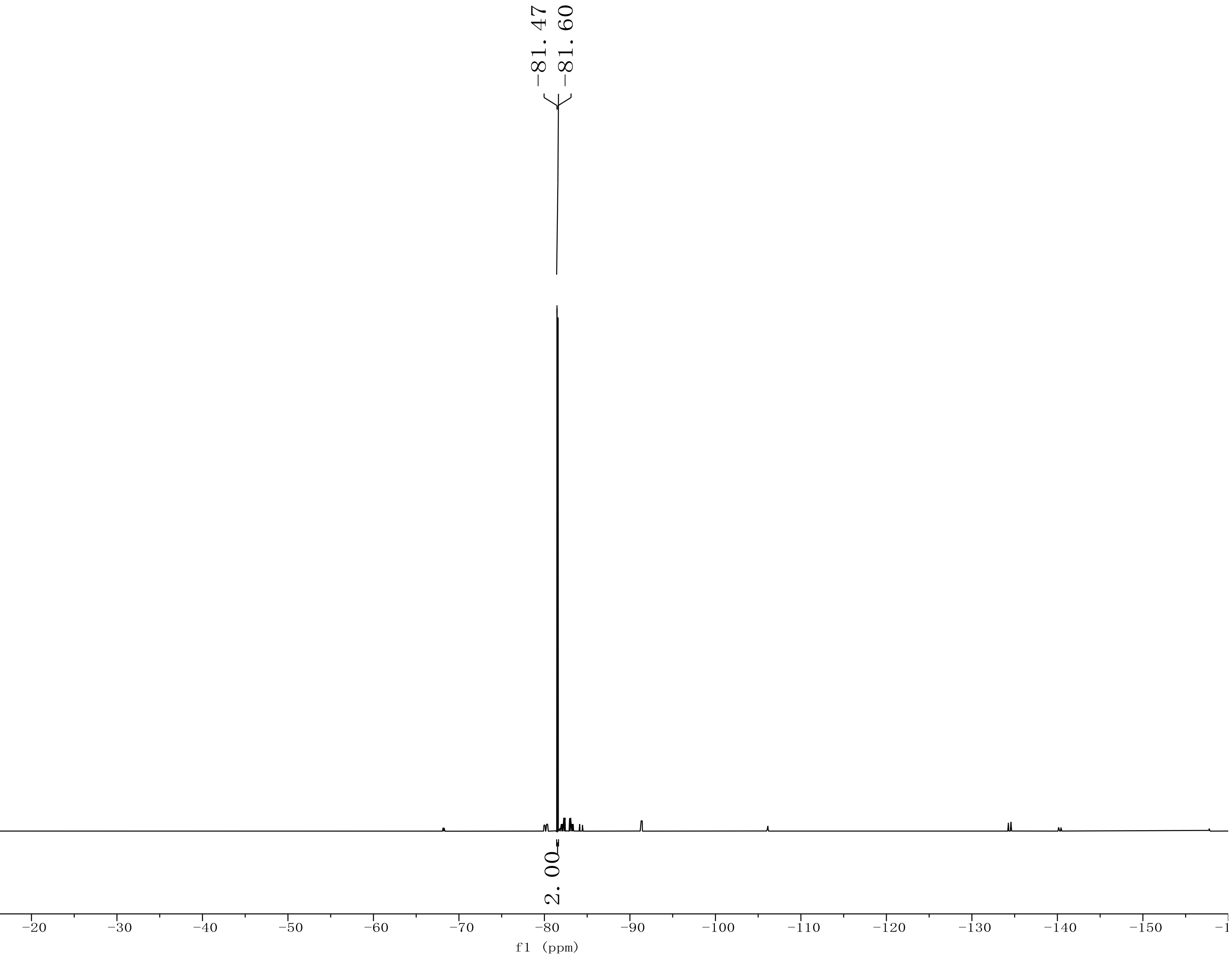

$^{13}C\{^{1}H\}$ NMR of the crude reaction mixture of [1-(difluoromethoxy)vinyl]benzene (3b)

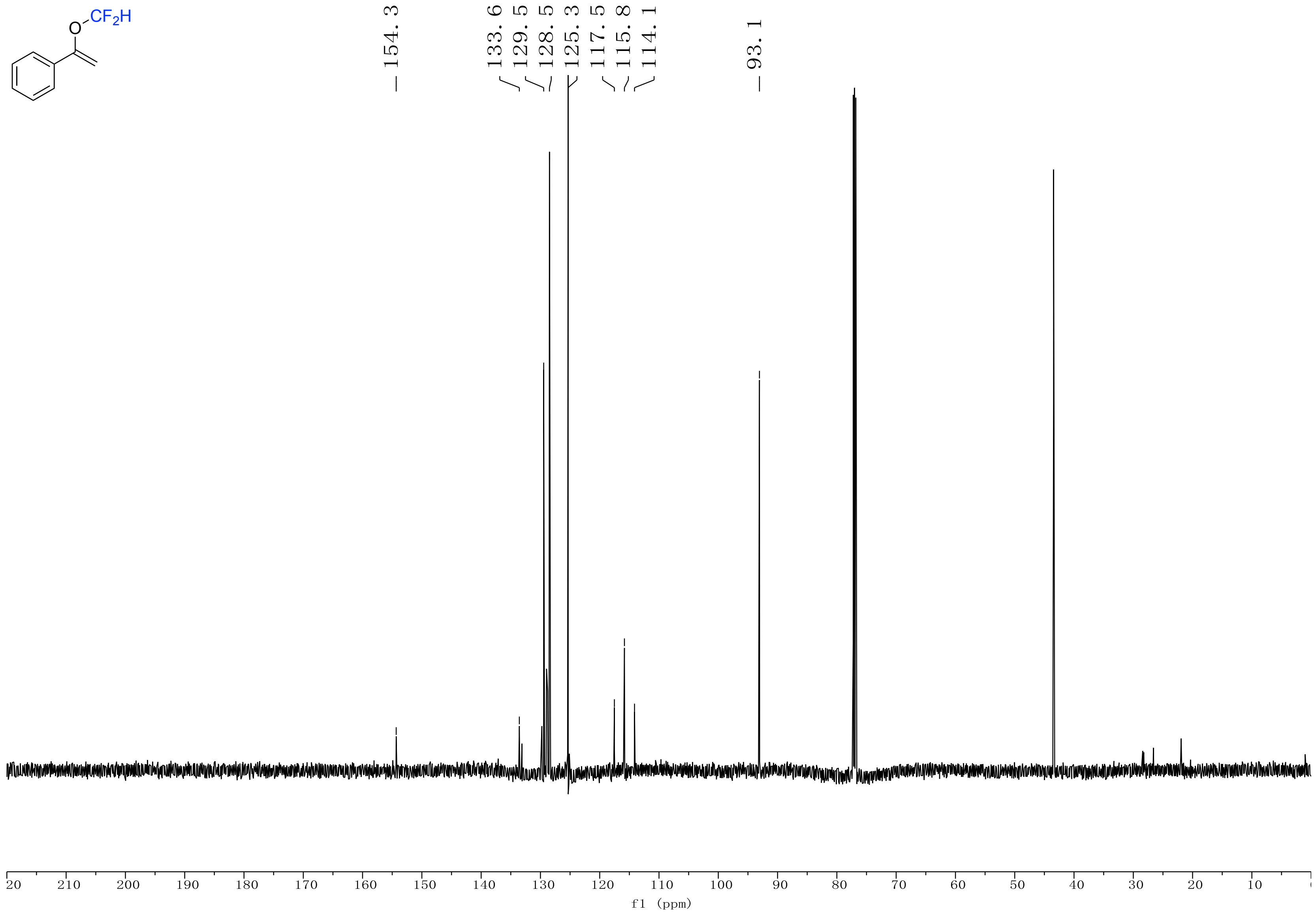

^{1}H NMR of 1-chloro-4-[1-(difluoromethoxy)vinyl]benzene (3c)

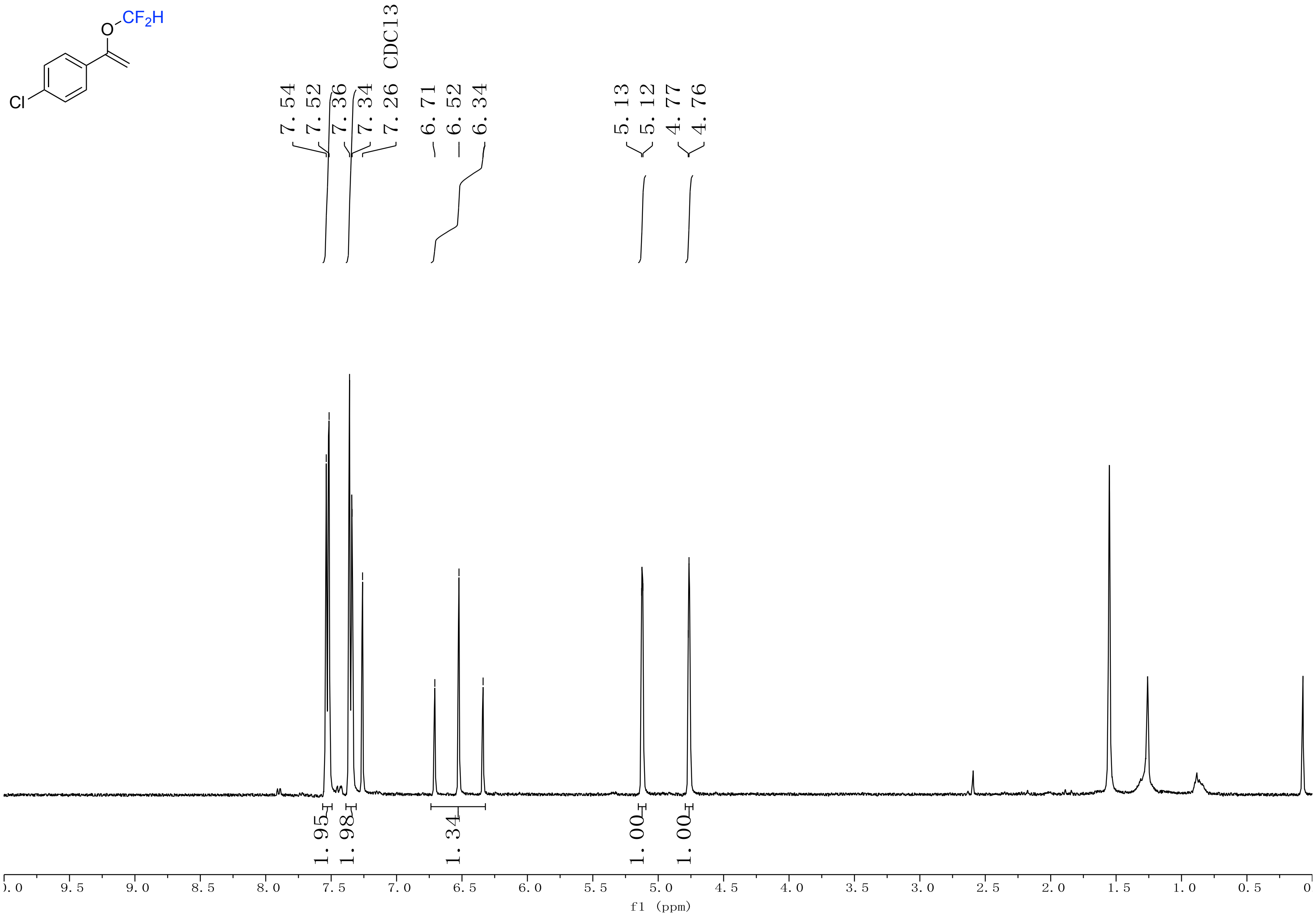

^{19}F NMR of 1-chloro-4-[1-(difluoromethoxy)vinyl]benzene (3c)

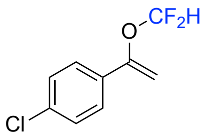

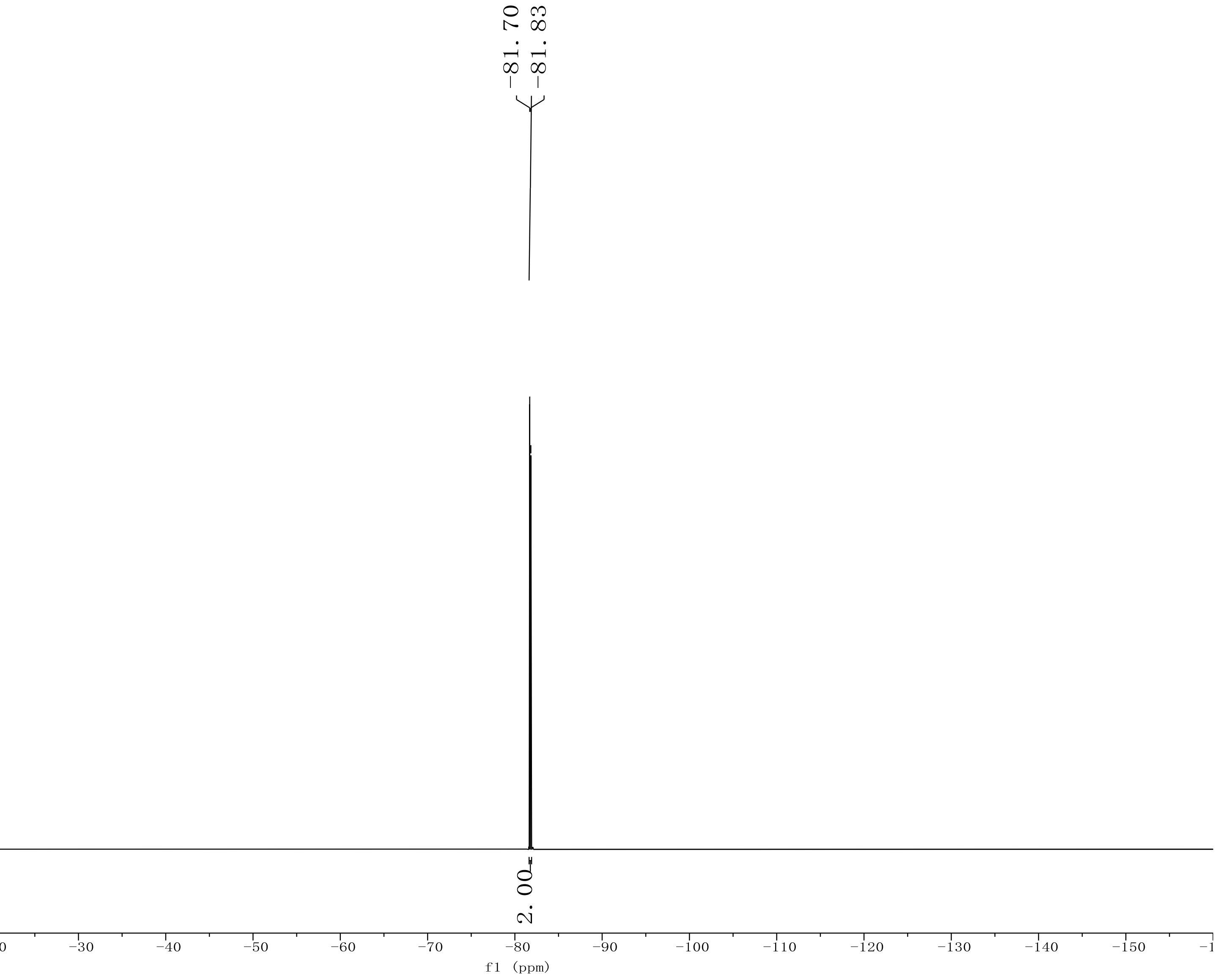

$^{13}C\{^{1}H\}$ NMR of 1-chloro-4-[1-(difluoromethoxy)vinyl]benzene (3c)

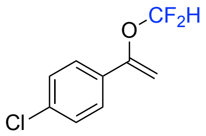

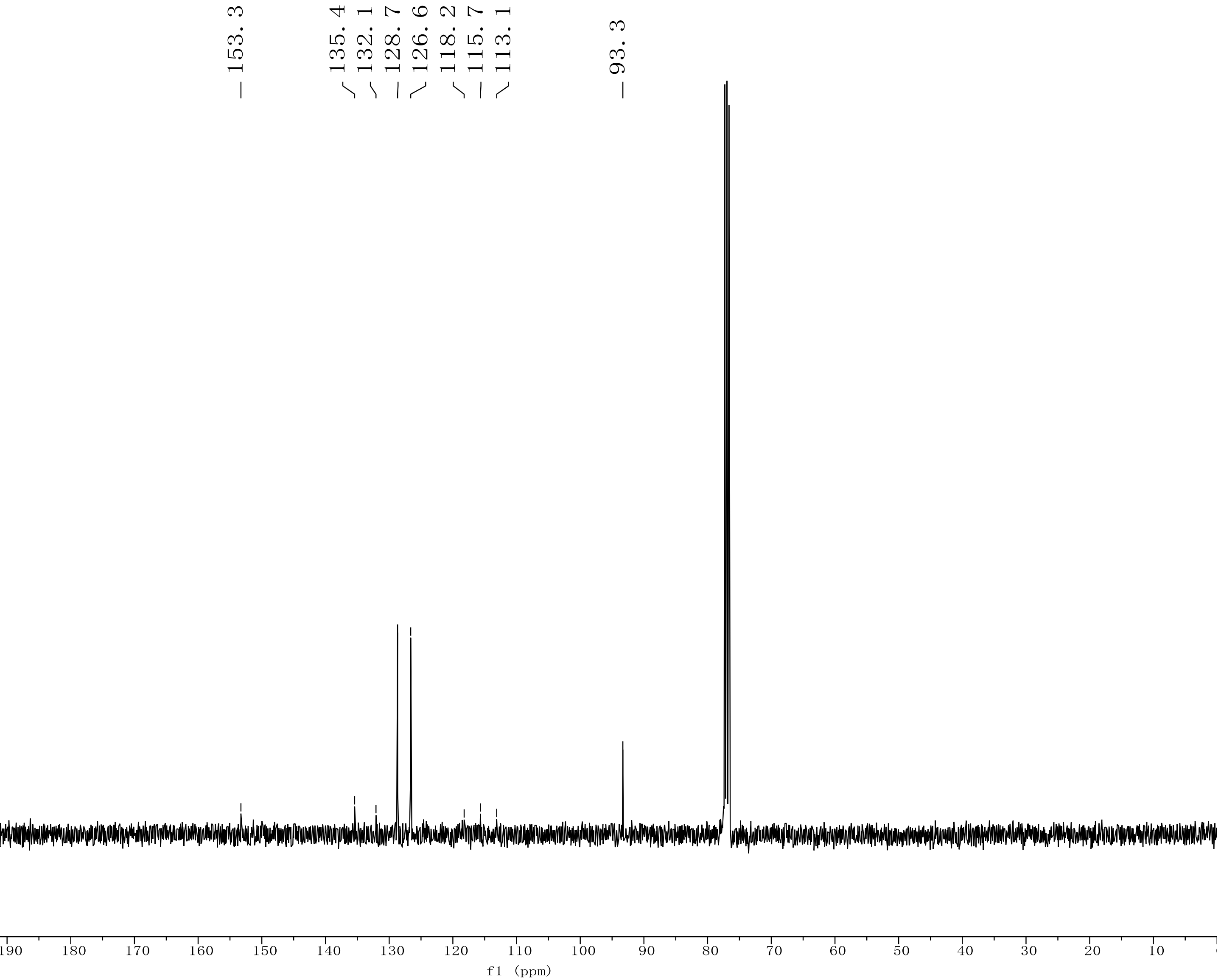

^{1}H NMR of 1-bromo-4-[1-(difluoromethoxy)vinyl]benzene (3d)

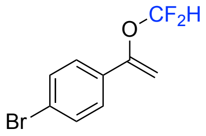

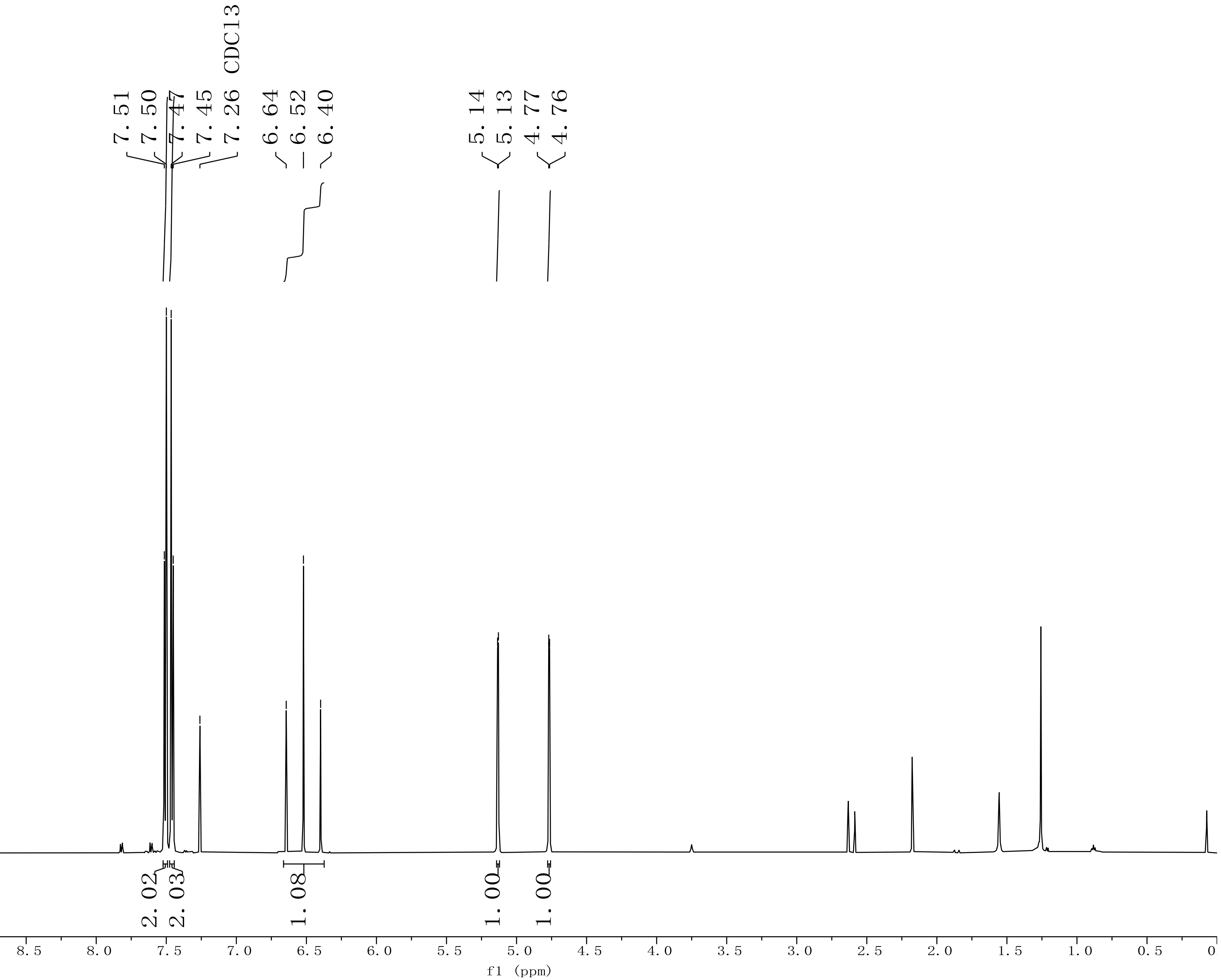

^{19}F NMR of 1-bromo-4-[1-(difluoromethoxy)vinyl]benzene (3d)

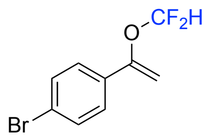

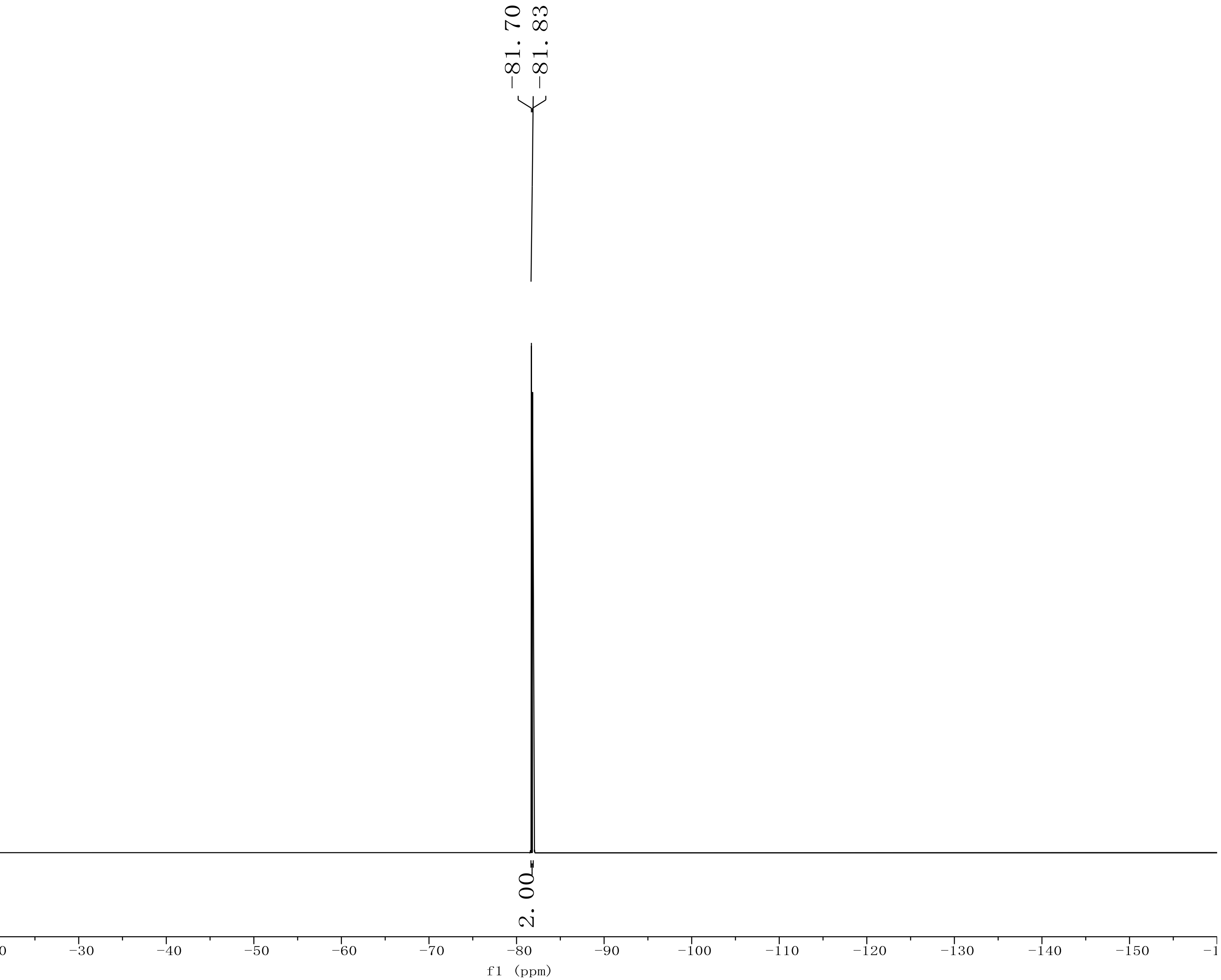

$^{13}C\{^{1}H\}$ NMR of 1-bromo-4-[1-(difluoromethoxy)vinyl]benzene (3d)

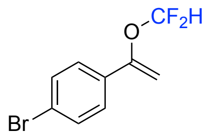

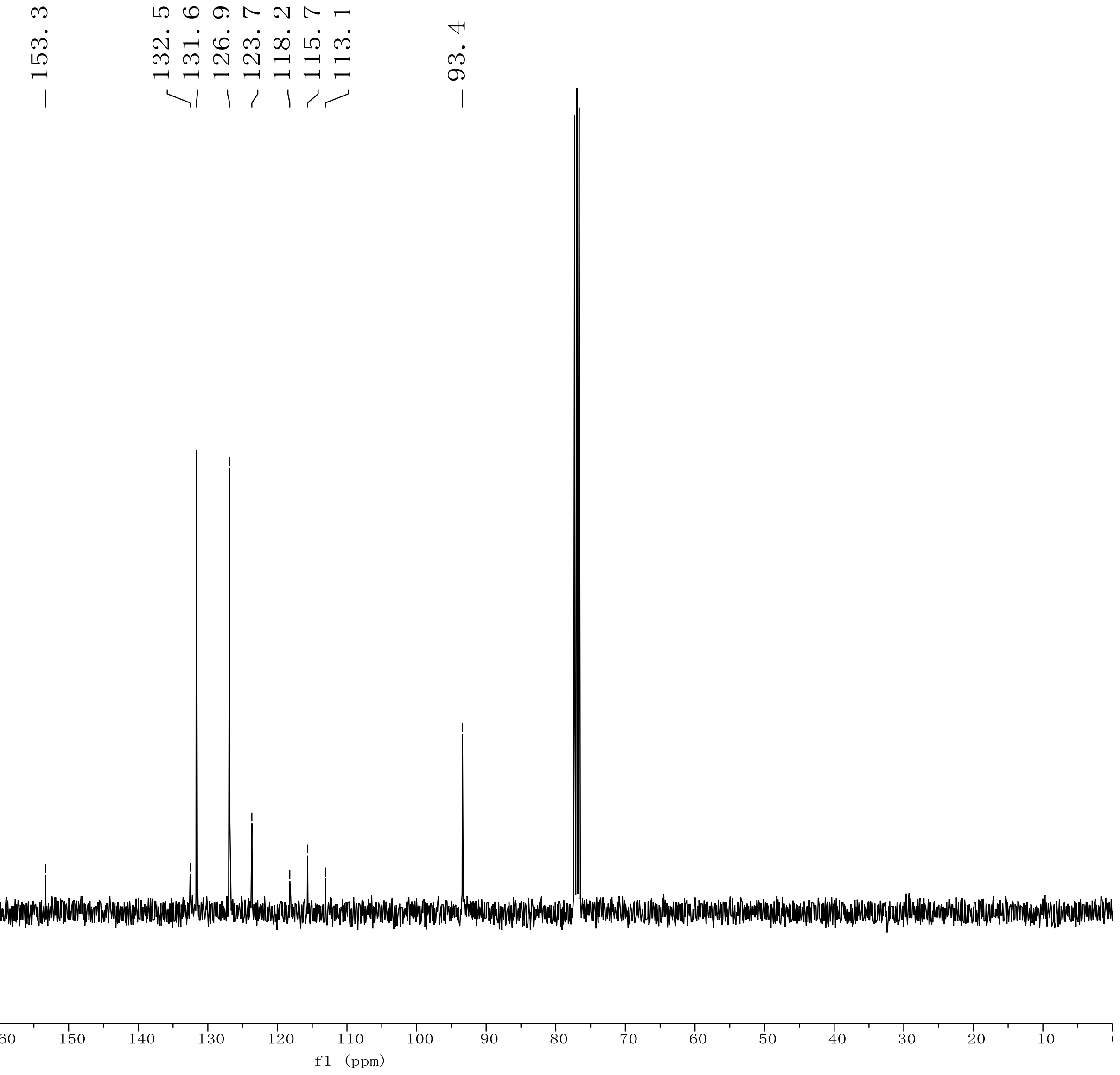

^{1}H NMR of 1-[1-(difluoromethoxy)vinyl]-4-isobutylbenzene (3e)

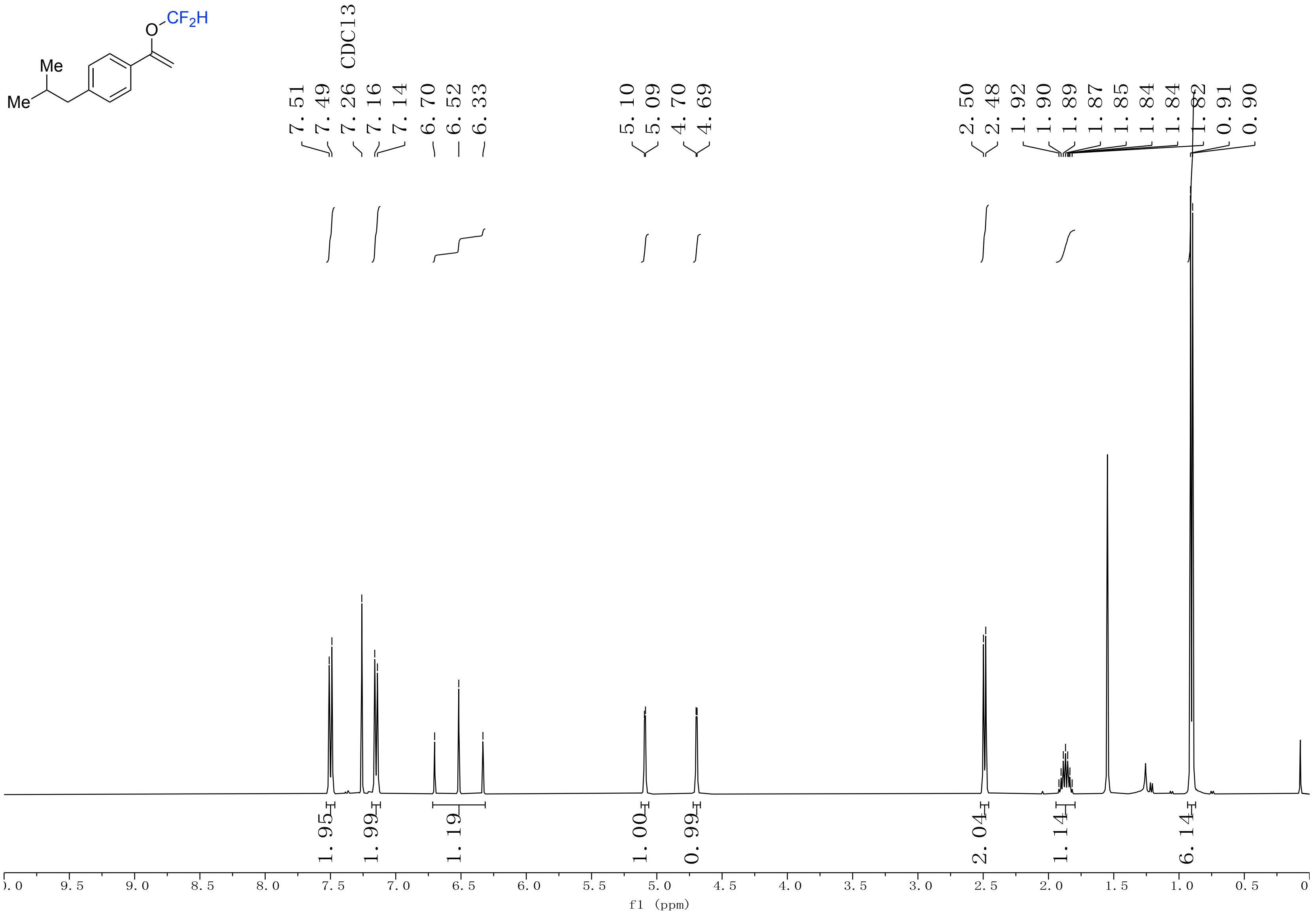

^{19}F NMR of 1-[1-(difluoromethoxy)vinyl]-4-isobutylbenzene (3e)

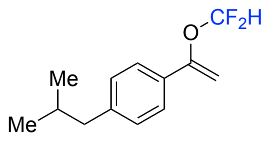

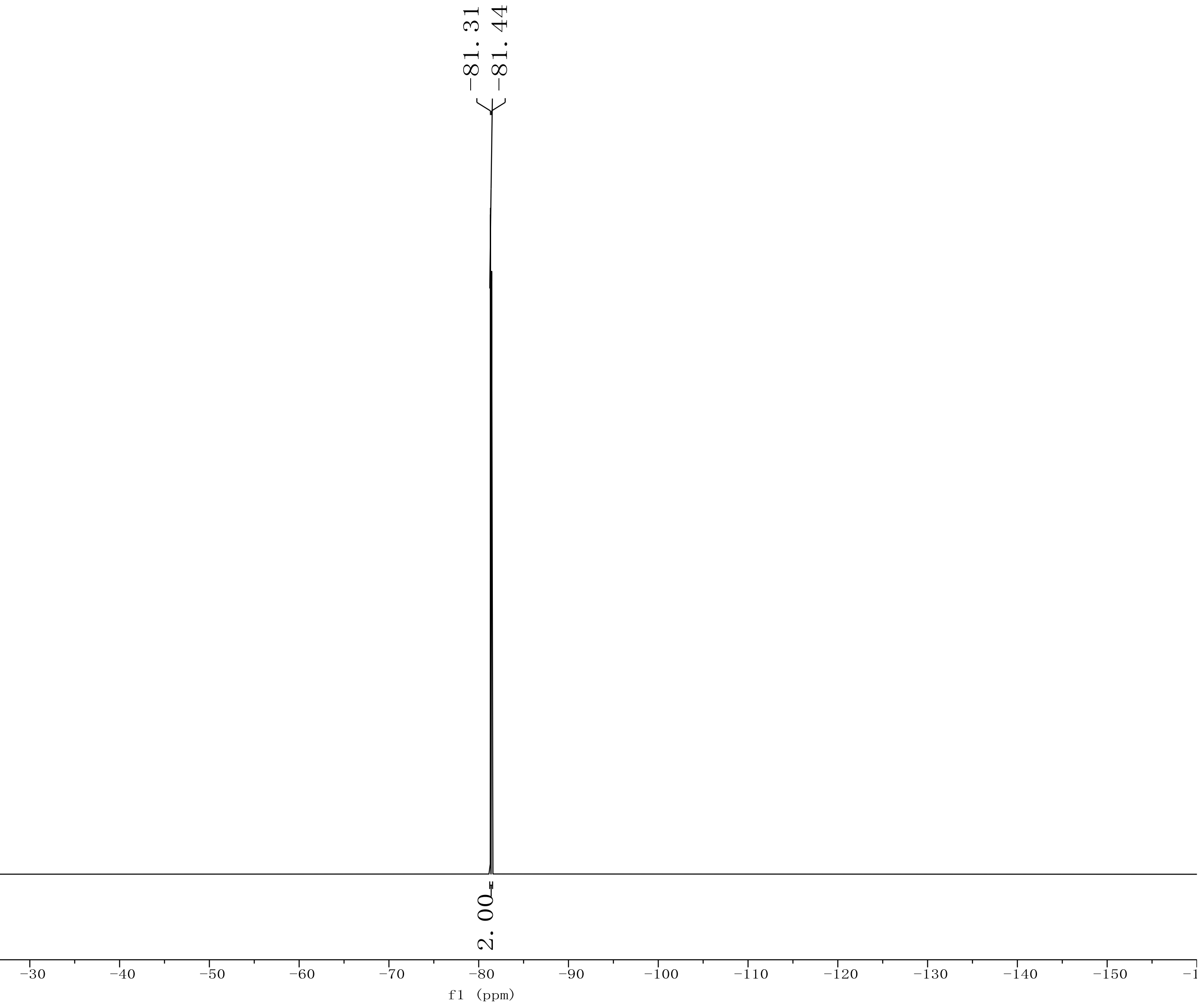

$^{13}C\{^1H\}$ NMR of 1-[1-(difluoromethoxy)vinyl]-4-isobutylbenzene (3e)

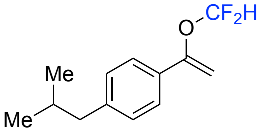

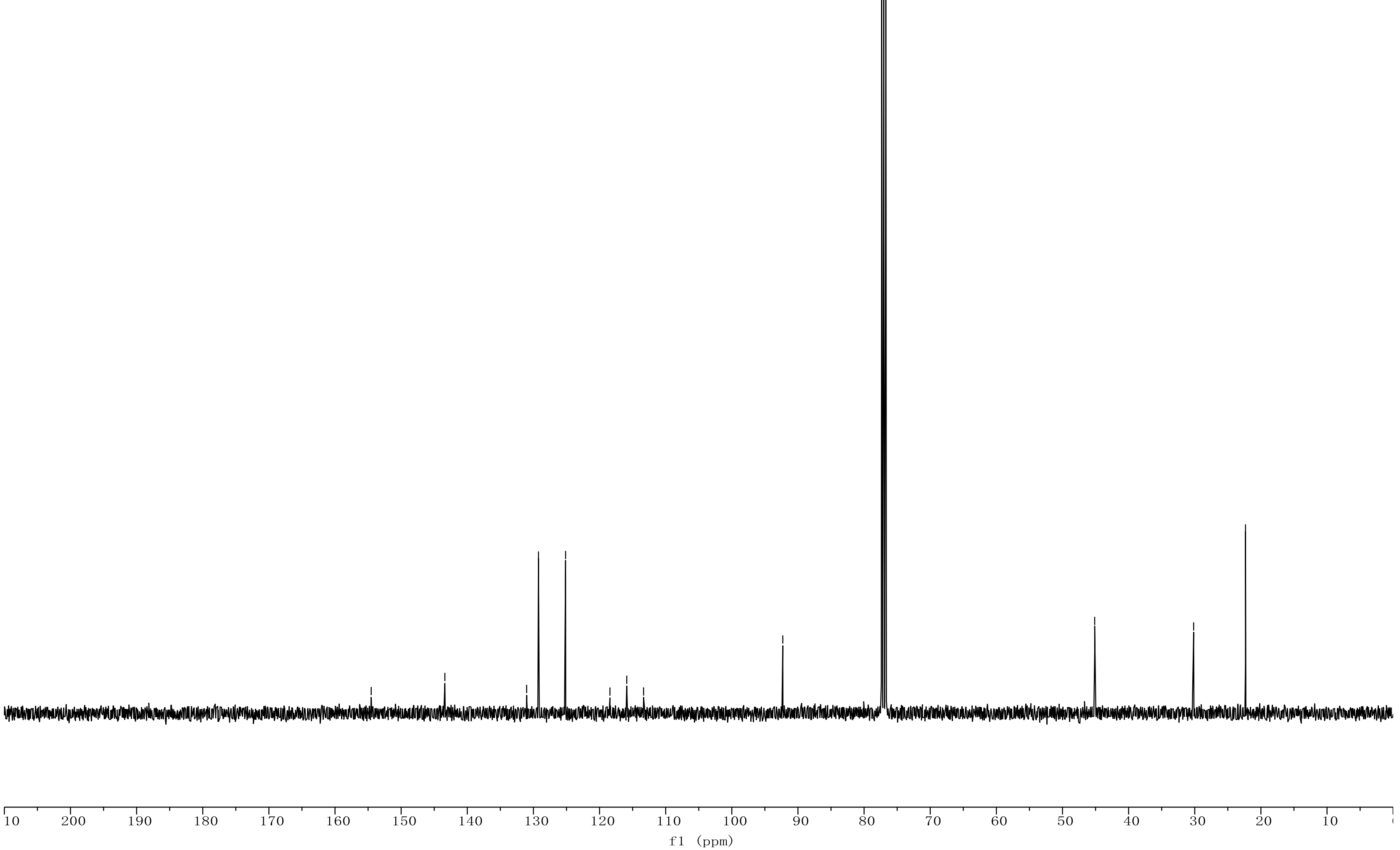

^{1}H NMR of the crude reaction mixture of 1-[1-(difluoromethoxy)vinyl]-2-methylbenzene (3f)

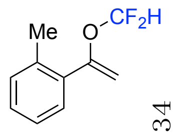

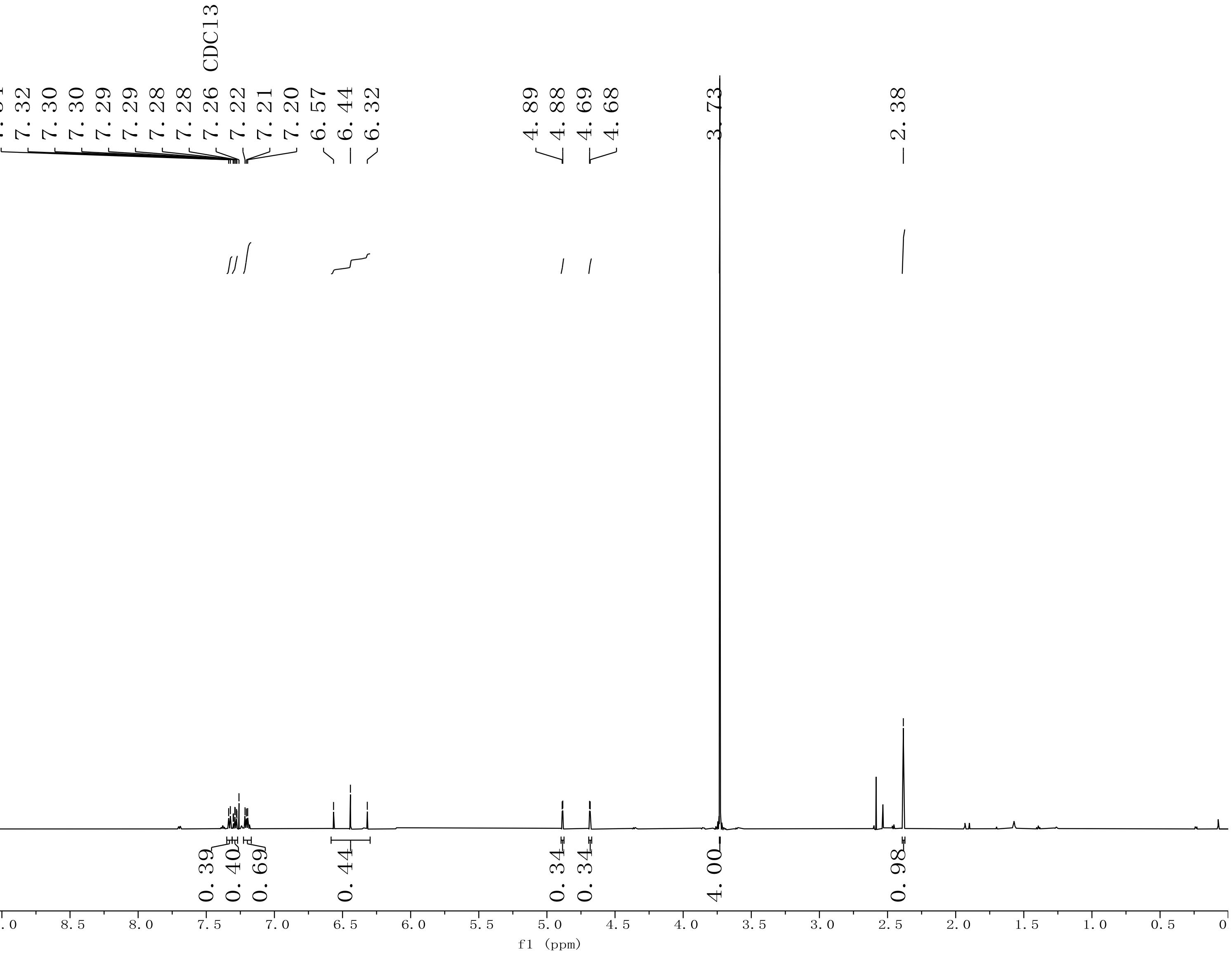

^{19}F NMR of the crude reaction mixture of 1-[1-(difluoromethoxy)vinyl]-2-methylbenzene (3f)

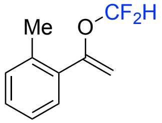

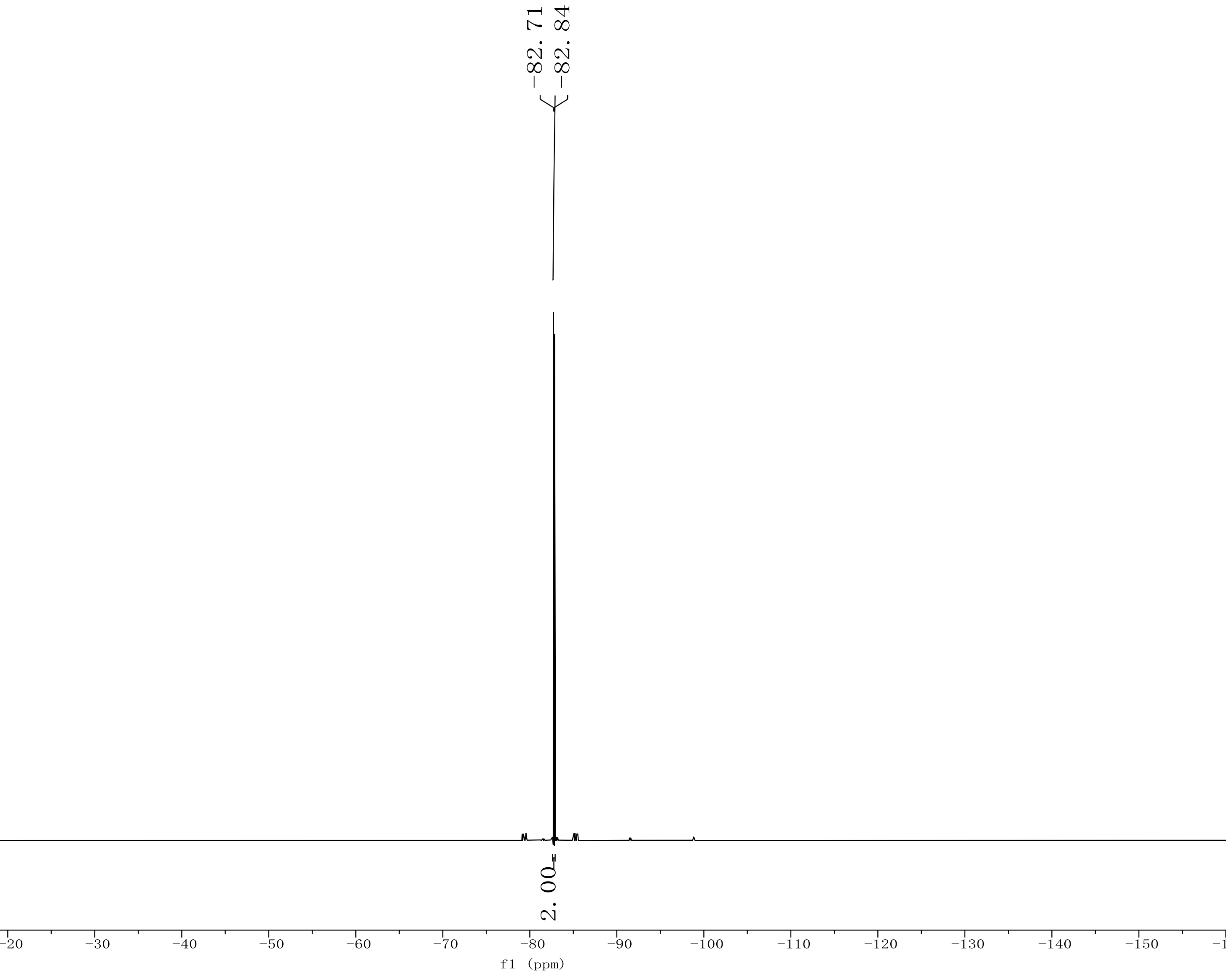

$^{13}C\{^1H\}$ NMR of the crude reaction mixture of 1-[1-(difluoromethoxy)vinyl]-2-methylbenzene (3f)

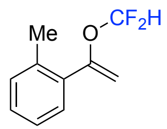

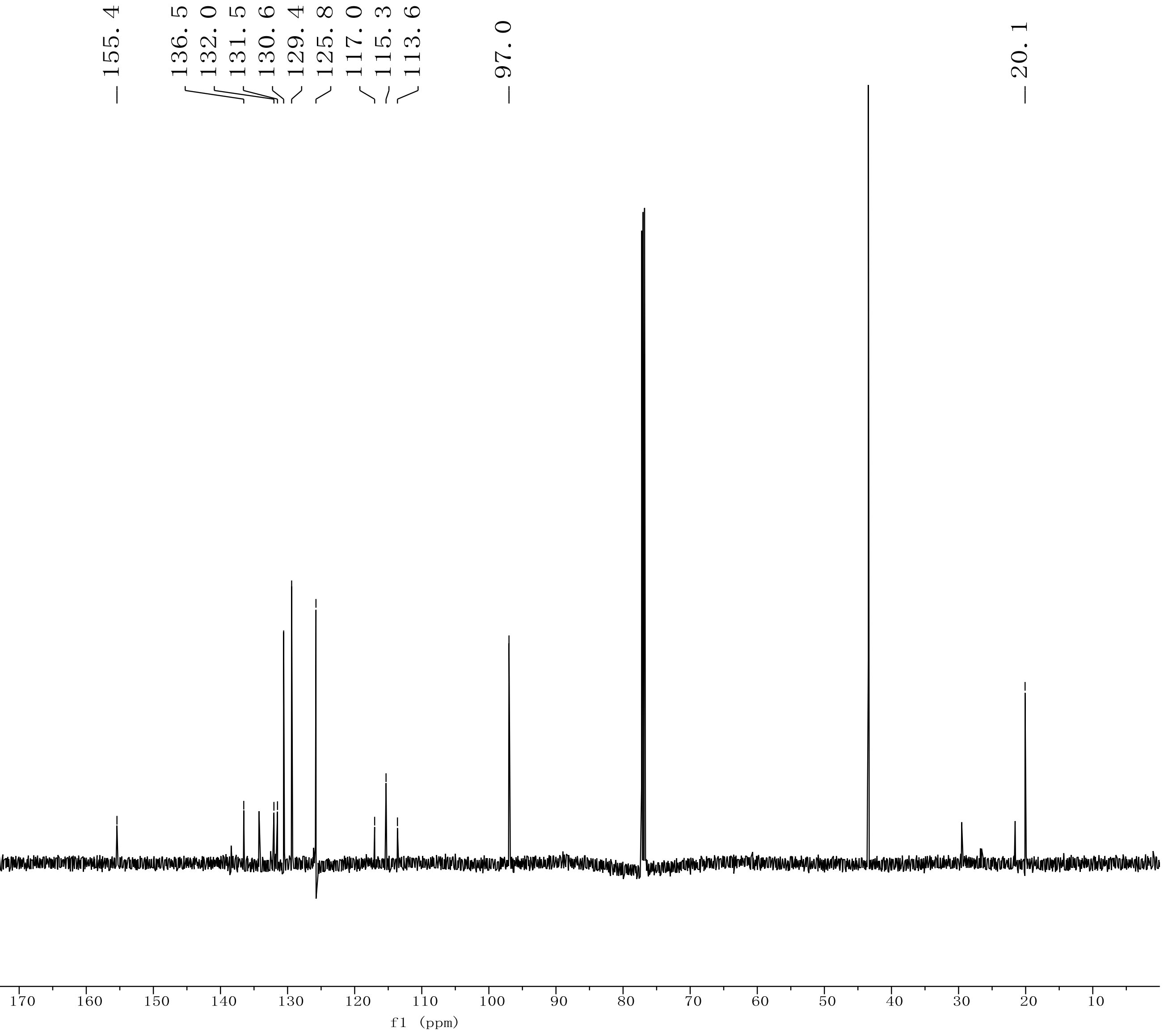

^{1}H NMR of the crude reaction mixture of 1-[1-(difluoromethoxy)vinyl]-2-methoxybenzene (3g)

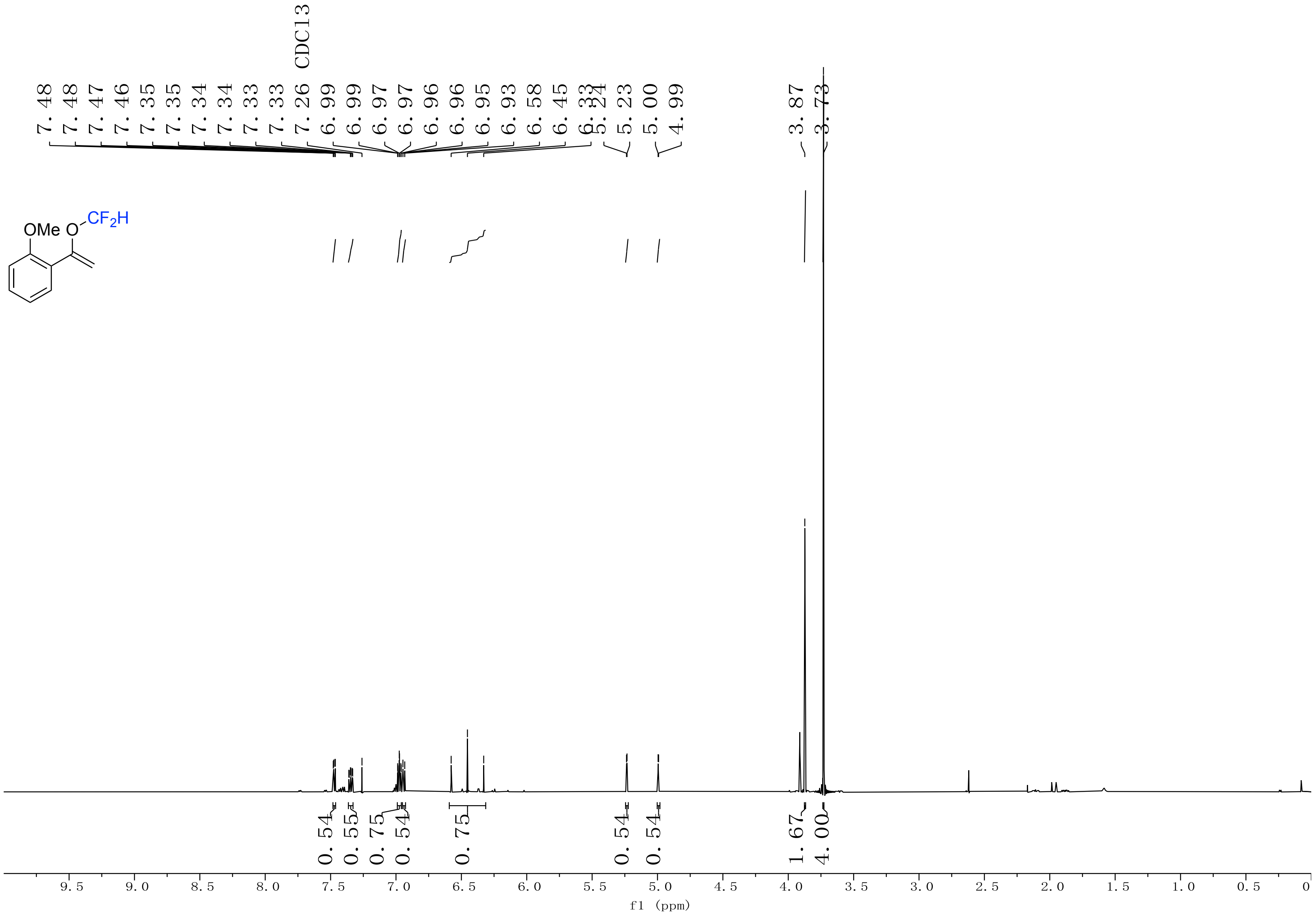

^{19}F NMR of the crude reaction mixture of 1-[1-(difluoromethoxy)vinyl]-2-methoxybenzene (3g)

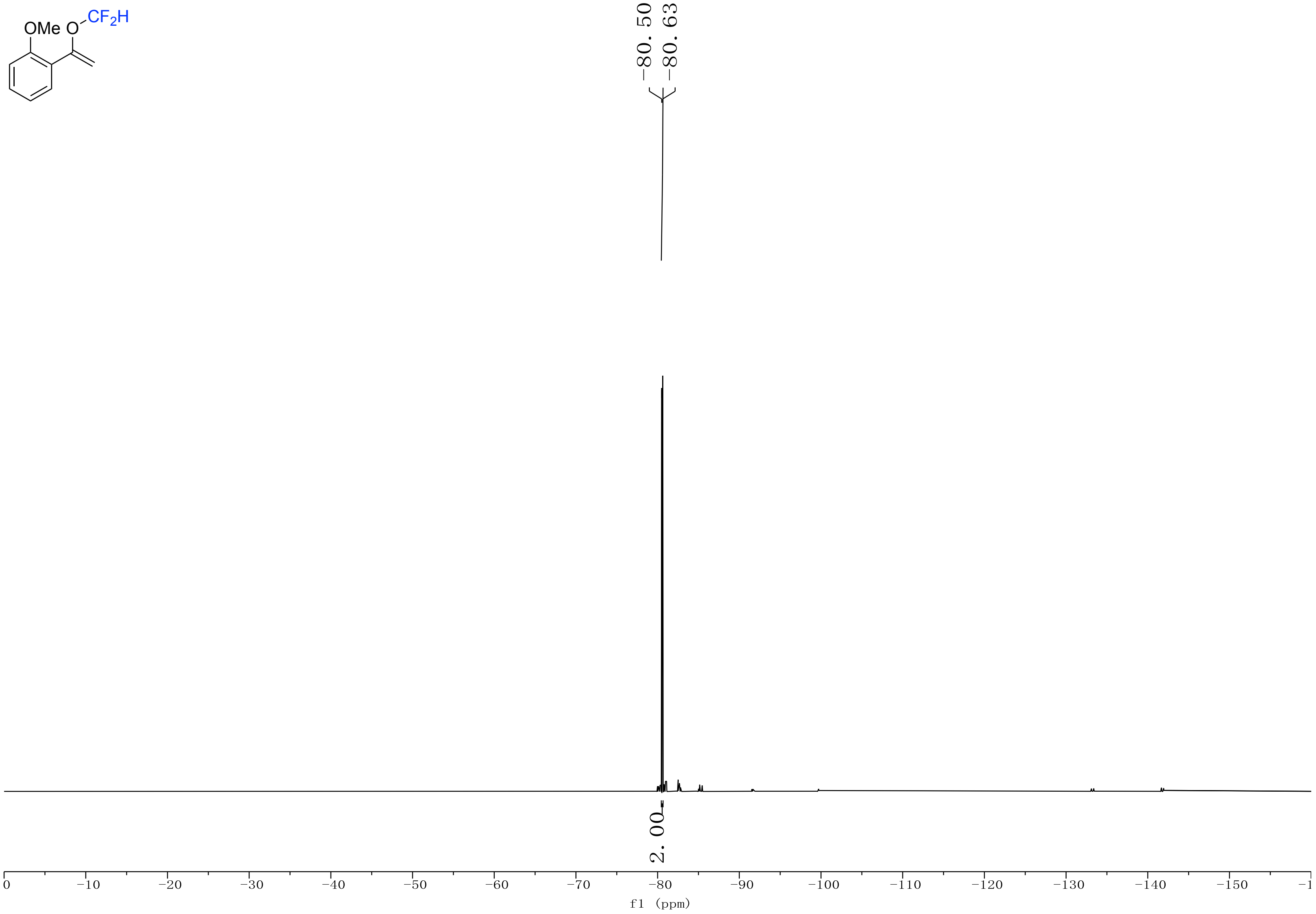

$^{13}C\{^1H\}$ NMR of the crude reaction mixture of 1-[1-(difluoromethoxy)vinyl]-2-methoxybenzene (3g)

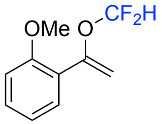

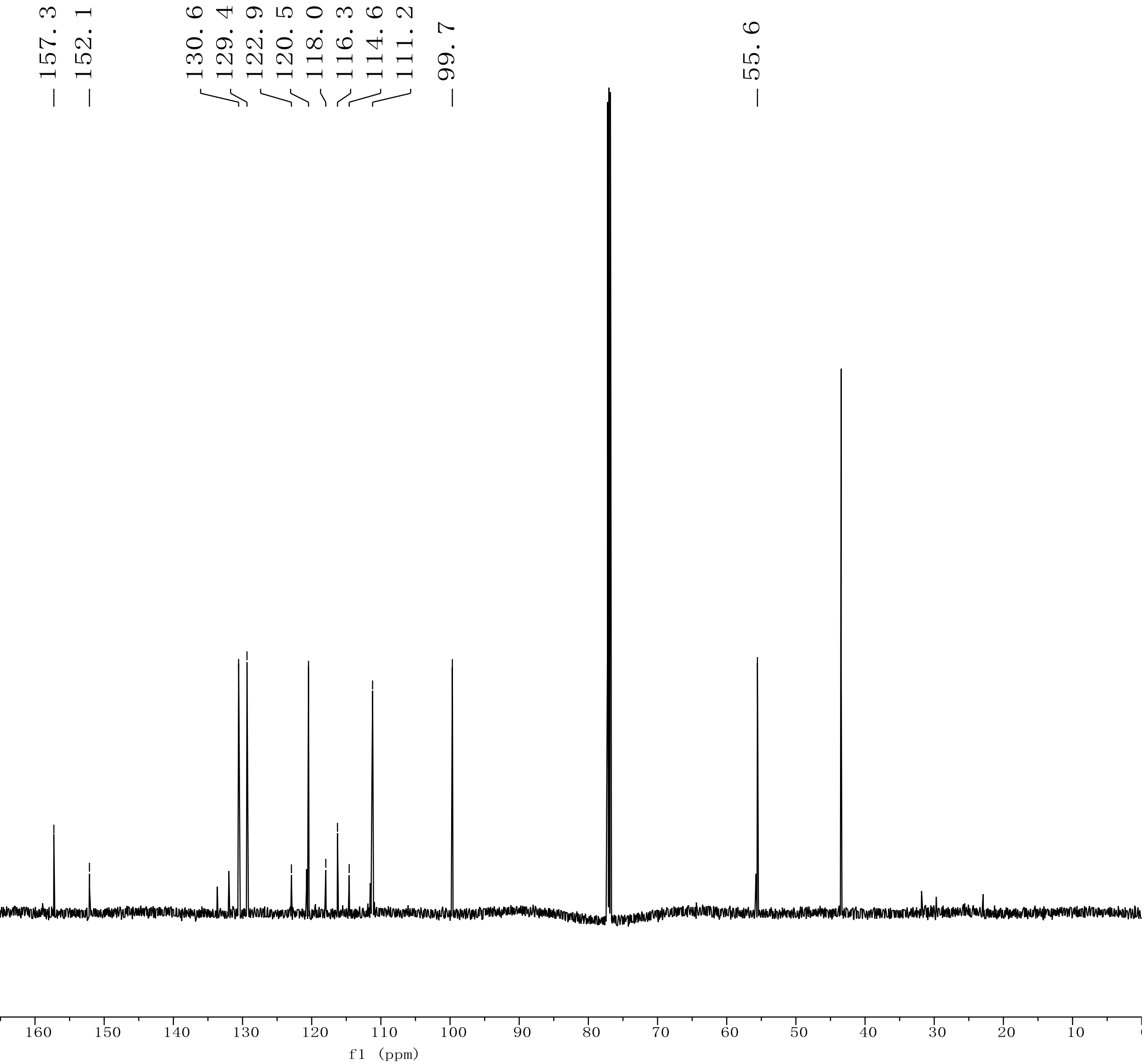

^{1}H NMR of 2-[1-(difluoromethoxy)vinyl]-1,3,5-trimethylbenzene (3h)

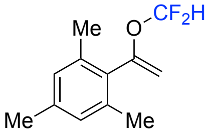

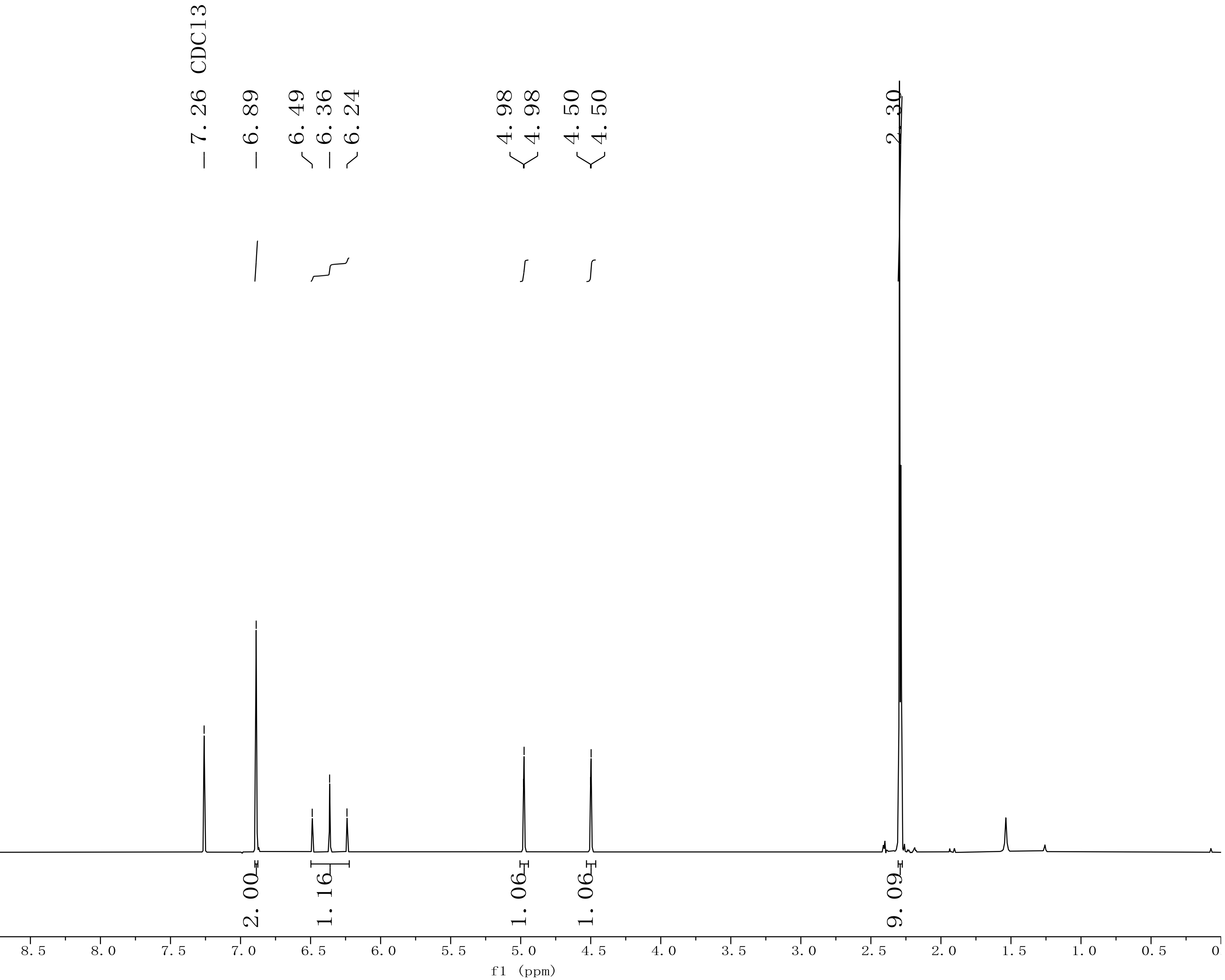

^{19}F NMR of 2-[1-(difluoromethoxy)vinyl]-1,3,5-trimethylbenzene (3h)

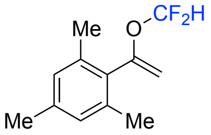

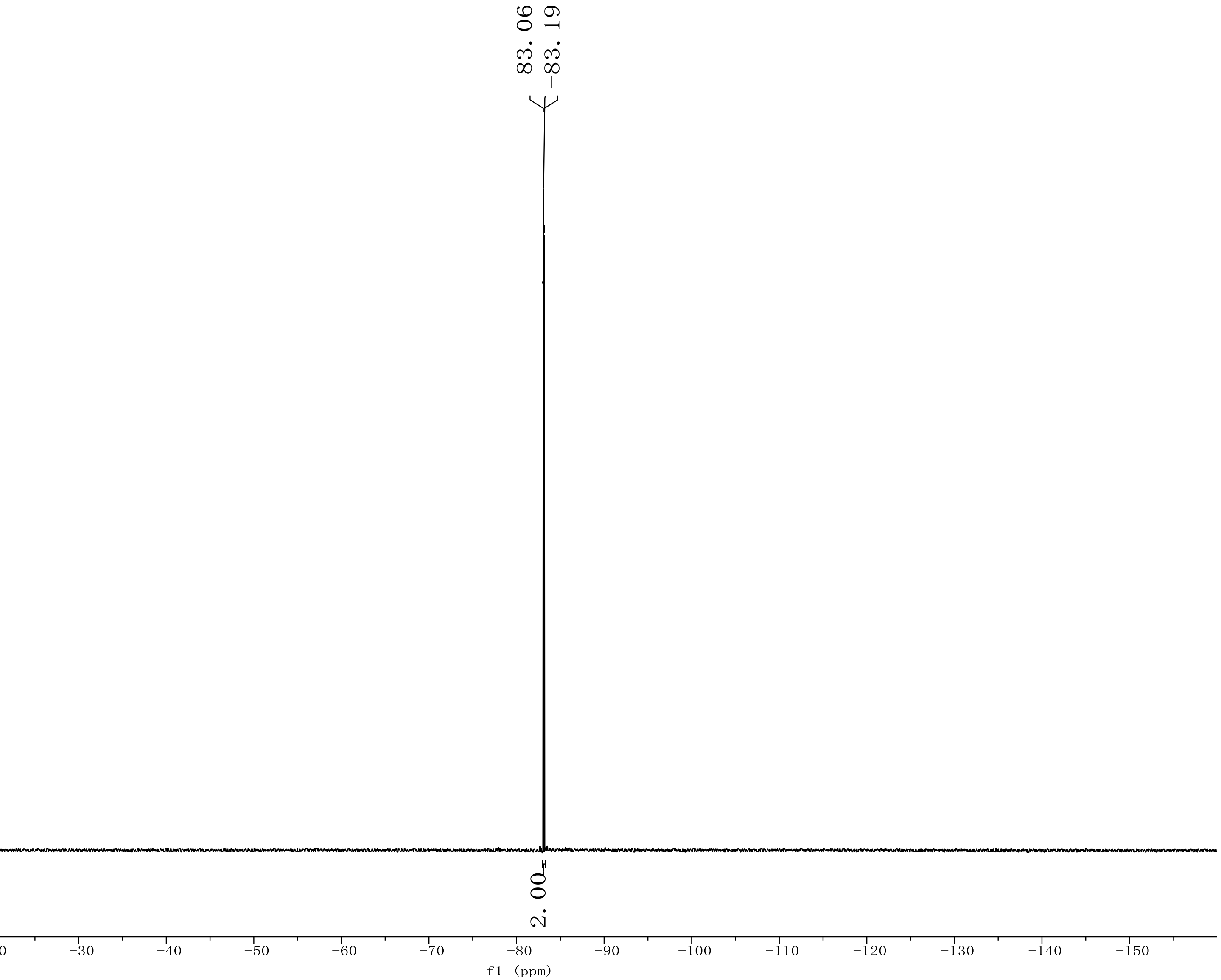

$^{13}C\{^1H\}$ NMR of 2-[1-(difluoromethoxy)vinyl]-1,3,5-trimethylbenzene (3h)

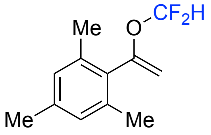

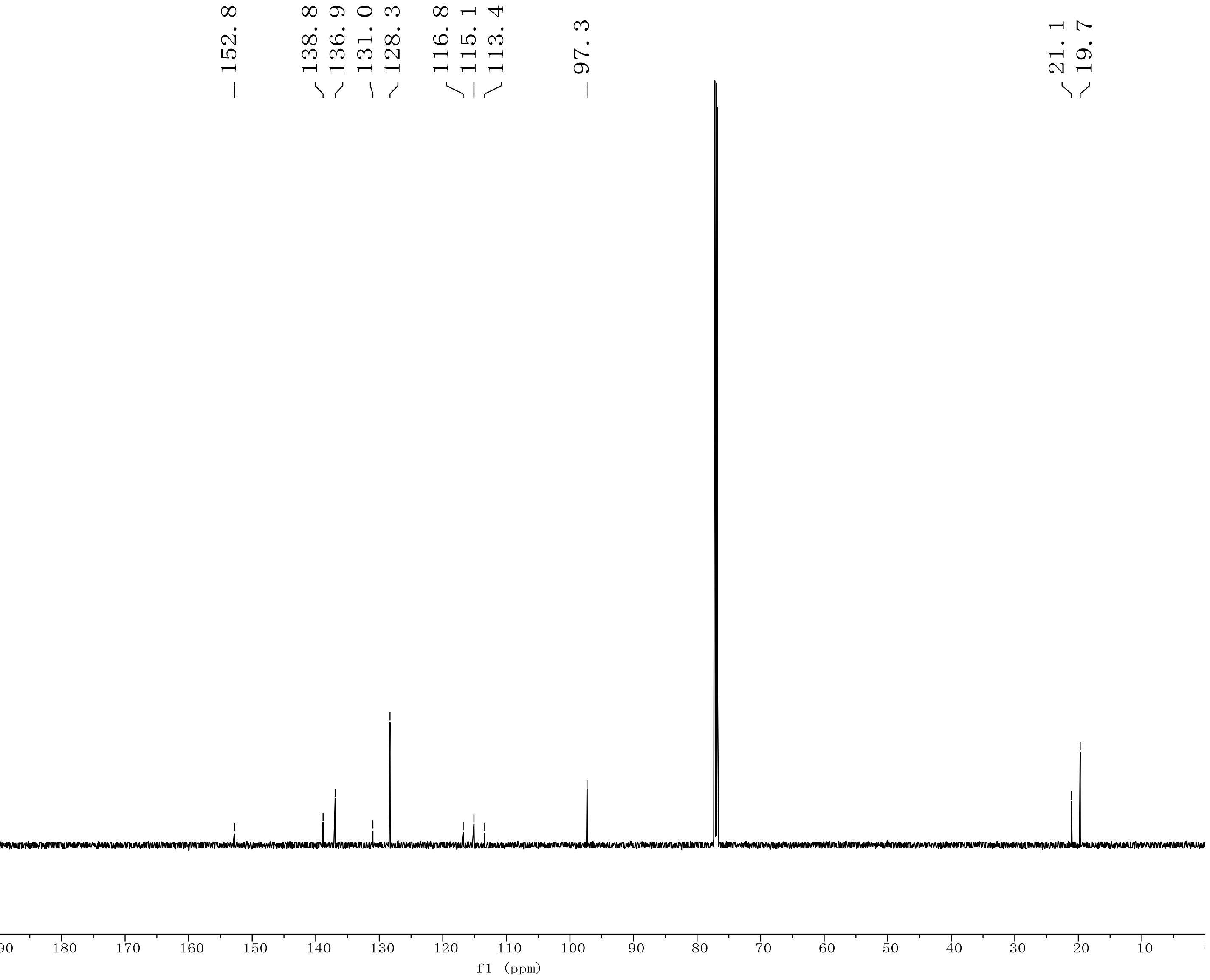

^1H NMR of the crude reaction mixture of 2-[1-(difluoromethoxy)vinyl]naphthalene (3i)

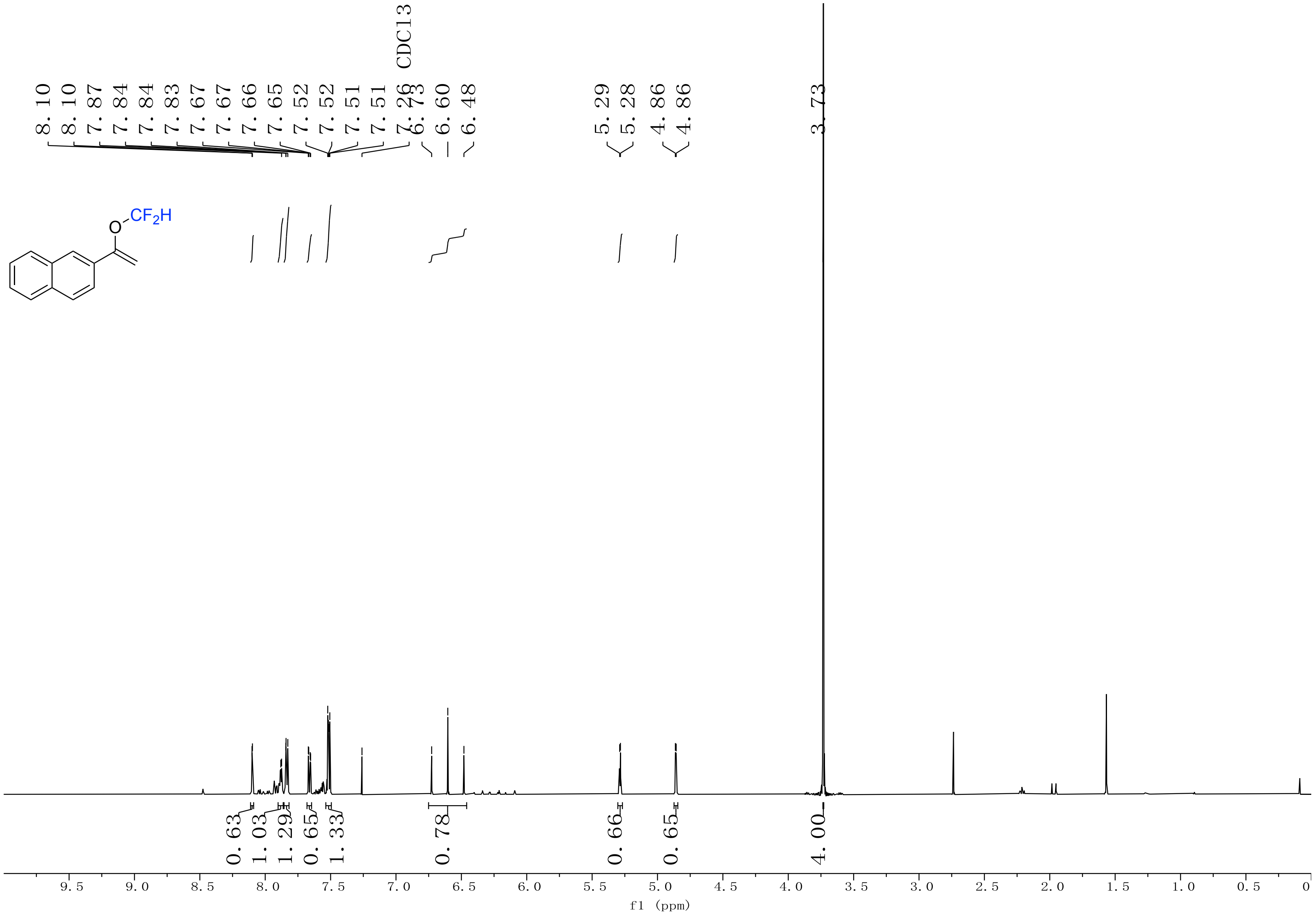

^{19}F NMR of the crude reaction mixture of 2-[1-(difluoromethoxy)vinyl]naphthalene (3i)

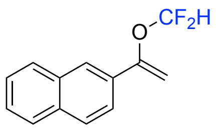

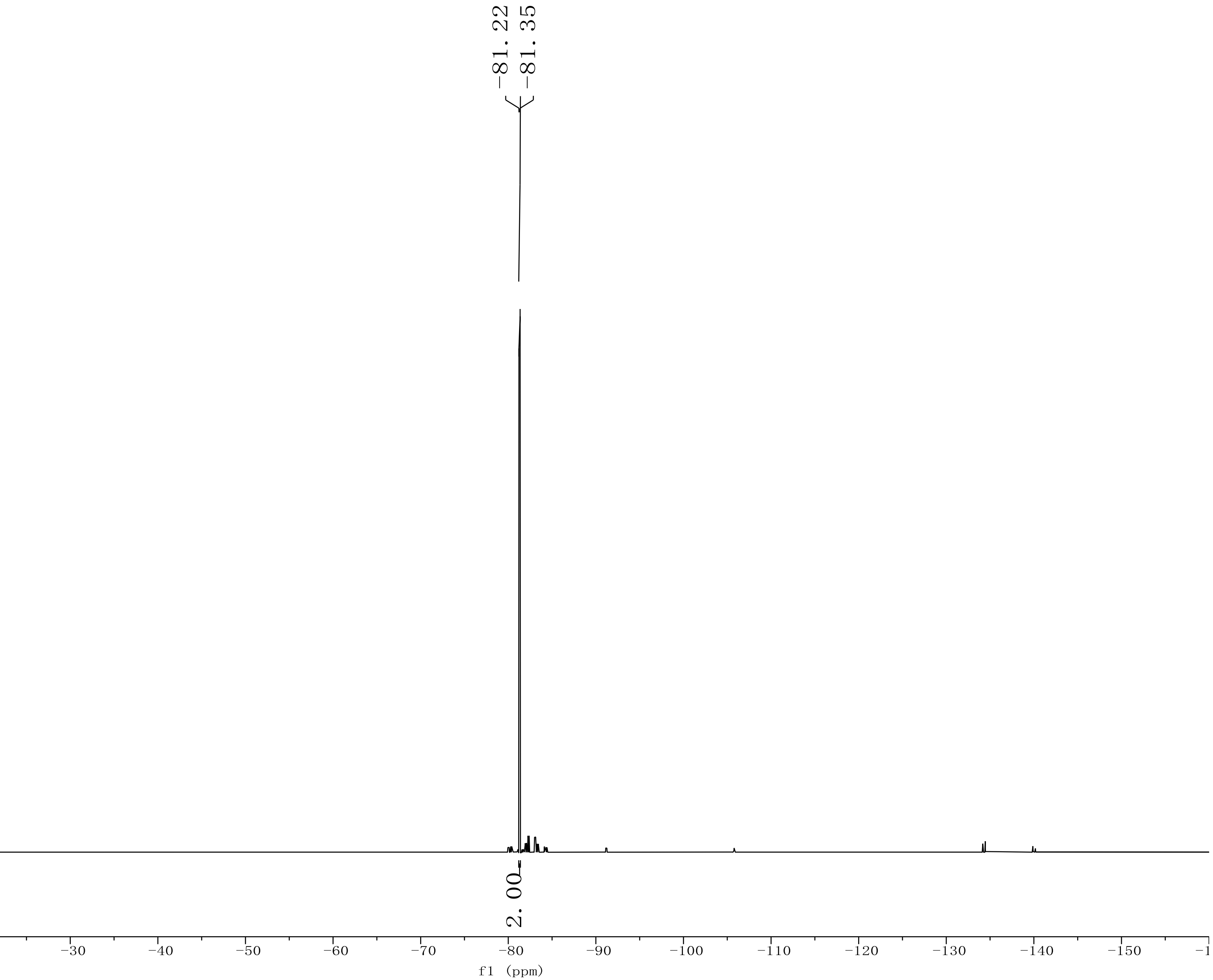

$^{13}C\{^1H\}$ NMR of the crude reaction mixture of 2-[1-(difluoromethoxy)vinyl]naphthalene (3i)

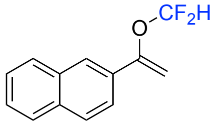

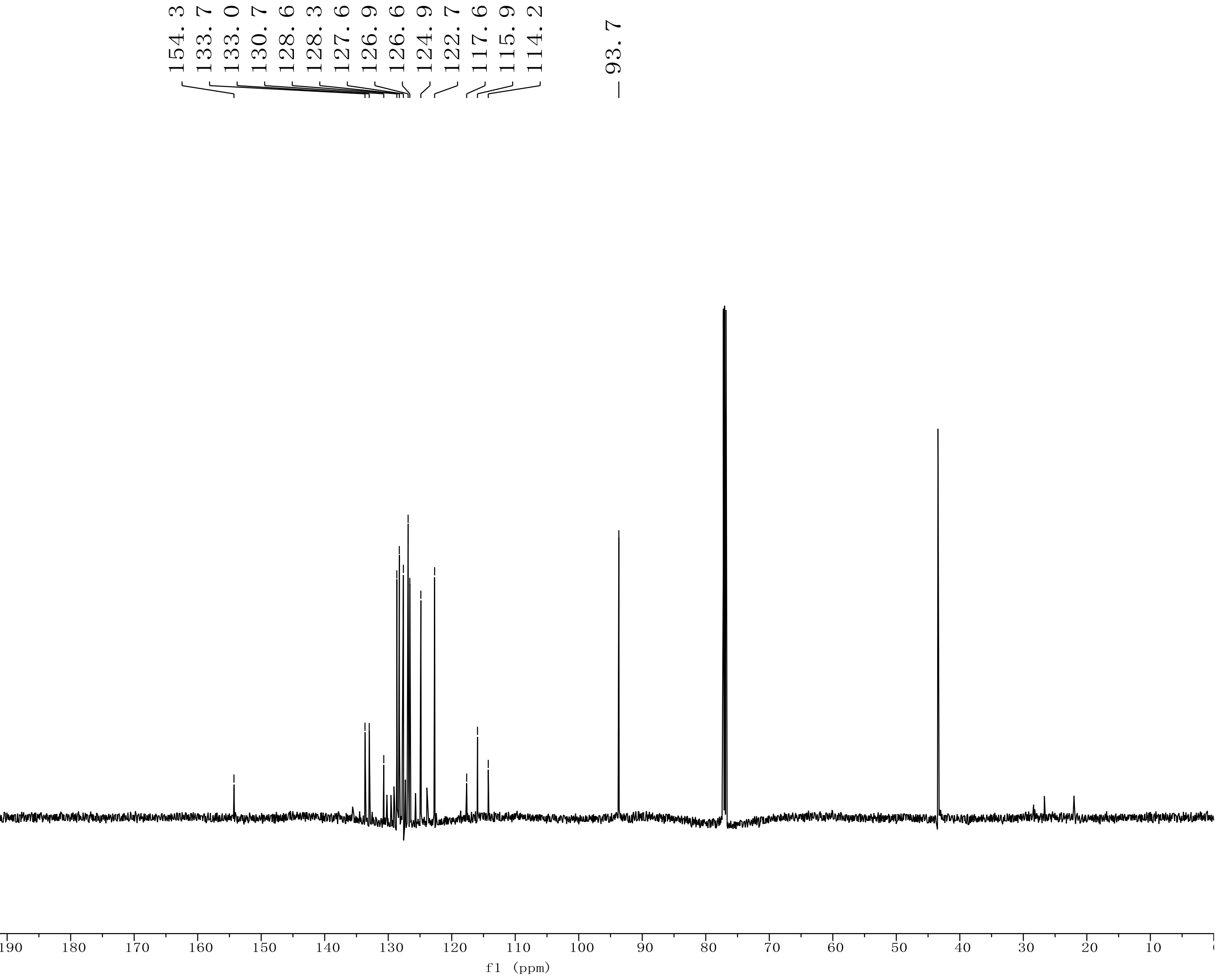

^{1}H NMR of the crude reaction mixture of [3-(difluoromethoxy)but-3-en-1-yl]benzene (3j)

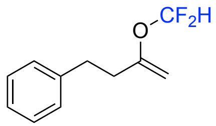

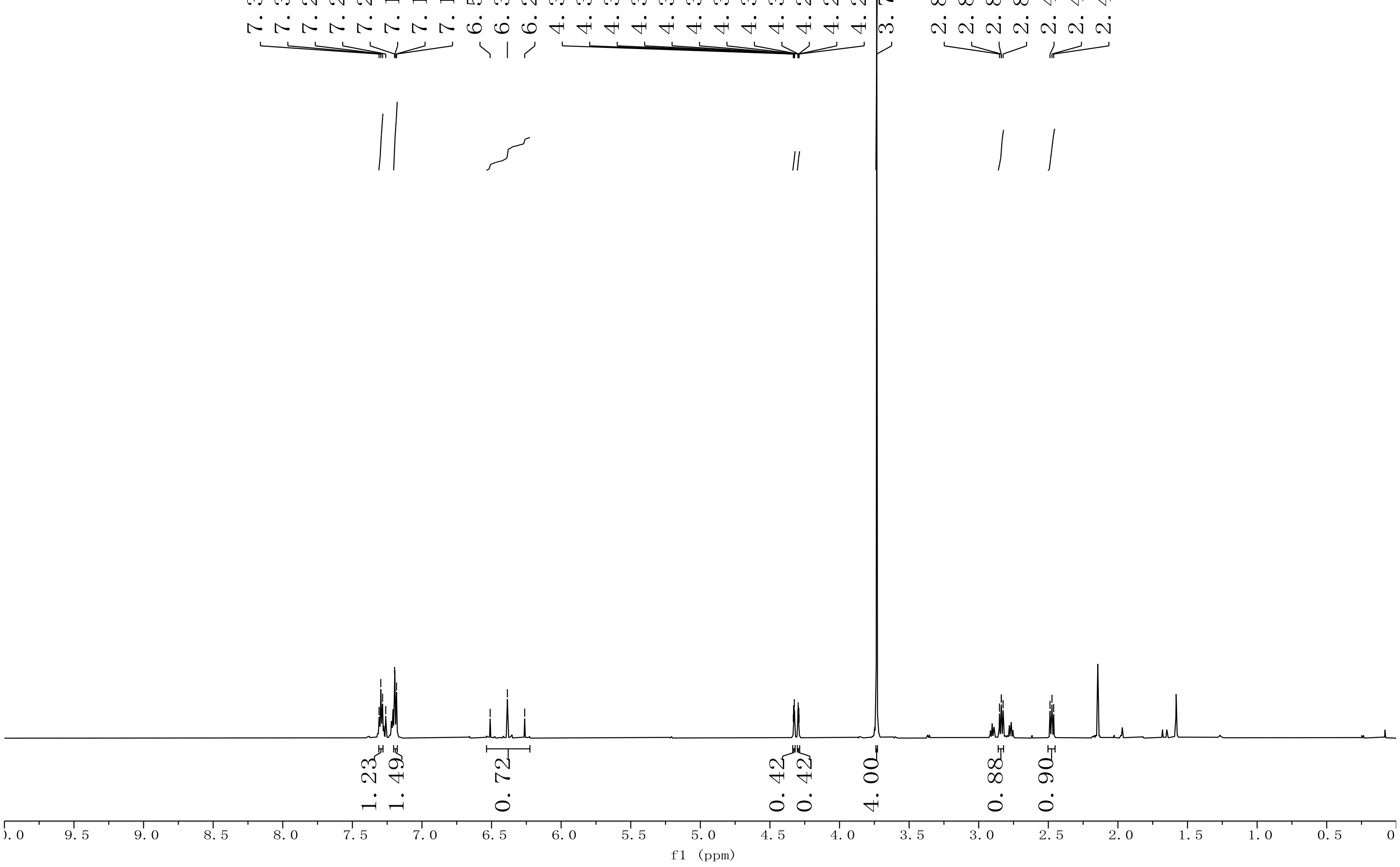

^{19}F NMR of the crude reaction mixture of [3-(difluoromethoxy)but-3-en-1-yl]benzene (3j)

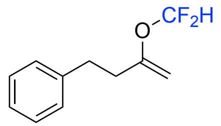

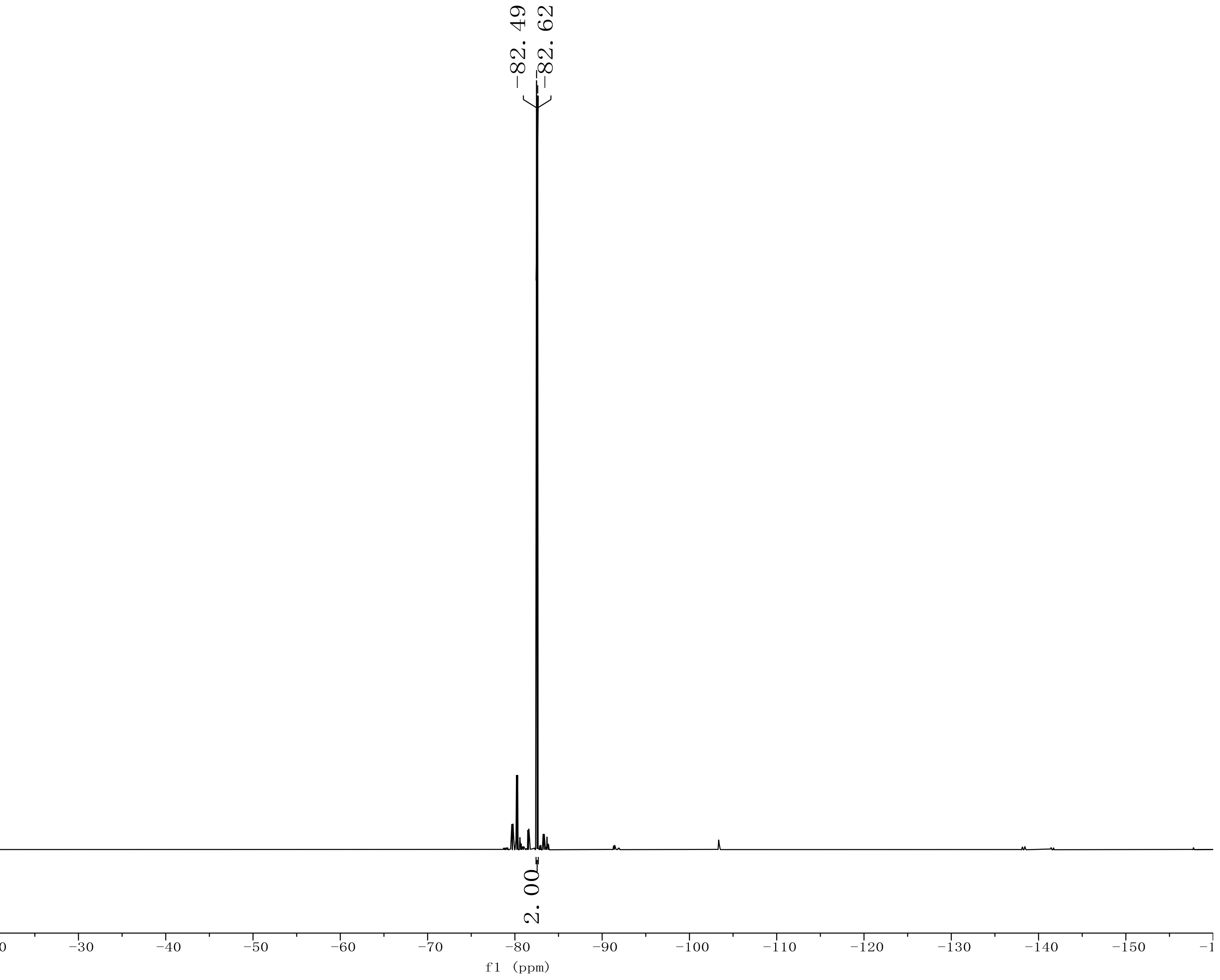

$^{13}C\{^1H\}$ NMR of the crude reaction mixture of [3-(difluoromethoxy)but-3-en-1-yl]benzene (3j)

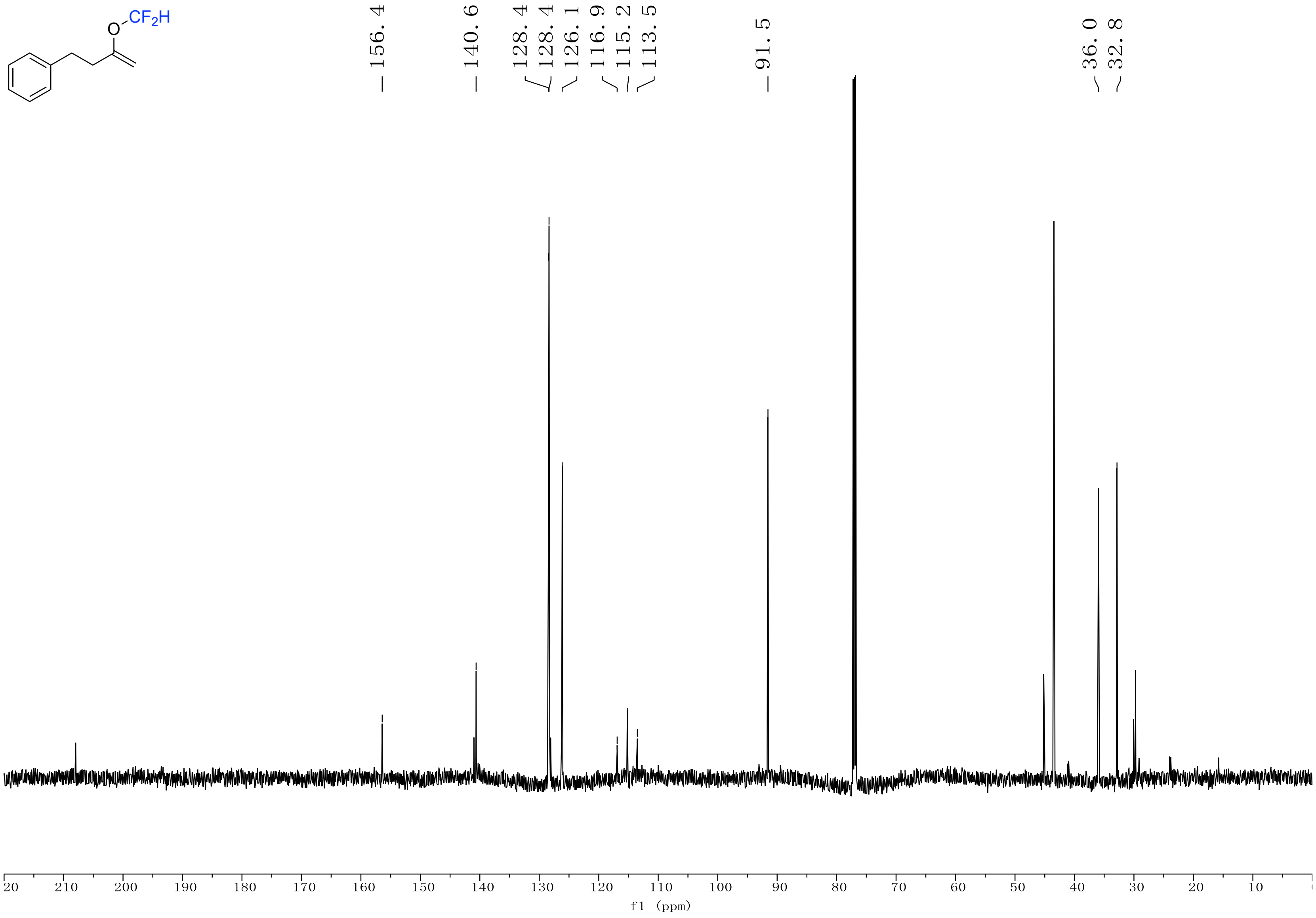

^{1}H NMR of 2,4-dichloro-1-[3-(difluoromethoxy)buta-1,3-dien-1-yl]benzene (3k)

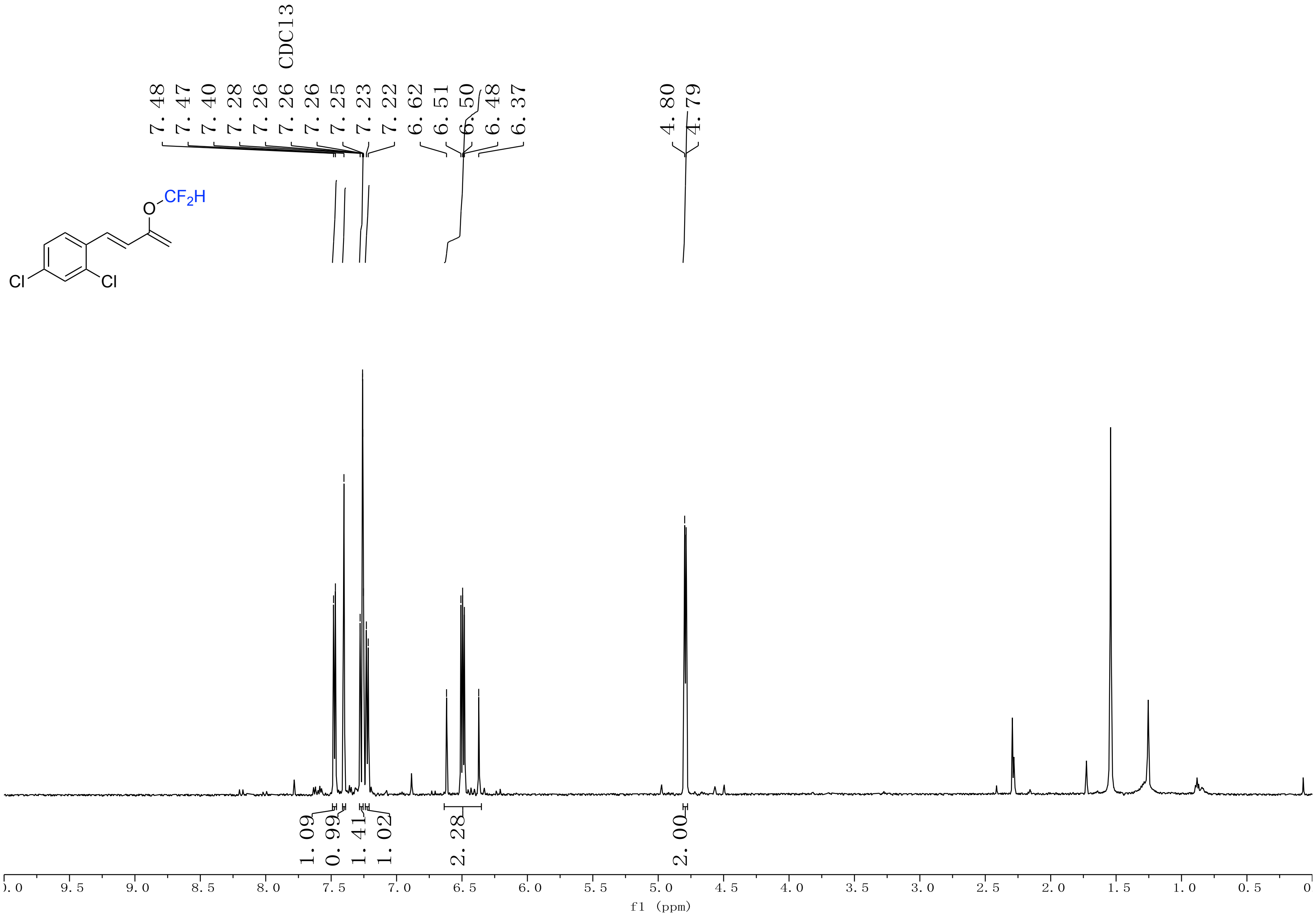

^{19}F NMR of 2,4-dichloro-1-[3-(difluoromethoxy)buta-1,3-dien-1-yl]benzene (3k)

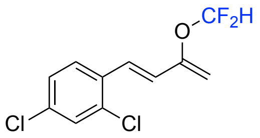

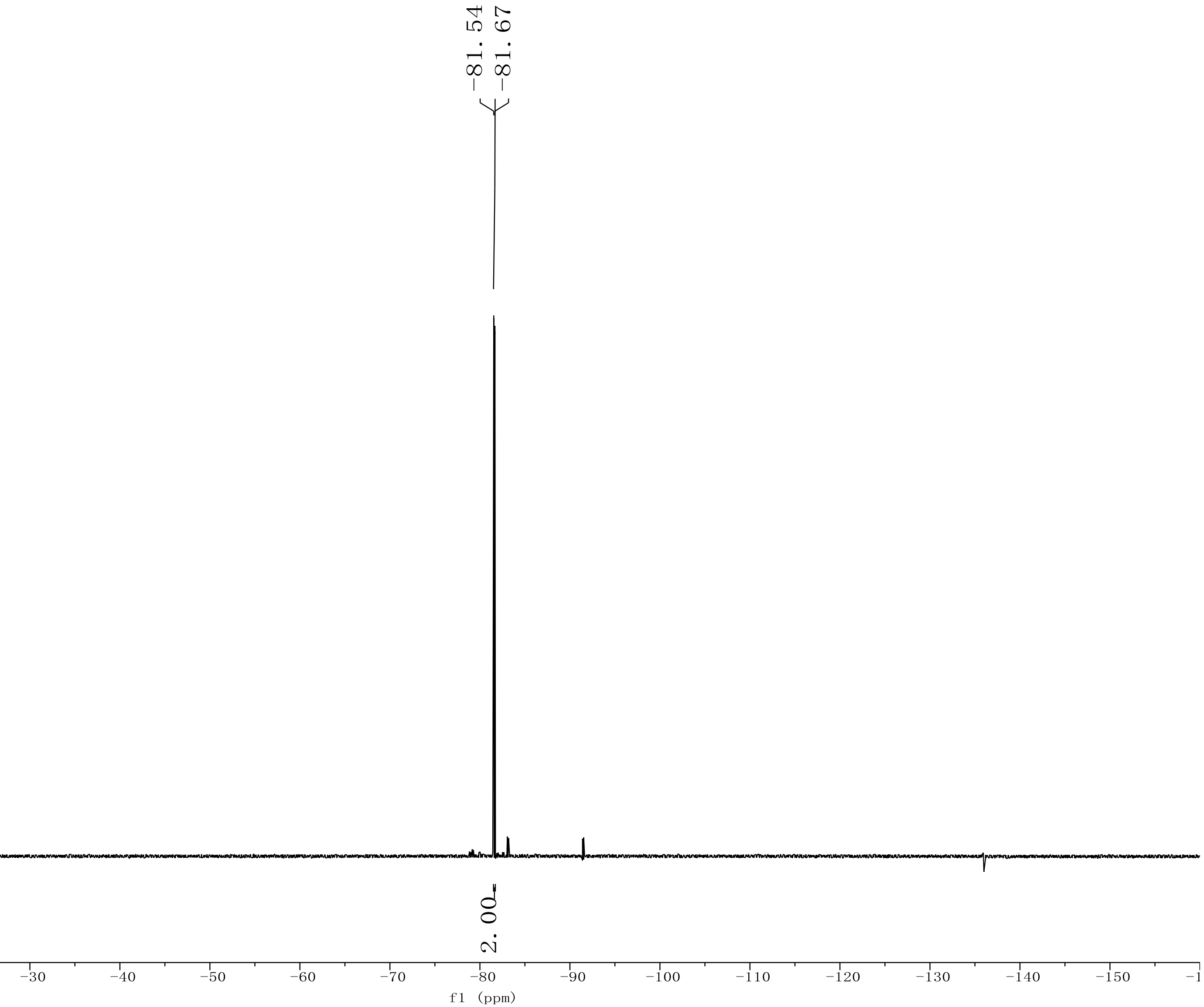

$^{13}C\{^{1}H\}$ NMR of 2,4-dichloro-1-[3-(difluoromethoxy)buta-1,3-dien-1-yl]benzene (3k)

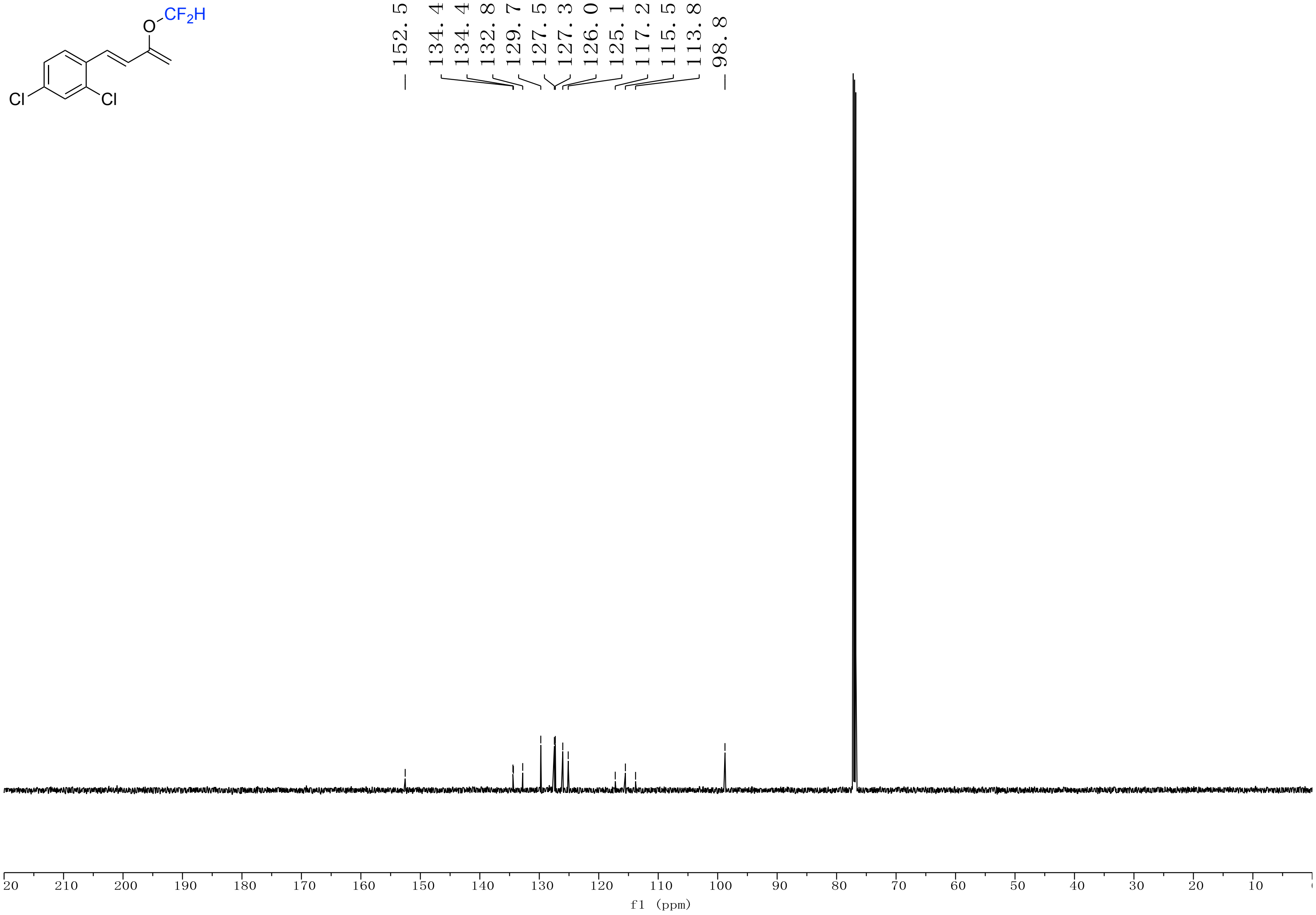

^{1}H NMR of 3-(difluoromethoxy)-1-phenylbut-2-en-1-one (3l)

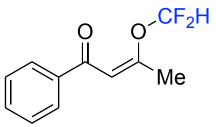

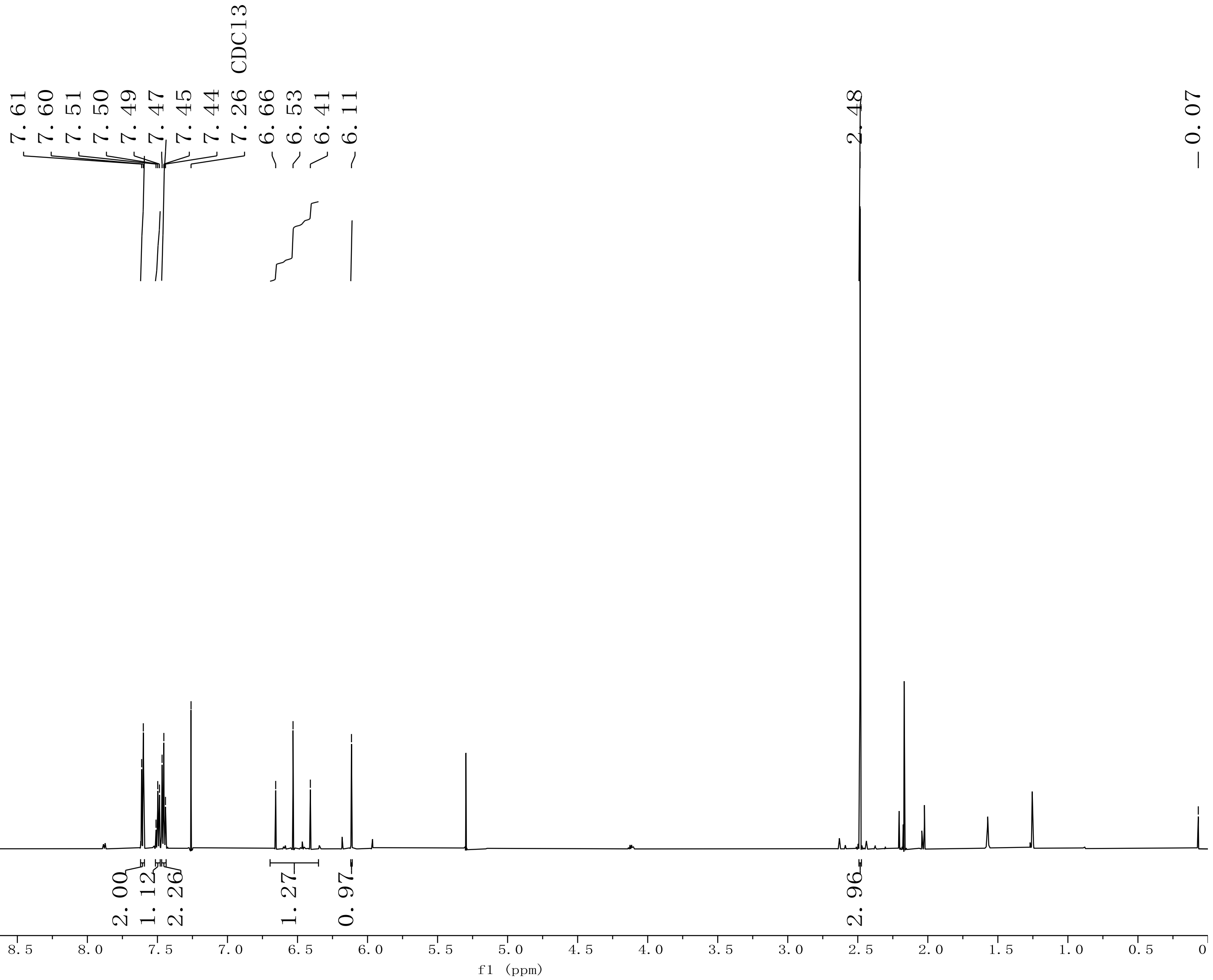

^{19}F NMR of 3-(difluoromethoxy)-1-phenylbut-2-en-1-one (3l)

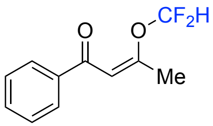

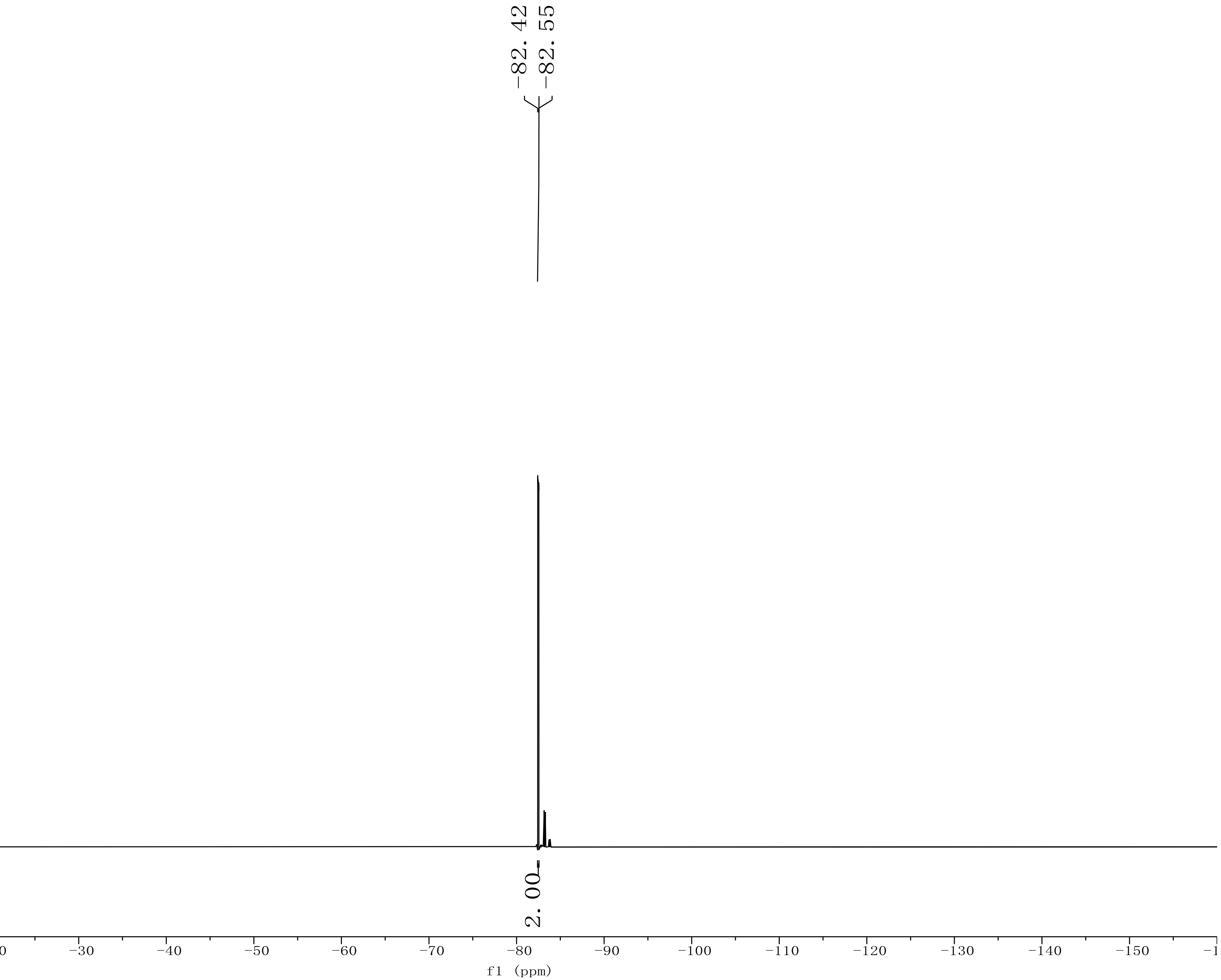

$^{13}C\{^{1}H\}$ NMR of 3-(difluoromethoxy)-1-phenylbut-2-en-1-one (3l)

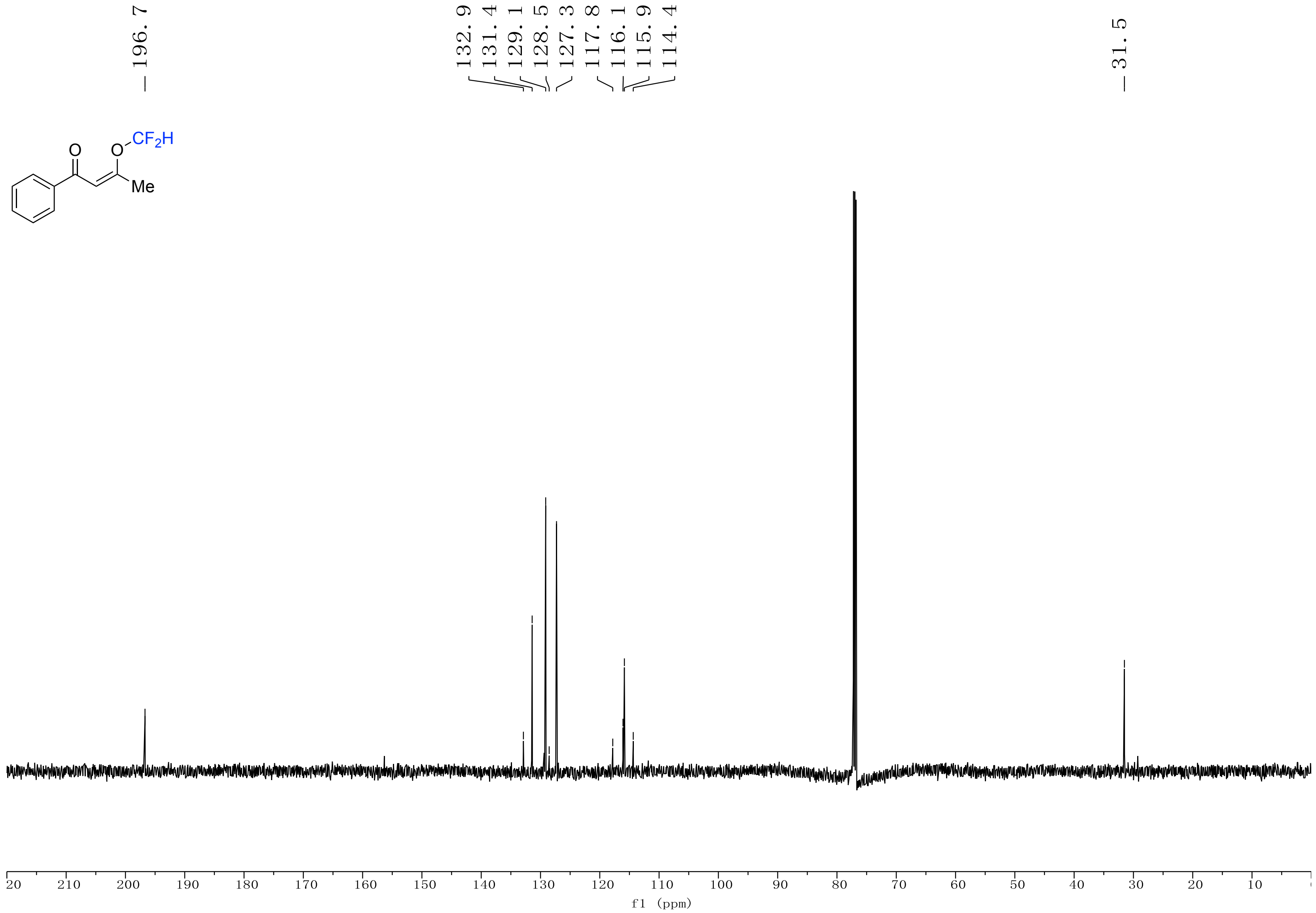

^{1}H NMR of the crude reaction mixture of 4-(difluoromethoxy)-2H-chromene (3m)

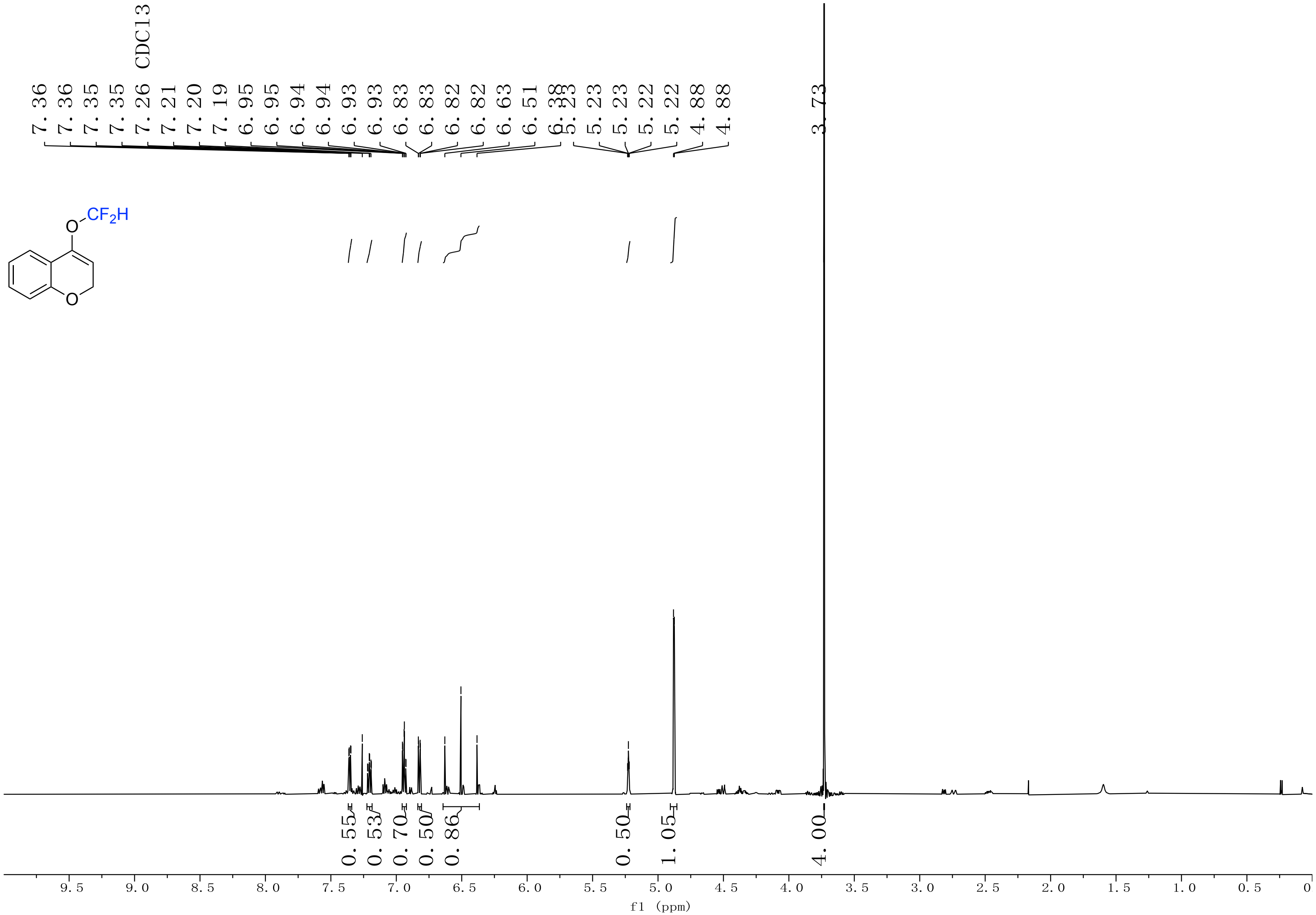

^{19}F NMR of the crude reaction mixture of 4-(difluoromethoxy)-2H-chromene (3m)

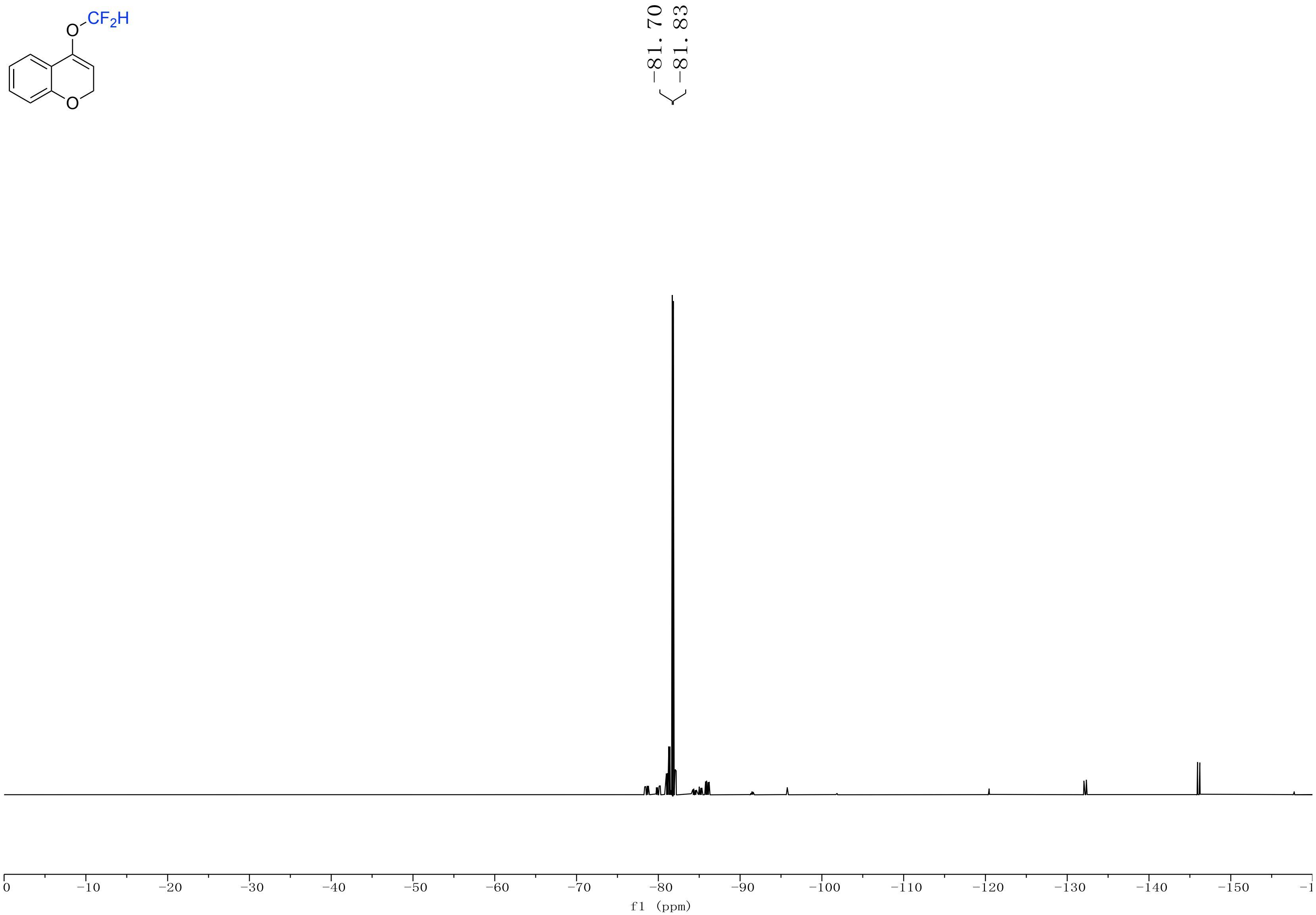

$^{13}C\{^1H\}$ NMR of the crude reaction mixture of 4-(difluoromethoxy)-2H-chromene (3m)

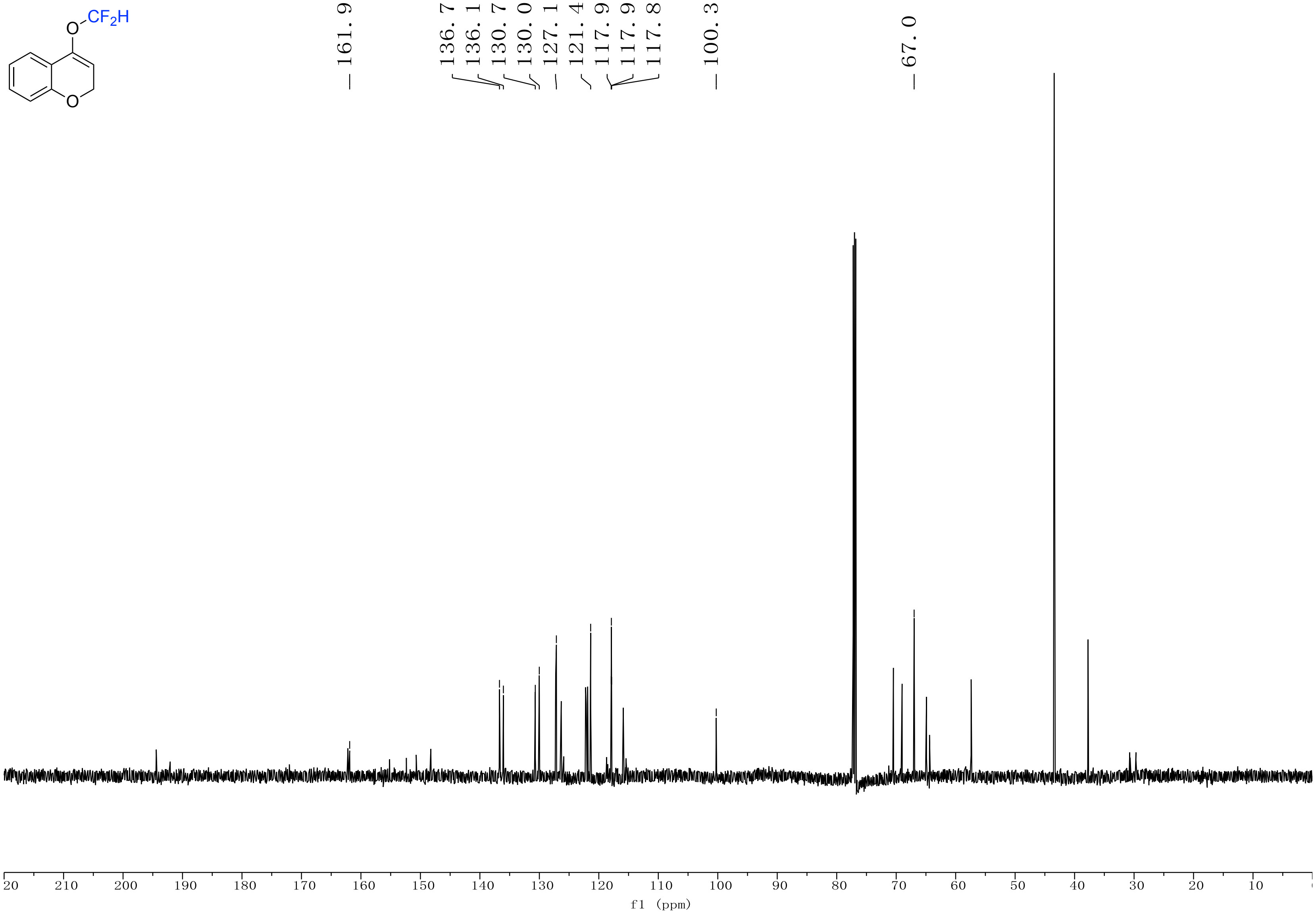

^{1}H NMR of the crude reaction mixture of 1-(difluoromethoxy)-7-phenylcyclohept-1-ene (3n)

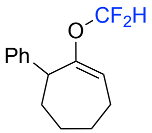

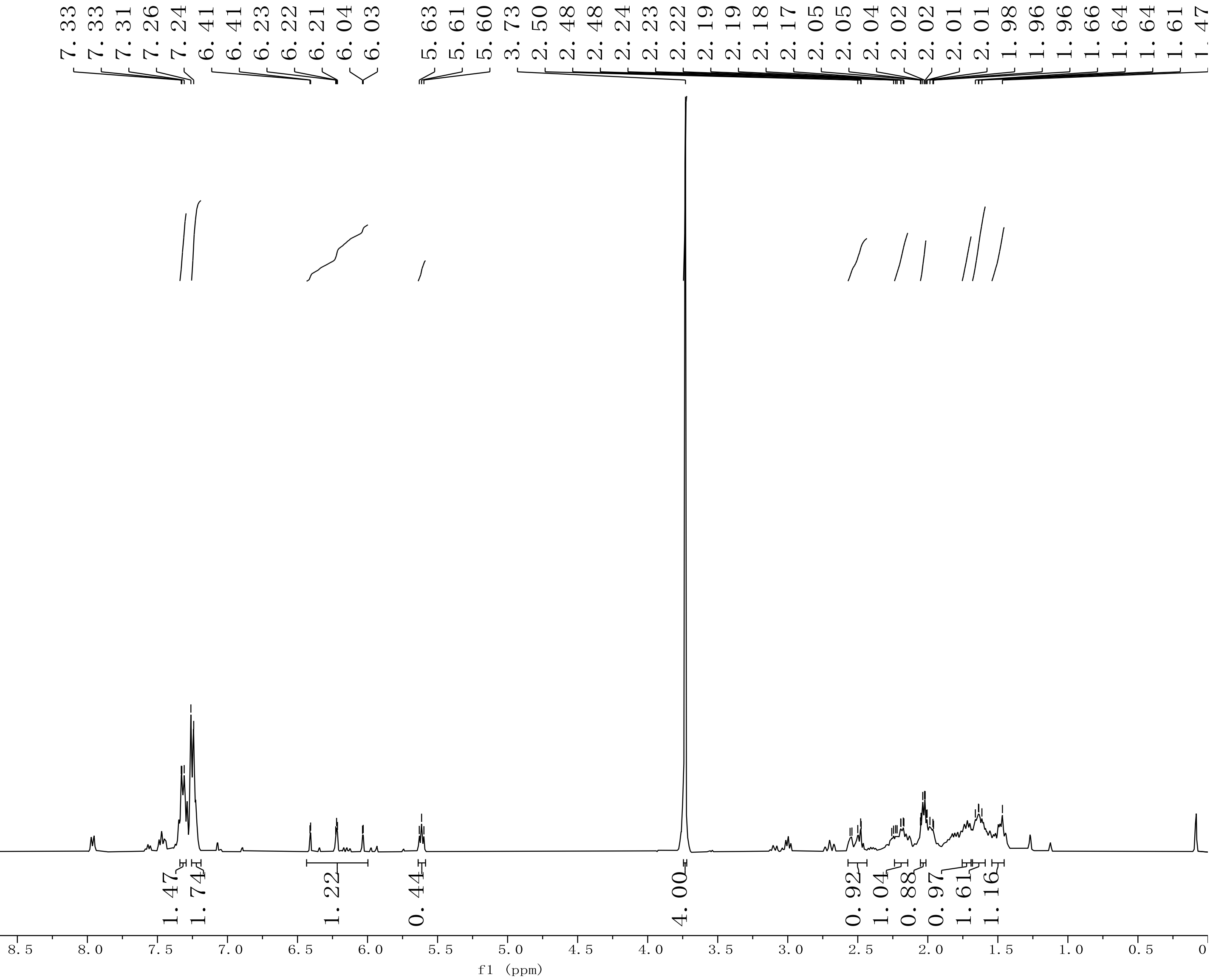

^{19}F NMR of the crude reaction mixture of 1-(difluoromethoxy)-7-phenylcyclohept-1-ene (3n)

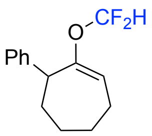

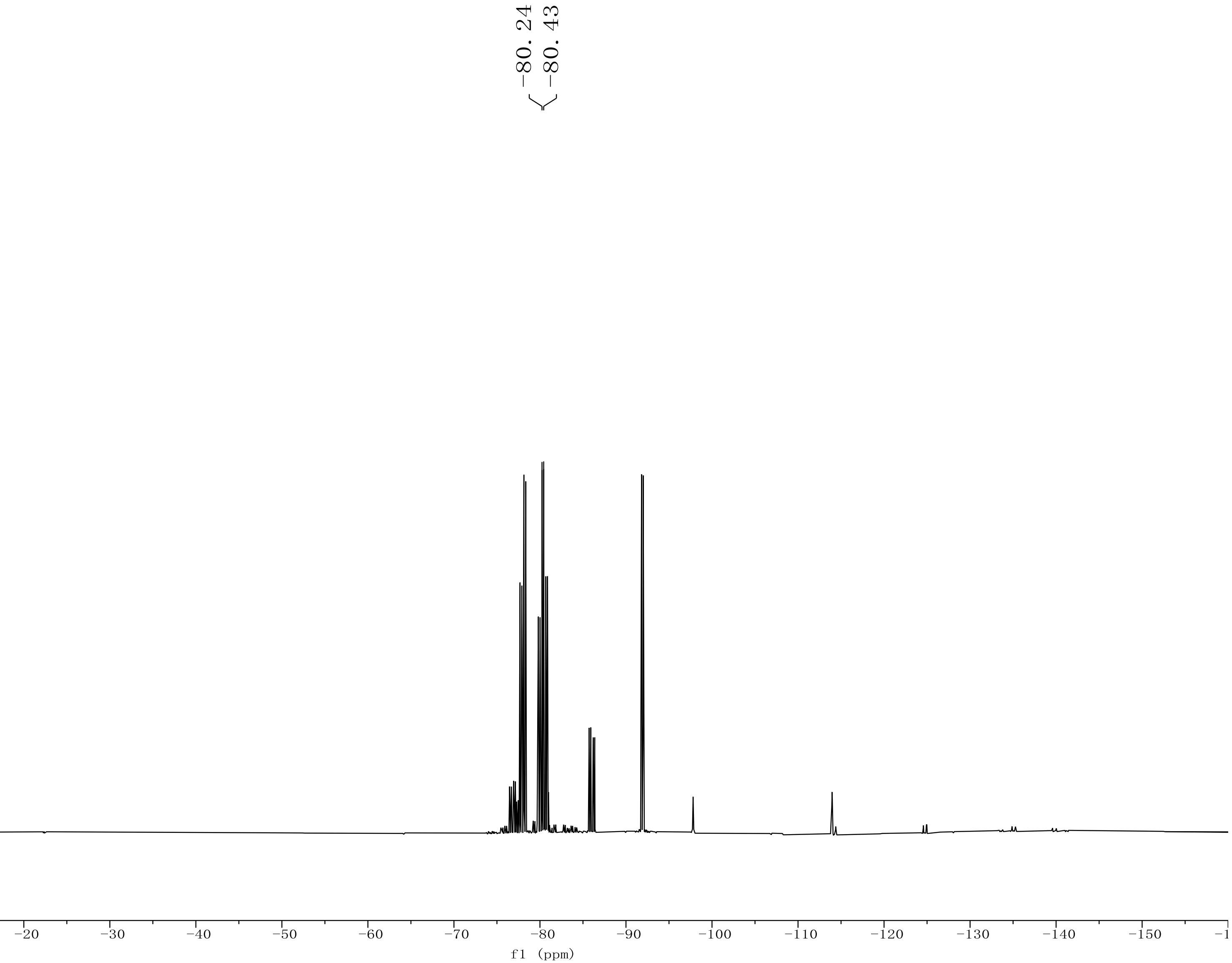

$^{13}C\{^{1}H\}$ NMR of the crude reaction mixture of 1-(difluoromethoxy)-7-phenylcyclohept-1-ene (3n)

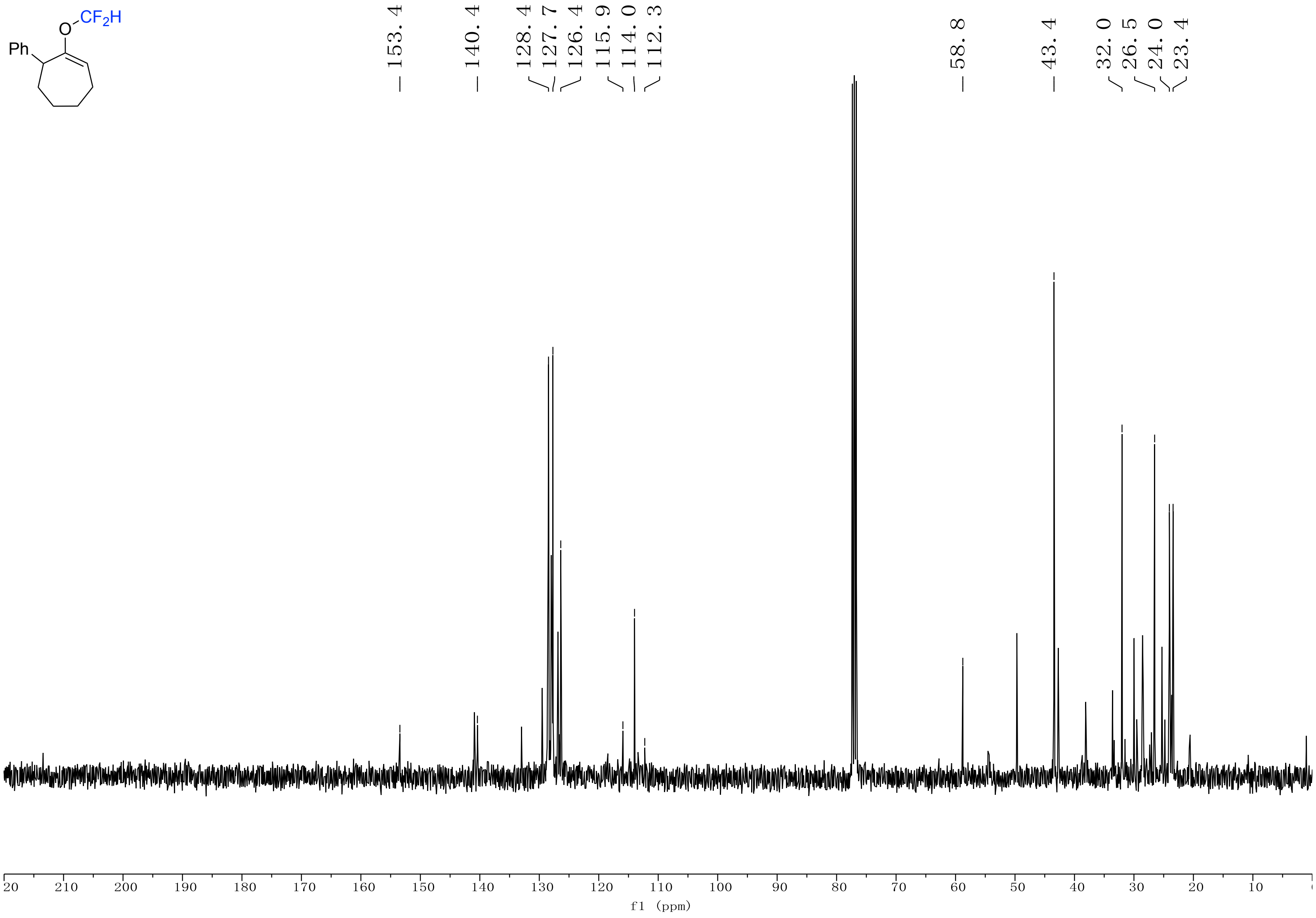

Supplement: File 1 — Experimental procedures, optimization studies, compound characterization data, NMR spectra, and mechanistic investigations. [file Beilstein_J_Org_Chem-20-2799-s001.pdf]
